# Halorotetin B, A Novel Terpenoid Compound Derived from Marine Ascidian, Suppresses Tumor Growth by Targeting the Cell Cycle Regulator UBE2C

**DOI:** 10.1002/advs.202515652

**Published:** 2025-12-12

**Authors:** Shanhao Han, Jianhui Li, Yuting Zhu, Penghui Liu, Yaoyao Zheng, Muchun He, Bo Dong

**Affiliations:** ^1^ Fang Zongxi Center for Marine EvoDevo MoE Key Laboratory of Marine Genetics and Breeding College of Marine Life Sciences Ocean University of China Qingdao 266003 China; ^2^ Laboratory for Marine Biology and Biotechnology Qingdao National Laboratory for Marine Science and Technology Qingdao 266237 China; ^3^ Department of Pharmacy Affiliated Hospital of Shandong University of Traditional Chinese Medicine Jinan 250011 China; ^4^ Liaoning Key Laboratory of Marine Animal Immunology and Disease Control Dalian Ocean University Dalian 116023 China; ^5^ Institute of Evolution & Marine Biodiversity Ocean University of China Qingdao 266003 China

**Keywords:** ascidian, cancer therapy, Halorotetin B, natural compound, UBE2C

## Abstract

Screening and identification of novel small‐molecule have proven to be effective strategies in addressing the growing threat of cancer to human health. In this study, a novel natural terpenoid compound, Halorotetin B, is identified from the edible ascidian *Halocynthia roretzi*. Halorotetin B is shown to significantly inhibit tumor growth both in vitro and in vivo. Mechanistically, the E2 ubiquitin‐conjugating enzyme C (UBE2C) is identified as a direct binding target of Halorotetin B through a combination of the peptide‐centric local stability assay and the omics‐based target enrichment and ranking. Further investigations reveal that Halorotetin B binding to UBE2C induced M phase cell cycle arrest by inhibiting the ubiquitin‐mediated degradation of key cell cycle regulators, including cyclin B1 and securin, ultimately leading to tumor cell senescence. These findings suggest that Halorotetin B, as a novel cell cycle inhibitor targeting UBE2C, holds strong potential for development into ascidian‐derived therapeutics for cancer treatment.

## Graphical abstract

Halorotetin B, a novel small‐molecule terpenoid identified from an edible marine ascidian, exhibits strong anti‐tumor activity both in vitro and in vivo through direct targeting UBE2C to induce tumor cell cycle arrest and then lead tumor cell senescence. As a newly discovered UBE2C inhibitor, Halorotetin B can serve as a novel potential cell senescence inducer for cancer therapy.

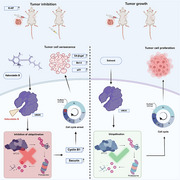

## 1 Introduction

Cancer remains a major threat to human health, ranking as the second leading cause of death globally after cardiovascular disease.^[^ ^1^ ^]^ Despite its high mortality rate, treatment options for cancer are still limited. Currently, small‐molecule drug chemotherapy is an effective strategy for cancer therapy.^[^ ^2^ ^]^ However, there is an urgent and unmet need to develop highly effective anti‐tumor drugs with lower toxicity. An ideal chemotherapeutic drug should exploit vulnerabilities unique to cancer cells that are absent in normal tissues. Natural products have long been recognized as a valuable source in drug discovery, owing to their structural novelty and chemical diversity.^[^ ^3^ ^]^ This is particularly true in the field of cancer chemotherapy, where ≈70% of FDA‐approved anti‐tumor drugs are natural products or their derivatives.^[^ ^4^ ^]^ Covering the majority of the earth's surface and hosting far greater biodiversity than terrestrial ecosystems, the ocean is a rich reservoir of bioactive compounds. The complex and diverse marine environment promotes the production of a wide range of structurally unique natural products. To date, more than 43 000 structurally novel marine natural compounds have been identified (https://marinlit.rsc.org). As a result, marine organisms are increasingly recognized as a promising source of bioactive molecules,^[^ ^5^ ^]^ making the search for marine‐derived natural products an emerging focus in pharmaceutical research.

Marine microorganisms, microalgae, and marine invertebrates are the three primary sources of marine natural products. Among marine invertebrates, ascidians have emerged as important model organisms in developmental biology due to their simple body structure and rapid embryogenesis.^[^ ^6^ ^]^ As a key evolutionary node between invertebrates and vertebrates, ascidians exhibit remarkable species diversity and environmental adaptability. This, along with their symbiotic microorganisms, endows them with the potential to produce a wide range of structurally novel and functionally diverse natural products.^[^ ^7^ ^]^

The first natural product from ascidians was isolated from *Aplidium* sp. in 1967.^[^ ^8^ ^]^ Since then, an increasing number of bioactive compounds have been discovered from ascidians. Notably, two ascidian‐derived secondary metabolites—Dehydrodidemnin B and Ecteinascidin 743 (ET‐743) have received approval for therapeutic use. Dehydrodidemnin B, extracted from *Trididemnum solidum*, was the first marine compound to enter clinical trials and exerts anti‐tumor effects by inhibiting protein biosynthesis.^[^ ^9^ ^]^ ET‐743, derived from *Ecteinascidia turbinata*, exhibits a unique mechanism of action by binding to DNA and blocking the nucleotide excision repair pathway, thereby inducing tumor cell death.^[^ ^10^ ^]^ Interestingly, ET‐743 has been shown to be synthesized not directly by the ascidian but by its symbiotic bacterium, *Candidatus Endoecteinascidia frumentensis*.^[^ ^11^ ^]^ In addition to these examples, many other ascidian‐derived natural products with novel structures and a wide range of biological activities have been reported. Taken together, ascidians represent a rich and underexplored resource for the discovery and development of new bioactive compounds.

*Halocynthia roretzi*, an edible species of ascidian, consists of three main anatomical parts: the stolon, the tunic, and the endocyst. The stolon serves as the organism's anchoring structure in the marine environment, while the endocyst is the edible portion, known for its rich nutritional content.^[^ ^12^ ^]^ Notably, the tunic is composed of cellulose‐a rare feature among animals‐which enhances the organism's adaptability to the complex and variable conditions of marine habitats. Despite the protective nature of its tough tunic, *H. roretzi* relies on secondary metabolites, primarily produced by its symbiotic microorganisms, to defend against marine pathogens. Our previous research has confirmed that *H. roretzi* harbors a diverse community of symbionts capable of producing a wide array of bioactive natural products.^[^ ^13^ ^]^ Other studies have also demonstrated that *H. roretzi*‐derived compounds exhibit a variety of biological activities, including antibacterial,^[^ ^14^ ^]^ anti‐inflammatory,^[^ ^15^ ^]^ and anti‐tumor effects.^[^ ^16^ ^]^

Although numerous natural products have been identified from ascidians, many exhibited poor druggability due to unclear mechanisms of action. Furthermore, research on anti‐tumor compounds from *H. roretzi* remains at a preliminary stage. In this study, we identified a novel small‐molecule terpenoid, Halorotetin B, from *H. roretzi*, which exhibits significant anti‐tumor activity. Importantly, we demonstrated that Halorotetin B directly targets the E2 ubiquitin‐conjugating enzyme UBE2C and elucidated its underlying anti‐tumor mechanisms. By binding to UBE2C, Halorotetin B inhibits the degradation of key cell cycle regulators, cyclin B1 and securin, thereby inducing robust tumor cell cycle arrest. Our findings suggest that Halorotetin B represents a promising UBE2C‐targeting small‐molecule inhibitor with strong potential for development as a therapeutic drug for cancer treatment.

## 2 Results and Discussion

### 2.1 Discovery and Structure Elucidation of Halorotetin B

#### 2.1.1 Discovery of the Anti‐Tumor Small‐Molecule Compound from *Halocynthia roretzi*

**Figure 1:**
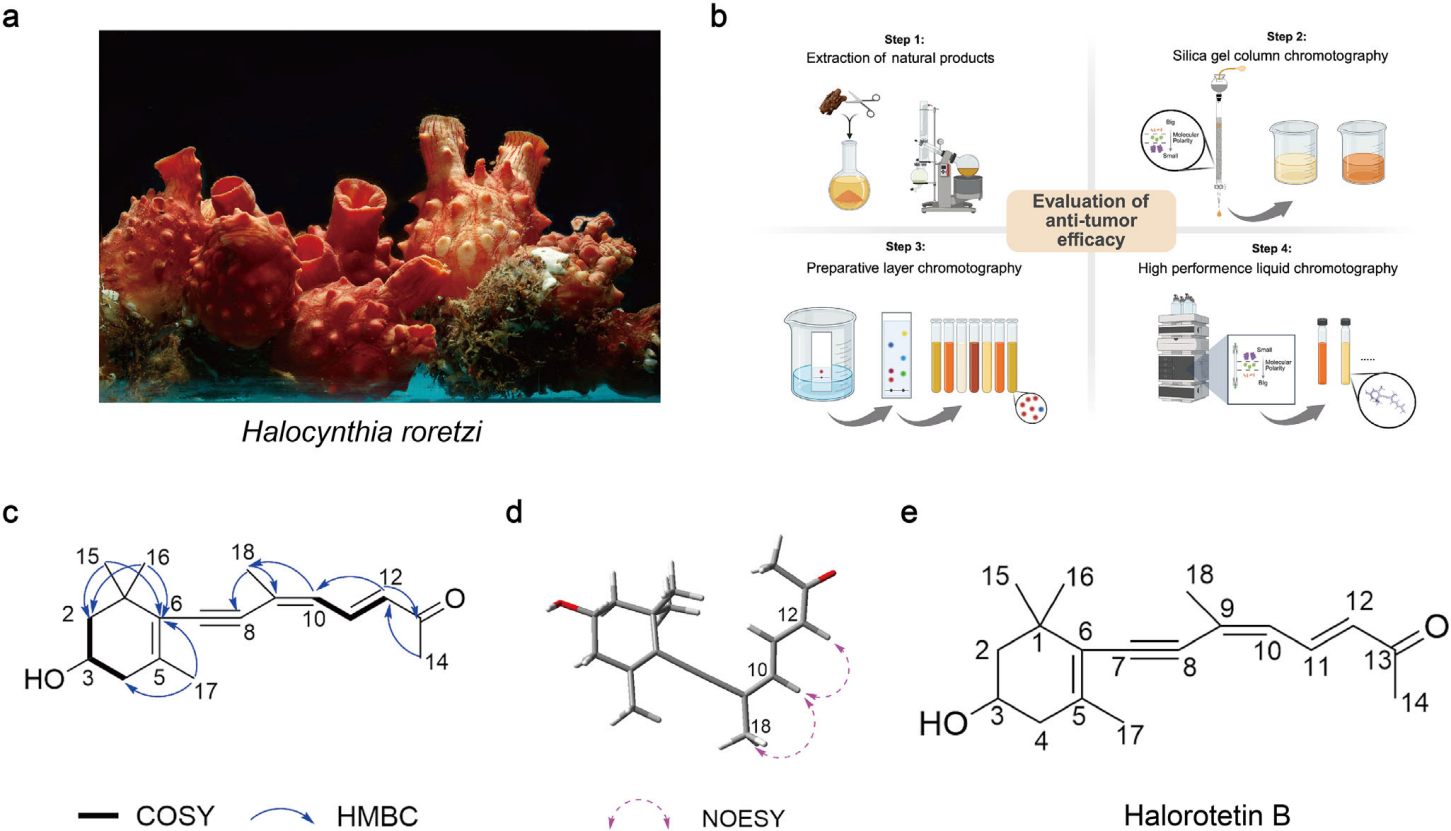
Discovery and structure elucidation of Halorotetin B. a) Adults of *Halocynthia roretzi*. The tunic (Orange‐red part) is composed of cellulose, and the stolon (Brown part in the bottom) plays attachment role in the ocean environment. b) Extraction and isolation processes of Halorotetin B. Step 1 exhibits the extraction of natural products from tunic. Step 2,3,4 exhibits the schematic of silica gel column chromatography, preparative layer chromatography (PLC), and high‐performance liquid chromatography (HPLC), respectively. Each step is accompanied by the evaluation of anti‐tumor efficacy. c) The key COSY, HMBC correlations of Halorotetin B. d) The key NOESY correlations of Halorotetin B. e) The chemical structure of Halorotetin B and the chemical formula of Halorotetin B is C_18_H_24_O_2_.

We isolated natural products with anti‐tumor activity from *H. roretzi* using a tumor cytotoxicity‐guided approach. The adults of the *H. roretzi* were dissected (**Figure** **1**a; Figure S1a, Supporting Information), and then the different parts were infusion with ethyl acetate. Based on preliminary tumor cell cytotoxicity screening, extracts from the tunic of *H. roretzi* were selected for further isolation. Subsequently, a combination of silica gel column chromatography, preparative layer chromatography (PLC), and high‐performance liquid chromatography (HPLC) was employed to purify natural products exhibiting anti‐tumor activity. During the purification process, fractions exhibiting the strongest cytotoxicity against tumor cells were selectively retained (Figure 1b; Figure S1b–e, Supporting Information). As a result, we successfully isolated a highly pure small‐molecule compound from the tunic extracts that exhibited the most potent tumor cell cytotoxicity (Figure S1f, Supporting Information).

#### 2.1.2 Structure Elucidation of the Novel Terpenoid Compound Halorotetin B

To identify the structure of the isolated small‐molecule, we performed nuclear magnetic resonance (NMR) spectroscopy and high‐resolution mass spectrometry (HRESIMS). The ^1^H NMR spectrum and HSQC spectrum revealed that there were five methyl groups, and the methyl group at 2.28 ppm may be attached to the unsaturated carbon. There were also two groups of methylene signals in the high‐field region, and their chemical inequivalence characteristics indicated that the two methylene groups may be in the circular structure. In addition, there was one hypomethyl hydrogen signal near 3.93 ppm, and three aromatic hydrogen or alkene hydrogen signals in the range of 6.00–8.00 ppm in the low‐field region (Figure S2a,d, Supporting Information). The ^13^C NMR spectrum indicated that the compound had 18 carbon atoms including 11 carbons linked to hydrogen and seven quaternary carbons, where a signal at 199.9 ppm suggested the presence of an aldehyde or carbonyl group in the compound. In the COSY spectrum, the correlation between H‐2/3/4 revealed that the compound contains ─CH_2_─CH(OH)─CH_2_─ structural fragments (Figure 1c; Figure S2b,c and Table S1, Supporting Information). Meanwhile, the HMBC correlation signal between CH_3_‐15/CH_3_‐16 and C‐1/2/6 and the HMBC correlation signal between CH_3_‐17 and C‐4/6 suggested that the compound contains cyclohexane partial fragment. In addition, in the COSY spectrum, the correlation between H‐10/11/12 indicated that there were two adjacent double‐bond structures, and the correlation between H‐12, C‐10/13 and H‐14, C‐12/13 in the HMBC spectrum indicated that the methyl C‐14 is linked to an unsaturated carbonyl group and was attached to the C‐12 terminus. The correlation between H‐10 and C‐18, H‐18, and C‐8/9 in the HMBC spectrum further improved the side chain structure. In addition to the quaternary carbon of C‐8, there is also a quaternary carbon signal. Thus, we speculated that C‐7/8 forms an alkyne group that connected the cyclohexane moiety to the side chain moiety (Figure 1c; Figure S2c,e and Table S1, Supporting Information). In the NOESY spectrum, CH_3_‐18 was NOE related to H‐10 but not H‐11, therefore, the double bond between C‐9/10 was Z configuration, and the C‐11/12 double bond was inferred to be E configuration according to the NOE correlation between H‐10 and H‐12 and the large coupling constant between H‐11 and H‐12 (Figure 1d; Figure S2f, Supporting Information). Furthermore, the HRESIMS data confirmed molecular weight of this small‐molecule (Figure S2g, Supporting Information). Based on all above results, the structure of this small‐molecule was determined (Figure 1e). Comparison with the MicroNMR database (https://www.nmrdata.com), we confirmed that the compound is a novel terpenoid, which we named Halorotetin B.

### 2.2 Halorotetin B Presents Inhibition Capacity on Tumor Cell and Tumor Spheroid Growth

**Figure 2:**
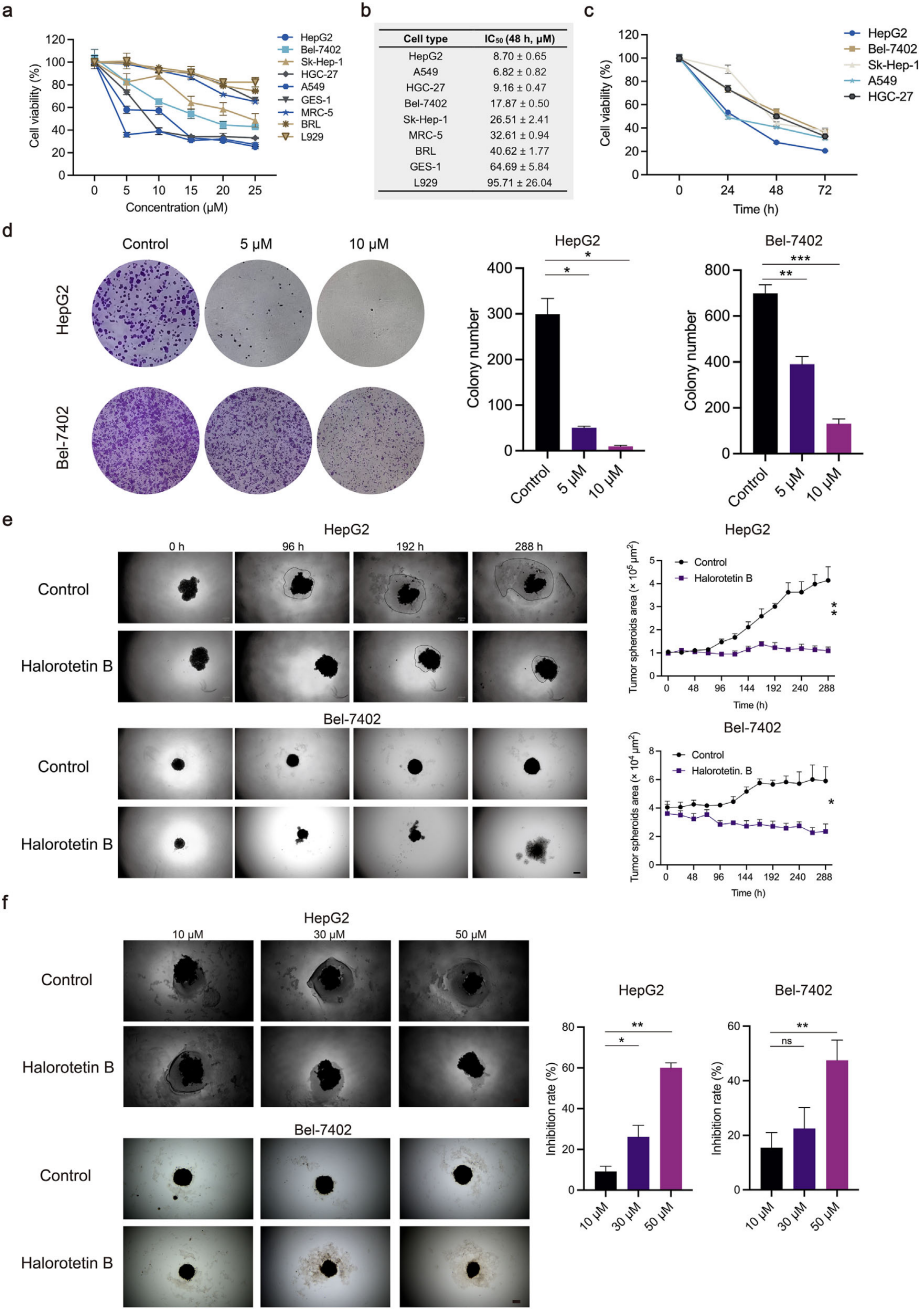
Halorotetin B inhibits the growth of tumor cell and tumor spheroid. a) The inhibitory effects of Halorotetin B on tumor cell lines and non‐malignant cell lines after treatment with Halorotetin B for 48 h. Doxorubicin was used as a positive control. b) The IC_50_ value of Halorotetin B on tumor cell lines and non‐malignant cell lines. c) The inhibitory effects of Halorotetin B on tumor cells after different treatment time. d) Halorotetin B decreases colony formation ability of HCC cells. The cells were stained with crystal violet and quantified. Data are presented as mean ± SD. Significance was determined by one‐way ANOVA, ^*^ *p* <0.05, ^**^ *p* <0.01, ^***^ *p* <0.001, n = 3 biologically independent samples. e) Halorotetin B (60 µm) inhibits the growth of HCC tumor spheroids, control group was the same volume of methanol as 60 µM Halorotetin B, scale bar = 200 µm. Data are presented as mean ± SD. Significance was determined by two‐way ANOVA, ^*^ *p* <0.05, ^**^ *p* <0.01, n = 3 biologically independent samples. f) Halorotetin B inhibited the proliferation of tumor spheroids in a dose‐dependent manner. In the statistical data, the treatment time with Halorotetin B was 288 h, scale bar = 200 µm. Data are presented as mean ± SD. Significance was determined by one‐way ANOVA, ns, *p* >0.05, ^**^ *p* <0.01, n = 3 biologically independent samples.

To assess its in vitro anti‑tumor activity, various cancer cell lines were treated with Halorotetin B. The results showed that Halorotetin B inhibited the growth of several types of tumor cells in a dose‐dependent manner (**Figure** **2a**). Its IC_50_ value for different tumor cells showed the high inhibition efficacy (Figure 2b; Figure S3a, Supporting Information). It is worth noting that non‐malignant cell lines were less sensitive to Halorotetin B than tumor cell lines (Figure 2a,b). Additionally, the inhibitory effect of Halorotetin B on the tested tumor cells was found to be time‐dependent (Figure 2c). Next, we also assessed the cytotoxicity of Halorotetin B to other tumor cells and found that the hepatocellular carcinoma (HCC) cells were most sensitive to Halorotetin B among all the tested cell lines (Figure S3b,c, Supporting Information). Therefore, the HCC cell lines were selected for further experiments. To further assess the growth inhibition of Halorotetin B on HCC cells, the colony formation assay was performed. The results showed that Halorotetin B treatment significantly decreased the colony formation ability of HCC cells (Figure 2d; Figure S3d, Supporting Information). In addition, to detect the anti‐tumor efficacy of Halorotetin B different from the cellular level, hepatocellular carcinoma tumor spheroids were established and treated with either Halorotetin B or methanol (control). The size of the tumor spheroids was then assessed using optical microscopy. The results showed that Halorotetin B treatment significantly reduced the growth of HepG2‐derived tumor spheroids (Figure 2e). Similarly, Halorotetin B significantly inhibited the growth of tumor spheroids derived from Bel‑7402 and Huh‑7 cells (Figure 2e; Figure S3e, Supporting Information). To further investigate whether Halorotetin B had a dose‐dependent inhibitory effect on the proliferation of hepatocellular carcinoma tumor spheroids, we treated tumor spheroids with different concentrations of Halorotetin B. The results indicate that the inhibitory effects of Halorotetin B on tumor spheroid proliferation is in a dose‐dependent manner (Figure 2f; Figure S3f, Supporting Information).

### 2.3 Halorotetin B Suppresses Tumor Growth in vivo

**Figure 3:**
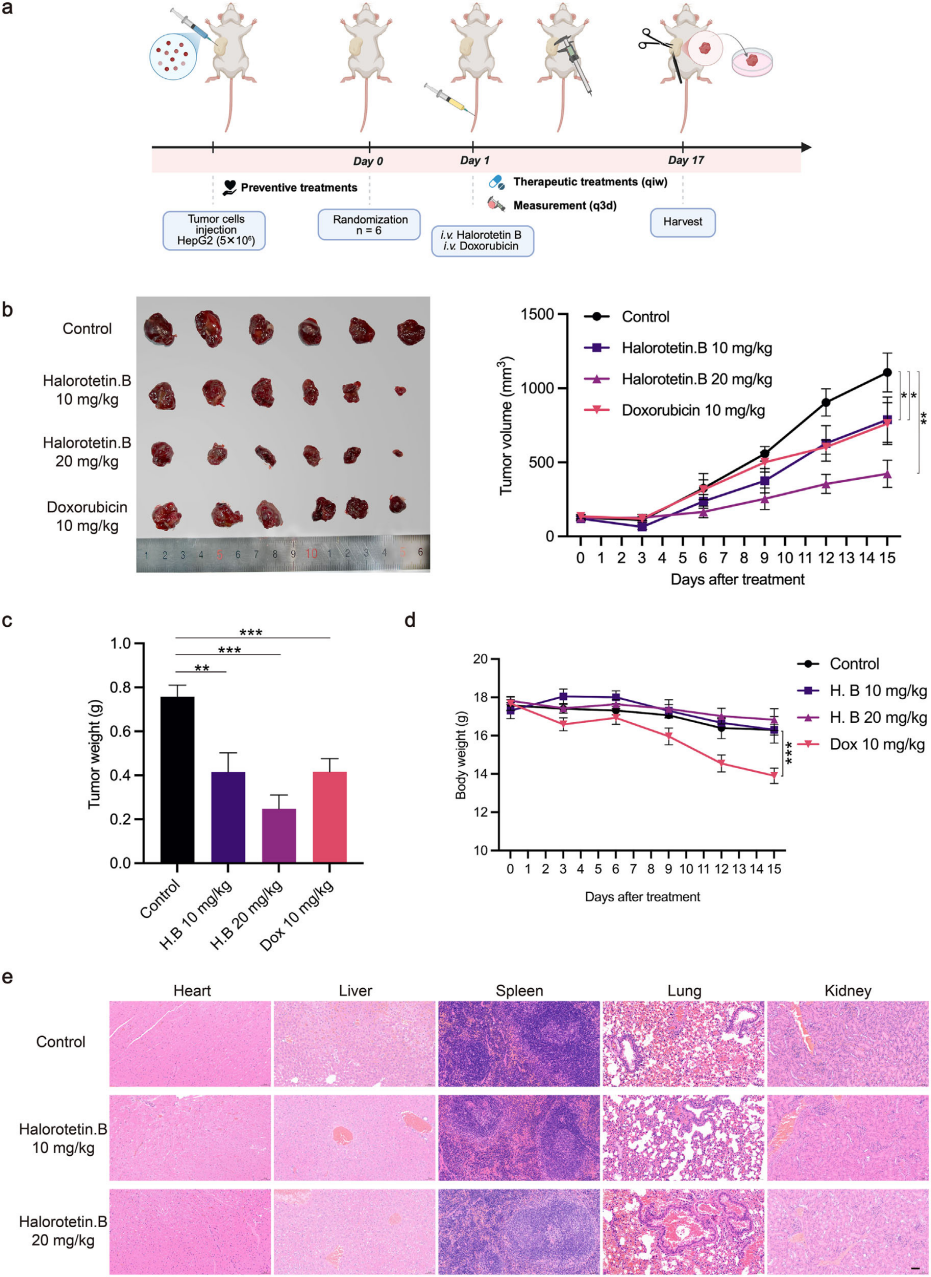
Halorotetin B inhibits tumor progression. a) Schematic outline of the HepG2 xenograft mouse model, and the solvent, Doxorubicin, Halorotetin B were given by tail intravenous injection. b) The tumor images and growth curve of HepG2 xenografts treated with solvent (Control), Doxorubicin and Halorotetin B, the tumor volume were detected every three days. Data are presented as mean ± s.e.m. Significance was determined by two‐way ANOVA, ^*^ *p* <0.05, ^**^ *p* <0.01, n = 6 mice. c) Tumor weight of xenograft models. Data are presented as mean ± s.e.m. Significance was determined by one‐way ANOVA, ^**^ *p* <0.01, ^***^ *p* <0.001, n = 6 mice. d) Body weight of xenograft models, the body weight was detected every three days. Data are presented as mean ± s.e.m. Significance was determined by two‐way ANOVA, ^***^ *p* <0.001, n = 6 mice. **e**) Representative hematoxylin and eosin staining images of vital organs, no histological changes were observed in these organs, scale bar = 50 µm.

To further investigate the anti‐tumor effects of Halorotetin B in vivo, we established hepatocellular carcinoma tumor model by injecting HepG2 cells into immunodeficient mice (**Figure** **3**a). Upon tail intravenous injection of either the solvent (control) or Halorotetin B, the control group exhibited rapid tumor growth, while tumor growth was significantly inhibited in the Halorotetin B‐treated group (Figure 3b). Furthermore, the tumor weight was also significantly decreased after Halorotetin B treatment (Figure 3c). To assess the potential in vivo side effects of Halorotetin B, we monitored the body weight of all mice every three days. The result showed that there was no significant difference in body weight between control and Halorotetin B treatment groups, whereas the Doxorubicin‐treated groups showed a significant decreasing trend (Figure 3d). Moreover, the vital organs, including the heart, liver, spleen, lungs, and kidneys were also harvested from the mice for histological examination. The results revealed no abnormalities in Halorotetin B–treated mice compared to controls, indicating that Halorotetin B causes no significant host toxicity at its effective dose (Figure 3e; Figure S4, Supporting Information). Together, these results demonstrate that Halorotetin B delivers potent anti‑tumor efficacy in vivo without adverse effects.

### 2.4 Halorotetin B Induces Cell Cycle Arrest of Tumor Cells

**Figure 4:**
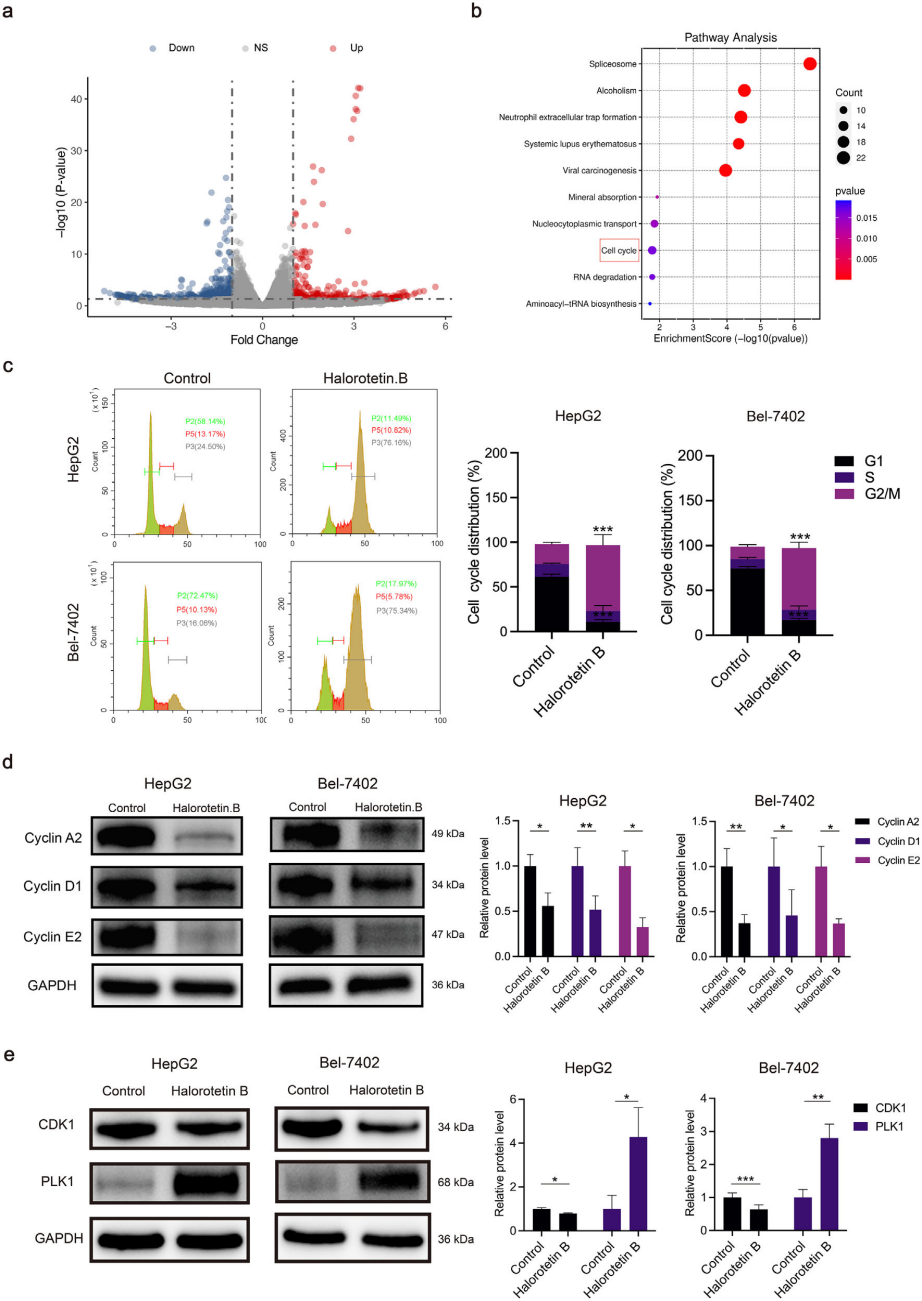
Halorotetin B induces cell cycle arrest of tumor cells. a) Volcano plot showed the differentially expressed genes between Halorotetin B (7 µm) treated and untreated HepG2 cells. Treatment time was 24 h. b) KEGG analysis for the significantly differentially expressed genes, the top ten pathways were listed. c) The cell cycle distribution was analyzed by flow cytometry, Halorotetin B concentration: 20 µm, and Halorotetin B treatment time: 24 h. Data are presented as mean ± SD. Significance was determined by one‐way ANOVA, ^***^ *p* <0.001, n = 3 biologically independent samples. d) Western blot analysis of the expression level of G1 or S phase cell cycle‐associated proteins in HCC cells exposed to Halorotetin B (20 µm) treatment for 24 h. Data are presented as mean ± SD. Significance was determined by two‐tailed *t*‐test, ^*^ *p* <0.05, ^**^ *p* <0.01, ^***^ *p* <0.001, n = 3 biologically independent samples. e) Western blot analysis of the expression level of G2 or M phase cell cycle‐associated proteins in HCC cells exposed to Halorotetin B (20 µm) treatment for 24 h. Data are presented as mean ± SD. Significance was determined by two‐tailed *t*‐test, ^*^ *p* <0.05, ^**^ *p* <0.01, ^***^ *p* <0.001, n = 3 biologically independent samples.

To elucidate the underlying mechanisms of Halorotetin B's anti‐tumor effect, we first performed RNA sequencing on HepG2 cells following Halorotetin B treatment. After screening, a total of 393 differentially expressed genes (DEGs) were identified, including 127 upregulated and 266 downregulated genes following Halorotetin B treatment (**Figure 4**a). KEGG analysis revealed that DEGs were mainly enriched in cell cycle signaling pathway, indicating that cell cycle signaling might be the potential target pathway of Halorotetin B (Figure 4b). Subsequently, we examined the effects of Halorotetin B on cell cycle of HCC cells by using flow cytometry, and the results showed the G2/M phases of HCC cells were significantly increased (Figure 4c; Figure S5a, Supporting Information). Furthermore, cell cycle‐ associated proteins were detected after Halorotetin B treatment, including cyclin A2, cyclin D1, and cyclin E2, which were expressed in S phase or G1 phase. The results showed that Halorotetin B treatment significantly decreased the expression of cyclin A2, cyclin D1, and cyclin E2 in three HCC cell lines (Figure 4d; Figure S5b, Supporting Information). Since Halorotetin B induced G2/M phase cell cycle arrest in tumor cells, we also examined its effects on the expression of key regulatory proteins during G2 and M phases. The results showed that the expression of CDK1, a G2‐phase regulatory protein, was significantly decreased after treatment with Halorotetin B, while the expression of PLK1, an M‐phase regulatory protein, was markedly increased (Figure 4e; Figure S5c, Supporting Information). Based on these findings, we speculated that Halorotetin B may cause cell cycle arrest specifically at the M phase. Taken together, these results suggested that Halorotetin B disrupted the cell cycle, and induced G2/M cell cycle arrest of HCC cells.

### 2.5 Identification of UBE2C as a Direct Target of Halorotetin B

**Figure 5:**
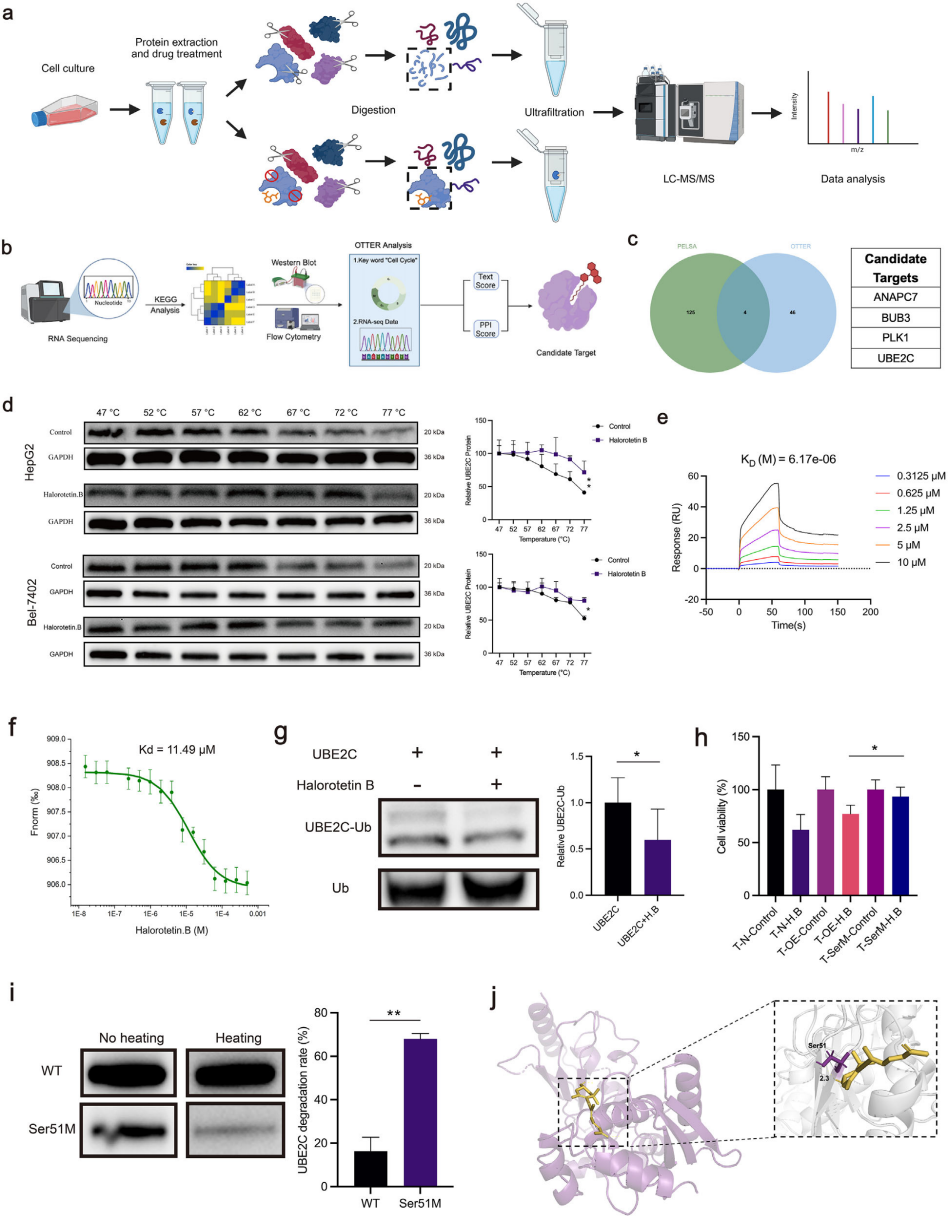
UBE2C is a direct target of Halorotetin B. a) The workflow of peptide‐centric local stability assay (PELSA). For drug treatment, the HepG2 cells were treated with 20 µM Halorotetin B. b) The RNA‐Seq data and key word were submitted to the OTTER and predicated the most potential target proteins. c) Identification of the most potential targets of Halorotetin B by integrated PELSA and OTTER analyses. d) The CETSA assay determined the thermal stabilization of UBE2C interaction with Halorotetin B (20 µM) in HCC cells. Halorotetin B treatment time: 24 h. Data are presented as mean ± SD. Significance was determined by two‐way ANOVA, ^*^ *p* <0.05, ^**^ *p* <0.01, n = 3 biologically independent samples. e) SPR analysis of Halorotetin B binding to recombinant human UBE2C (K_D_ = 6.71 µm). f) MST analysis of Halorotetin B binding to recombinant human UBE2C (K_d_ = 11.49 µM). g) The enzymatic activity detection of UBE2C protein. Ubiquitin chain formation was visualized by Western blot, and the band intensity was quantified by grayscale analysis. Data are presented as mean ± SD. Significance was determined by two‐tailed *t*‐test, ^*^ *p* <0.05, n = 3 biologically independent samples. h) The inhibitory effects of Halorotetin B on HEK‐293T cells after treatment with 20 µm Halorotetin B for 24 h with the transfection of different plasmids. T‐N: HEK‐293T cells without plasmid transfection. T‐OE: HEK‐293T cells with UBE2C overexpression plasmid transfection. T‐SerM: HEK‐293T cells with Ser51 mutation plasmid transfection. Data are presented as mean ± SD. Significance was determined by two‐way ANOVA, ^*^ *p* <0.05, n = 3 biologically independent samples. i) The thermal stabilization of wide type UBE2C and Ser51‐mutant UBE2C. The heating groups were heated at 77 °C for 10 min. Data are presented as mean ± SD. Significance was determined by two‐tailed *t*‐test, ^**^ *p* <0.01, n = 3 biologically independent samples. j) Molecular docking of Halorotetin B (Color in yellow‐orange) to UBE2C (PDB id: 4YII). Representation of Halorotetin B as a stick model showing the position of interaction site with Ser‐51(Color in purple) by Hydrogen bond (Color in yellow).

To investigate the molecular mechanisms by which Halorotetin B regulates the cell cycle signaling pathway, the peptide‐centric local stability assay (PELSA) and the text‐mining‐based web server OTTER were performed to identify the binding proteins of Halorotetin B (**Figure** **5**a,b).^[^ ^17^, ^18^ ^]^ Owing to its superior identification accuracy, PELSA analysis yielded 1463 potential targets proteins. Subsequent Gene Ontology (GO) enrichment analysis of these candidates identified 129 targets associated with the cell cycle signaling pathway. By integrating these findings with predictions from OTTER, we ultimately pinpointed most potential candidate target proteins for Halorotetin B (Figure 5c). To validate the potential targets, we employed the cellular thermal shift assay (CETSA) to screen candidate proteins. The results revealed that UBE2C exhibited significant thermal stabilization upon Halorotetin B treatment compared to the other three proteins (Figure 5d; Figure S6a,b, Supporting Information), indicating a direct interaction between Halorotetin B and UBE2C. Next, to obtain direct evidence of target engagement in a physiologically relevant context, we applied CETSA to tumor tissues isolated from animal models. The thermal stabilization of UBE2C protein was significantly increased in the tumors from Halorotetin B‐treated animals compared to the control group, which indicated that Halorotetin B binds to UBE2C protein in vivo (Figure S6c, Supporting Information). Furthermore, surface plasmon resonance (SPR) and microscale thermophoresis (MST) analysis confirmed that Halorotetin B directly binds to UBE2C, with the dissociation constant values of 6.71 and 11.49 µm, respectively (Figure 5e,f). To investigate whether the binding of Halorotetin B to UBE2C protein affects its enzymatic activity, we conducted a functional assay. Given that UBE2C is an E2 ubiquitin‐conjugating enzyme, we compared the ubiquitin chain formation capability of the wild‐type UBE2C and Halorotetin B‐binding UBE2C. The results demonstrated that Halorotetin B significantly suppressed the enzymatic activity of UBE2C protein (Figure 5g).

Next, we further investigated the specific binding sites of Halorotetin B on UBE2C. The binding mode was predicted by molecular docking tool (AutoDockTools, Version 1.5.7) and visualized by PyMOL (PyMOL Molecular Graphics System, Version 2.5.4). Among the top ten docking models with the smallest docking energy, only six docking models formed docking sites (Figure S6d, Supporting Information). Then the GFP‐tagged mutation plasmids based on these sites were constructed. A GFP‐tagged UBE2C was also constructed as the control group. Next, we transfected HEK‐293T cells with the plasmids encoding wild‐type or mutant UBE2C, followed by treatment with Halorotetin B. As expected, the Ser51‐to‐cysteine mutation exhibited reduced cytotoxicity compared to the wild‐type UBE2C and the other five site‐specific mutations, which did not significantly affect the cytotoxic response to Halorotetin B (Figure 5h; Figure S6e,f, Supporting Information). Similar results were observed when wild‐type and Ser51‐mutant UBE2C plasmids were co‐transfected into HepG2 cells (Figure S6g, Supporting Information). To further confirm the binding site of Halorotetin B on UBE2C, we purified the Ser51‐mutant protein and conducted the CETSA experiment. The results indicated that the thermal stabilization of Ser51‐mutant UBE2C was markedly reduced compared to wild type UBE2C protein (Figure 5i; Figure S6h, Supporting Information). Taken together, these findings suggested that UBE2C was a direct target of Halorotetin B, and the Ser51 was the binding site of Halorotetin B on UBE2C (Figure 5j).

### 2.6 UBE2C is Required in Tumor Progression

**Figure 6:**
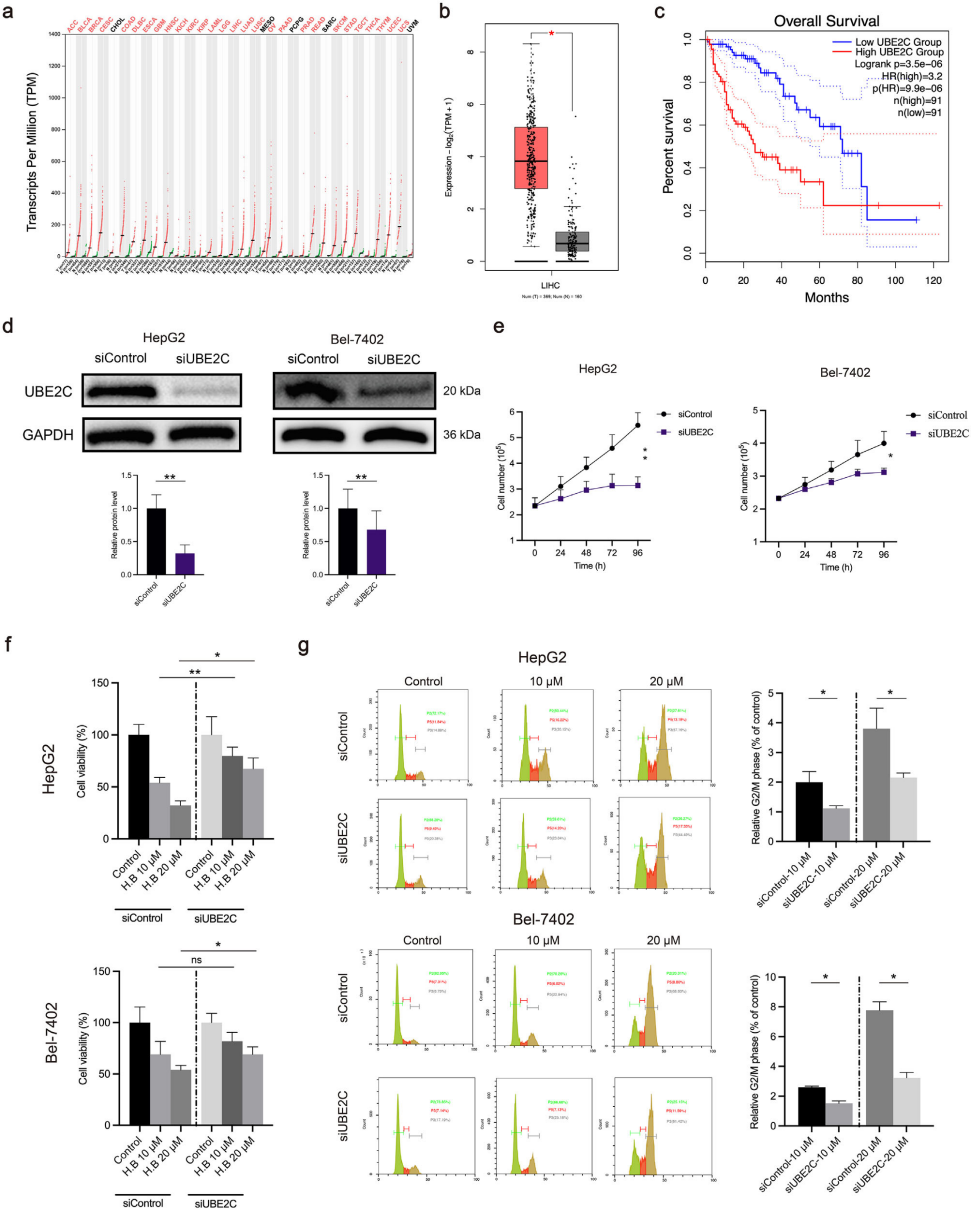
UBE2C overexpression is associated with tumor progression and anti‐tumor efficacy of Halorotetin B. a) The *UBE2C* transcription expression profile across all tumor samples and the paired normal tissues. b) The *UBE2C* expression analysis in liver cancer (LIHC). The method for discrepancy analysis is one‐way ANOVA, using disease state (Tumor or Normal) as variable for calculating differential expression. c) Survival analysis for the association of UBE2C expression with overall survival of patients in the GEPIA2 dataset (http://gepia2.cancer‐pku.cn/#index). d) The protein level of UBE2C after siRNA treatment for 48 h. The concentration of control siRNA and UBE2C siRNA: 100 pm. Data are presented as mean ± SD. Significance was determined by two‐tailed *t*‐test, ^**^ *p*<0.01, n = 3 biologically independent samples. e) Influence of siRNA knockdown of UBE2C on HCC cell proliferation. Data are presented as mean ± SD. Significance was determined by two‐way ANOVA, ^*^ *p*<0.05, ^**^ *p*<0.01, n = 3 biologically independent samples. f) Cell viability assays in HCC cells transfected with siRNA targeting UBE2C or control siRNA. Halorotetin B treatment time: 48 h. Methanol as the control group. Data are presented as mean ± SD. Significance was determined by two‐way ANOVA, ns *p* >0.05, ^*^ *p* <0.05, ^**^ *p* <0.01, n = 3 biologically independent samples. g) Cell cycle distribution assays of Halorotetin B on UBE2C wild type cells and UBE2C knockdown cells. The cells were treated with control under the indicated concentrations of Halorotetin B for 24 h. Methanol as the control group. Data are presented as mean ± SD. Significance was determined by two‐way ANOVA, ^*^ *p* <0.05, n = 3 biologically independent samples.

To explore the role of *UBE2C* in tumor progression, we examined its expression levels in tumor samples using the gene expression profiling interactive analysis 2 (GEPIA2) platform.^[^ ^19^ ^]^ Analysis of the GEPIA2 database revealed that *UBE2C* transcription levels were significantly elevated across various tumor types (**Figure** **6**a). More importantly, overexpression of *UBE2C* in HCC was correlated with the poor survival rate (Figure 6b,c). We further investigated the role of *UBE2C* in tumor cells by performing gene knockdown in HCC cells. Western blot results confirmed a marked reduction at UBE2C protein levels (Figure 6d; Figure S7a, Supporting Information), and the significant decreases in HCC cell proliferation were observed in the UBE2C knockdown groups (Figure 6e; Figure S7b, Supporting Information). Moreover, UBE2C knockdown in HCC cells markedly reduced the cytotoxicity of Halorotetin B compared to the wild‐type HCC cells (Figure 6f; Figure S7c, Supporting Information). Silencing *UBE2C* also led to an accumulation of HCC cells in the G2/M phase and attenuated Halorotetin B‐induced cell cycle arrest (Figure 6g; Figure S7d, Supporting Information). Together, these findings demonstrate that *UBE2C* is critical for tumor progression and is required for the anti‑tumor effects of Halorotetin B.

### 2.7 Halorotetin B Arrests Tumor Cells at Cell Cycle M Phase

**Figure 7:**
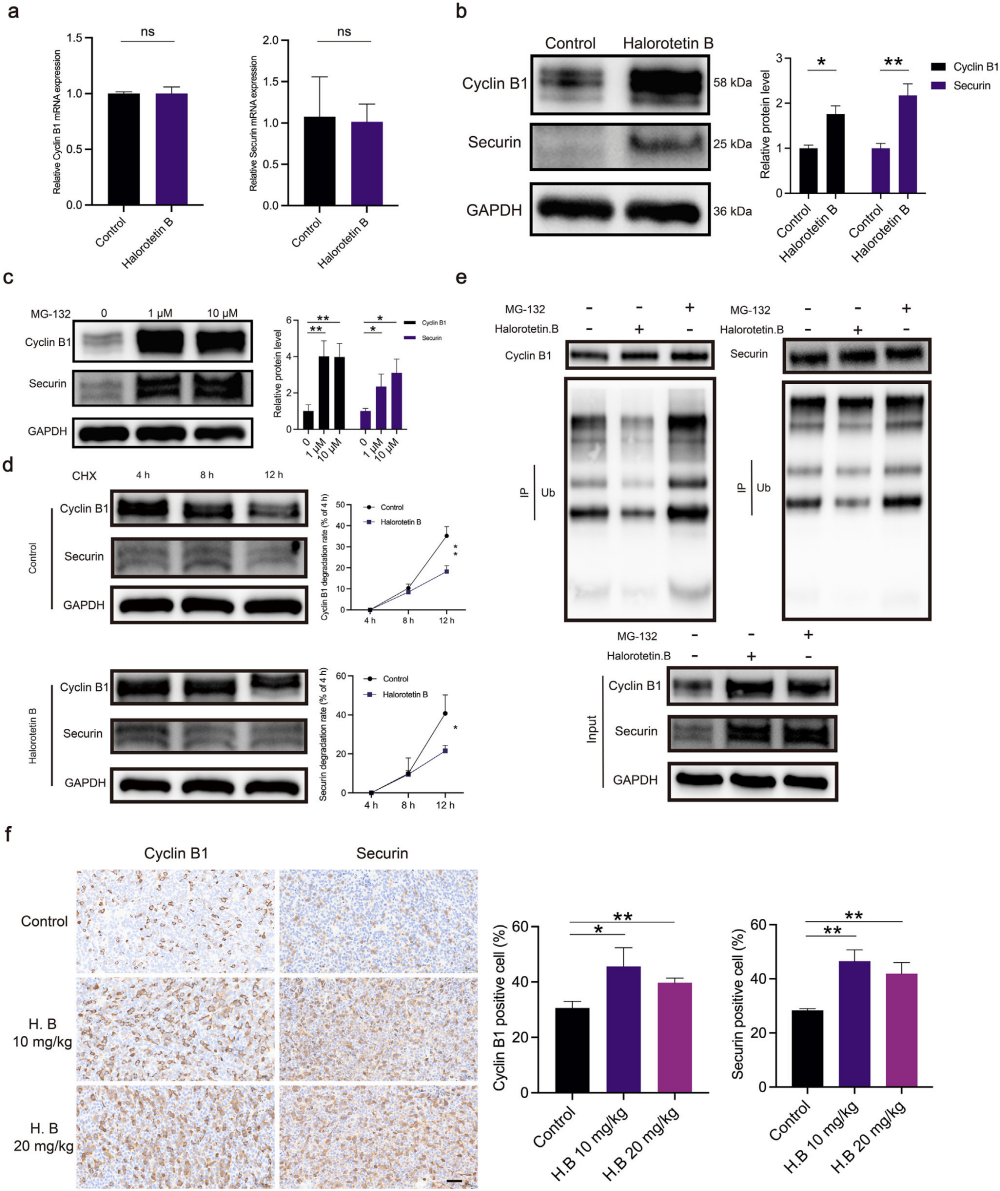
Halorotetin B arrests tumor cells at the cell cycle M phase. a) Quantitative RT‐PCR was used to analyze the mRNA level of *cyclin B1* and *securin* in HepG2 cells treated with Halorotetin B (20 µm) for 24 h. Data are presented as mean ± SD. Significance was determined by two‐tailed *t*‐test, ^*^ *p* <0.05, ^**^ *p* <0.01, n = 3 biologically independent samples. b) Western blot analyzed the protein level of cyclin B1 and securin in HepG2 cells treated with Halorotetin B (20 µm) for 24 h. Data are presented as mean ± SD. Significance was determined by two‐tailed *t*‐test, ns *p* >0.05, n = 3 biologically independent samples. c) The influence of ubiquitin‐proteasome pathway on the degradation of cyclin B1 and securin in HepG2 cells was evaluated by proteasome inhibitor (MG‐132), and quantification was determined by the protein level. Data are presented as mean ± SD. Significance was determined by one‐way ANOVA, ^*^ *p* <0.05, ^**^ *p* <0.01, n = 3 biologically independent samples. d) The degradation rate of cyclin B1 and securin in HepG2 cells was analyzed by Western blot in the presence of CHX (50 mgmL^−1^). Data are presented as mean ± SD. Significance was determined by two‐way ANOVA, ^*^ *p* <0.05, ^**^ *p* <0.01, n = 3 biologically independent samples. e) Immunoblotting presents the ubiquitination level of degradation rate of cyclin B1 and securin in HepG2 cells with or without Halorotetin B (20 µm) treatment for 24 h. f) Representative images of cyclin B1 and securin immunohistochemical staining of tumor tissues after treatment with Halorotetin B or solvent, scale bar = 50 µm. The number of cyclin B1 and securin positive cells was counted in the whole section. Data are presented as mean ± SD. Significance was determined by one‐way ANOVA, ^*^ *p* <0.05, ^**^ *p* <0.01, n = 3 sections per group.

UBE2C as an E2 ubiquitin‐conjugating enzyme, plays important roles in the delivery of ubiquitin and degradation of target proteins.^[^ ^20^ ^]^ During the M phase of cell mitosis, the cyclin B1 and securin are the most important target proteins of UBE2C.^[^ ^21^ ^]^ The ubiquitination degradation of these two proteins is necessary for the exit of cell mitosis.^[^ ^22^ ^]^ We herein investigated whether Halorotetin B inhibit the degradation of cyclin B1 and securin via targeting UBE2C. Quantitative Real‐time PCR assays revealed that Halorotetin B had no significant effect on mRNA level of *cyclin B1* and *securin* in HepG2 cells (**Figure** **7**a). However, further experiments demonstrated that the protein level of cyclin B1 and securin was significantly increased in HepG2 cells after Halorotetin B treatment (Figure 7b). To investigate the influence of ubiquitin‐proteasome pathway on the degradation of cyclin B1 and securin, we treated HepG2 cells with proteasome inhibitor (MG‐132) and found that the protein level of cyclin B1 and securin was significantly increased (Figure 7c). These results suggested that the protein levels of cyclin B1 and securin were regulated by the ubiquitin‐proteasome pathway and were influenced by Halorotetin B treatment. To determine whether Halorotetin B modulates cyclin B1 and securin levels via the ubiquitin–proteasome pathway, HepG2 cells were treated with the protein synthesis inhibitor cycloheximide (CHX). In the presence of CHX, the degradation rates of both cyclin B1 and securin became slower in Halorotetin B–treated cells than the control ones (Figure 7d). Moreover, the ubiquitination levels of cyclin B1 and securin in HepG2 cells were significantly decreased in Halorotetin B treatment group compared to the control group and MG‐132 treatment group (Figure 7e).

More importantly, immunohistochemistry (IHC) and western blot assays revealed that the protein levels of cyclin B1 and securin were also significantly increased in mouse tumor tissues (Figure 7f; Figure S8a, Supporting Information). Interestingly, the Ki‐67 marker, which is crucial for assessing tumor proliferation, also showed an increased positive rate following Halorotetin B treatment (Figure S8b, Supporting Information). This is because the expression of Ki‐67 spans the whole cell cycle.^[^ ^23^ ^]^ Halorotetin B can arrest the cell cycle at the M phase, causing the cells to synchronize, and these synchronized cells still express Ki‐67, resulting in an increased positivity rate of Ki‐67 during IHC staining. All together, these results indicate that Halorotetin B induces M phase cell cycle arrest by inhibiting the ubiquitination degradation of cyclin B1 and securin.

### 2.8 Halorotetin B Induces Tumor Cell Senescence

**Figure 8:**
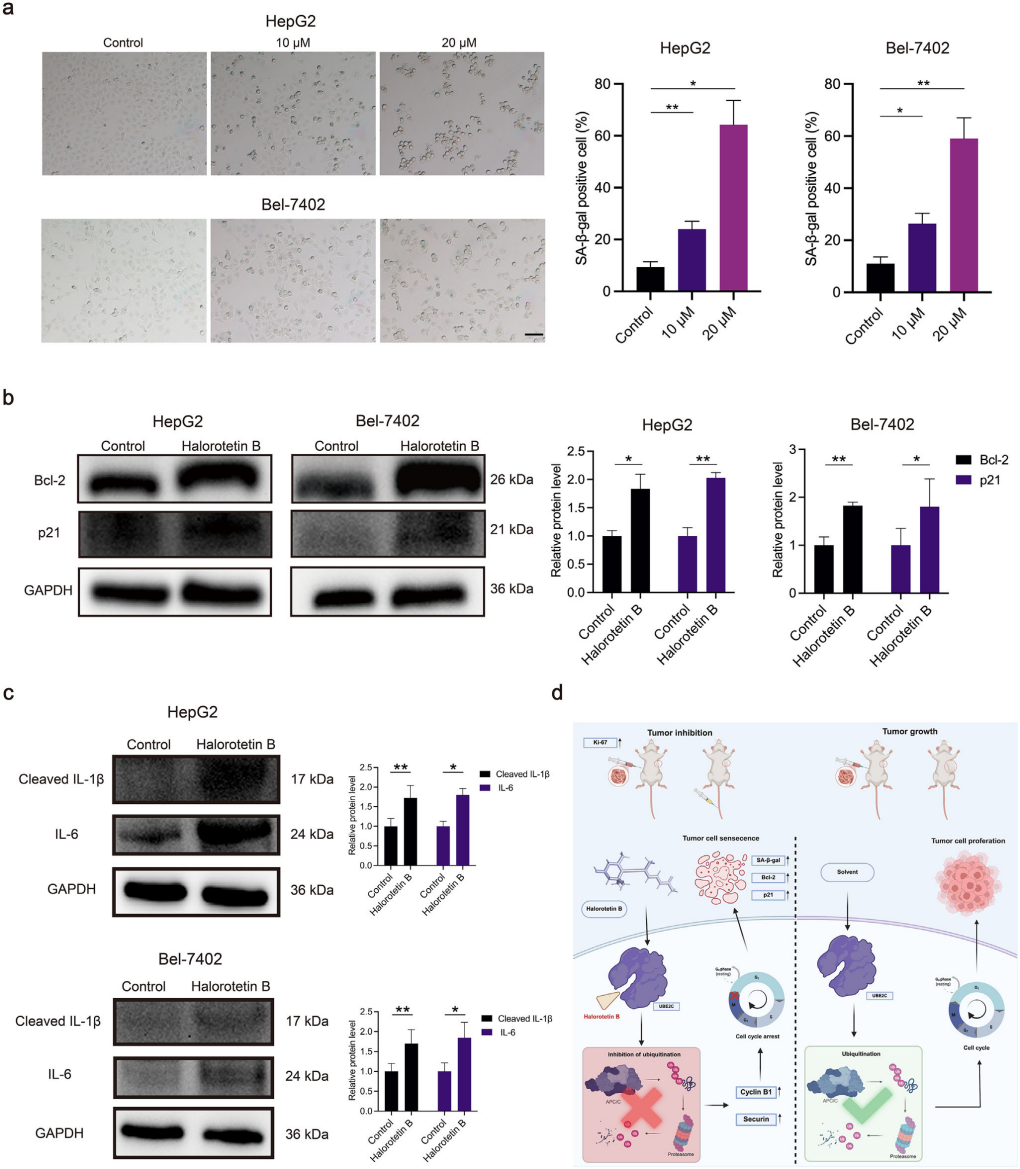
Halorotetin B induces senescence of tumor cells. a) 𝛽‐galactosidase staining and quantification reveals that treated with Halorotetin B for 24 h induced cell senescence of HCC cells, scale bar = 100 µm. Data are presented as mean ± SD. Significance was determined by one‐way ANOVA, ^*^ *p* <0.05, ^**^ *p* <0.01, n = 3 biologically independent samples. b) Western blot analyzed the cell senescence associated proteins level treated with Halorotetin B (20 µm) for 24 h. Data are presented as mean ± SD. Significance was determined by two‐tailed *t*‐test, ^*^ *p* <0.05, ^**^ *p* <0.01, n = 3 biologically independent samples. c) Western blot analyzed the senescence‐associated secretory phenotype (SASP) level treated with Halorotetin B (20 µm) for 24 h. Data are presented as mean ± SD. Significance was determined by two‐tailed *t*‐test, ^*^ *p* <0.05, ^**^ *p* <0.01, n= 3 biologically independent samples. d) Schematic representation depicts the anti‐tumor mechanisms of Halorotetin B.

Inducing cell senescence has been shown to play a crucial role in preventing cancer progression. Several cell cycle inhibitors that target mitotic processes are currently being evaluated as potential inducers of senescence in clinical trials.^[^ ^24^ ^]^ As a cell cycle inhibitor, we investigated whether Halorotetin B induced tumor cell senescence through inducing cell cycle arrest. By assessing senescence‐associated 𝛽‐galactosidase (SA‐𝛽‐gal) staining, a widely used marker for cell senescence, we found that the number of SA‐𝛽‐gal positive cells was significantly increased after Halorotetin B treatment (**Figure** **8**a; Figure S9a, Supporting Information). In addition, western blot assays revealed that the expression of senescence‐associated proteins was also influenced by Halorotetin B (Figure 8b; Figure S9b, Supporting Information). Moreover, the senescence‐associated secretory phenotype (SASP) is a major characteristic of cell senescence. To investigate the effect of Halorotetin B on this process, we analyzed the expression of SASP components, including IL‐1𝛽 and IL‐6 via western blot. The results revealed that Halorotetin B treatment elevated SASP levels (Figure 8c; Figure S9c, Supporting Information). Thus, these data suggest a previously uncharacterized mechanism for inducing senescence of tumor cells via targeting UBE2C by Halorotetin B.

## 3 Discussion

In this study, we identified a novel terpenoid, Halorotetin B, from *H. roretzi*, and demonstrated its potent anti‐tumor efficacy both in vitro and in vivo. Mechanically, Halorotetin B directly binds to UBE2C, inhibiting its activity and thereby reducing the ubiquitin‑mediated degradation of cyclin B1 and securin. This impairment of mitotic exit induces M‑phase cell cycle arrest and promotes tumor cell senescence (Figure 8d). Thus, Halorotetin B represents a novel cell cycle inhibitor that exerts its anti‑tumor effects by targeting UBE2C.

Halorotetin B is a yellow‑colored, oily terpenoid compound. Terpenoid compounds have shown outstanding effectiveness in the development of anti‐cancer drugs. Plant‐derived terpenoid compounds combination with conventional therapeutics play an important role in the treatment of colorectal cancer.^[^ ^25^ ^]^ Similar to the anti‑tumor alkaloid ET‑743 from *E. turbinata*, which functions in chemical defense.^[^ ^26^ ^]^ Halorotetin B production may also contribute to *H. roretzi*’s adaptation to the ocean environment. Structurally, its hydroxyl and carbonyl groups likely mediate binding to biological targets, while the stereochemistry at C‑9 and C‑10 appears to influence both chemical stability and cytotoxic potency. In support of this, we previously characterized Halorotetin A, which differs only in the C‑9/C‑10 configuration and found it to be markedly less effective against tumor cells.^[^ ^27^ ^]^ Based on a comparative analysis with analogous compound, we assign an *R*‑configuration to the C‑3′ hydroxyl group in Halorotetin B.^[^ ^28^, ^29^ ^]^ Functionally, Halorotetin B preferentially inhibits tumor cell proliferation over normal cells, consistent with the elevated expression of *UBE2C* in tumor tissues.^[^ ^30^ ^]^ In nude mouse xenograft studies, Halorotetin B (20 mg kg^−1^) exhibited a superior safety profile, as doxorubicin caused mortality in mice after one time administration at a dose of 20 mg kg^−1^. A major limitation remains its extremely low natural yield: only 0.02 mg of Halorotetin B can be obtained from 1 kg of *H. roretzi* (≈ 0.000002% yield). Therefore, developing efficient chemical synthesis routes or microbial fermentation processes will be critical for advancing Halorotetin B as a lead compound for cancer therapy. We are currently investigating the microbial origin of Halorotetin B to enable its large‐scale production through microbial fermentation process.

The discovery of new therapeutic targets is crucial for the development of promising anti‐tumor drugs and cancer therapies.^[^ ^31^ ^]^ Recent studies have demonstrated that targeting ubiquitin‐like protein conjugation and the ubiquitin‐proteasome system presents a potential strategy for developing anti‐tumor agents.^[^ ^32^ ^]^ Several inhibitors targeting ubiquitin‐conjugating enzymes have been reported. For example, Manadosterol A, isolated from the marine sponge *Lissodendryx fibrosa*, inhibits the Ubc13‐Uev1A interaction,^[^ ^33^ ^]^ while CC0651 is an allosteric inhibitor of the human Cdc34 ubiquitin‐conjugating enzyme.^[^ ^34^ ^]^ Additionally, inhibitors targeting UBE2T and UbcH5c have been described.^[^ ^35^, ^36^ ^]^ UBE2C, a ubiquitin‐conjugating enzyme, is overexpressed in various human cancers, including gastric and ovarian cancers.^[^ ^37^, ^38^ ^]^ In lung adenocarcinoma, the elevated *UBE2C* levels correlate with poor clinical outcomes,^[^ ^39^ ^]^ and its overexpression promotes lymphatic metastasis in bladder cancer.^[^ ^40^ ^]^ Furthermore, UBE2C knockdown inhibits proliferation, migration, and invasion in thyroid carcinoma cells.^[^ ^41^ ^]^ These findings underscore the oncogenic role of *UBE2C* in cancer progression. In our study, we found that *UBE2C* is involved in the proliferation of HCC cells. Moreover, depletion of UBE2C has been shown to reverse cisplatin resistance in ovarian cancer.^[^ ^38^ ^]^ Similarly, *UBE2C* overexpression reduces the sensitivity of HCC cells to sorafenib treatment.^[^ ^42^ ^]^ Therefore, combining Halorotetin B with chemotherapeutics may enhance the efficacy of Halorotetin B for cancer therapy. Given these results, targeting UBE2C could be a promising strategy to control tumor progression. To our knowledge, there have been no canonical, direct, and potent small‐molecule UBE2C inhibitor. Based on our results, Halorotetin B is a novel inhibitor of the UBE2C protein.

Progression through the cell cycle relies on the tightly regulated expression and activity of cyclins and cyclin‐dependent kinases (CDKs). Despite decades of effort, only a limited number of cell cycle inhibitors have successfully reached clinical use, most of which target CDKs or interfere with DNA synthesis.^[^ ^43^ ^]^ Recent studies have identified UBE2C as an oncogenic protein that promotes tumor progression by mediating the ubiquitin‐dependent degradation of cyclin B1 and securin.^[^ ^21^ ^]^ This degradation process is essential for mitotic exit.^[^ ^22^ ^]^ In our study, Halorotetin B was shown to inhibit the ubiquitination and subsequent degradation of cyclin B1 and securin, resulting in the marked cell cycle arrest. These findings position Halorotetin B as a novel cell cycle inhibitor that disrupts cyclin regulation through targeting UBE2C. Furthermore, cellular senescence—an increasingly recognized outcome of cell cycle inhibition—was significantly induced following Halorotetin B treatment, as confirmed by our experimental data.

Due to the effect of Halorotetin B on the cell cycle signaling pathway, only one direct cell cycle‐associated target of Halorotetin B was identified in this study. However, it is well‐established that natural products often exert their biological effects through multiple targets.^[^ ^44^ ^]^ To explore additional anti‐tumor effects of Halorotetin B, we tested whether Halorotetin B exerts anti‐tumor effects via alternative signaling pathways. The anti‐tumor efficacy of Halorotetin B was significantly attenuated by Z‐VAD, an apoptosis inhibitor (Figure S9c, Supporting Information). Furthermore, the migration and invasion of HepG2 cells were also significantly weakened following Halorotetin B treatment (Figure S9d,e, Supporting Information). These results indicated that the involvement of other molecular targets contributes to Halorotetin B's anti‐tumor activity. Further investigation will be necessary to fully elucidate these underlying mechanisms.

In conclusion, our study identifies Halorotetin B as a promising novel therapeutic agent with a defined molecular target. By directly targeting UBE2C, Halorotetin B effectively suppresses tumor growth both in vitro and in vivo without causing significant adverse effects. As a novel natural inhibitor of UBE2C, Halorotetin B represents a valuable lead compound for the development of new anti‐tumor drugs.

## 4 Experimental Section

### Cell Lines, Antibodies, and Chemicals

Cell lines HepG2, Bel‐7402, A549, HGC‐27, Sk‐Hep‐1, Huh‐7, and L929 were purchased from Cell Bank of Shanghai Institute of Cell Biology. Cell lines Mcf‐7, BHT‐101, 95‐D, HeLa, BRL, GES‐1, MRC‐5, and HEK‐293T were purchased from Precella Wuhan. The anti‐Ki‐67 (Cat. No. Ab15580, dilution ratio: 1/30), anti‐UBE2C (Cat. No. EPR‐23165‐31, dilution ratio: 1/1000), anti‐Cyclin B1 (Cat. No. Ab32053, dilution ration for IHC: 1/250, dilution ration for western blot: 1/3000), and anti‐Securin (Cat. No. Ab79546, dilution ration for IHC: 1/250, dilution ration for western blot: 1/5000) antibodies were purchased from Abcam. The anti‐Cyclin A2 (Cat. No. R24022, dilution ration: 1/1000), anti‐Cyclin D1 (Cat. No. R380999, dilution ration: 1/1000), anti‐Cyclin E2 (Cat. No. R24029, dilution ration: 1/1000), anti‐Bcl‐2 (Cat. No. 381702, dilution ration: 1/1000), anti‐IL‐1𝛽 (Cat. No. 516288, dilution ration: 1/1000), anti‐IL‐6 (Cat. No. 347023, dilution ration: 1/1000), and anti‐Ub (Cat. No. 381080, dilution ration: 1/1000) antibodies were purchased from Zenbio. The anti‐p21 (Cat. No. A5163, dilution ration: 1/1000) antibody was purchased from Selleck. The anti‐GAPDH (Cat. No. HC‐301‐01, dilution ration: 1/3000) antibody was purchased from TransGen. The MG‐132 (Cat. No. T2154) and CHX (Cat. No. T1225) were purchased from TargetMol. The Fer‐1 (Cat. No. SML0583), Nec‐1 (Cat. No. 480066), and Z‐VAD (Cat. No. V116) were purchased from Sigma–Aldrich. The doxorubicin (Cat. No. HY‐15142A) were purchased from MedChemExpress (MCE).

### Natural Products Extraction

The *Halocynthia roretzi* were collected from Rongcheng city (Shandong province). Then the *H. roretzi* were dissected and the tunic were soaked in ethyl acetate for one week. After a week, the supernatant was collected as the natural products extract. All organic reagents were purchased from Sinopharm Chemical Reagent Co., Ltd. (SCR, Shanghai, China).

### Silica Gel Chromatography

The silica gel column was assembled with 300–400 mesh silica gel. Next, the tunic ethyl acetate extract was added into 200–300 mesh silica gel and then added into the silica gel column. The top of the column was filled with 60–80 silica gel. Then the whole column was wetted by petroleum ether. The elution mobile phase was configured according to different proportions of ethyl acetate and petroleum ether. Finally, methanol eluted all reaming components.

### Preparative Layer Chromatography (PLC)

The silica gel plates were purchased from Merck (1.13895.0001). After silica gel chromatography, the components with anti‐tumor efficacy were separated by PLC. Adding the components to be separated on the silica gel chromatography. The dichloromethane and methanol (15:1) as elution mobile phase.

### High Performance Liquid Chromatography (HPLC)

The HPLC instrument was purchased from HITACHI (L‐2000), and the C18 reverse column was purchased from Kromasil (M05CLA25). The components with anti‐tumor efficacy after PLC separation were further separated by HPLC. The methanol and pure water as elution mobile phase, using gradient elution, the proportion of methanol increased linearly from 20% to 100% within 50 min.

### NMR Spectrum and Mass Spectrum

The compound was dissolved in Methanol‐*d*_4._ NMR spectra was recorded by a JEOL JEM‐ECP NMR spectrometer (600 MHz for ^1^H NMR and 150 MHz for ^13^C NMR). HRESIMS was tested on a Thermo MAT95XP high‐resolution mass spectrometer. HRESIMS [M+Na]^+^ m/z 295.1666, (calcd for C_18_H_24_O_2_Na, 295.1669).

### Cell Viability Detection

The tumor cells were seeded on the 96‐well plate (Thermo Fisher, Cat# 267427). Then the cells were cultured in the incubator for 24 h. When the cell density reaches ≈60%, the tested compound was added to the medium. Next, the cells were continued to be culture for 48 h. Then the CCK‐8 (Dojindo) was added to the medium and incubated for 90 min. Finally, the absorbance values of each group at 450 nm were detected, and then obtained cell viability data.

### Colony Formation

The tumor cells were seeded on the 6‐well plate (Thermo Fisher, Cat# 150239). The number of cells in each well was ≈700. Then the cells were cultured in the incubator for 24 h. Next, the tested compound was added to the medium. After that, the cells were continued to be culture for one week, during this time, the culture medium was changed every day. After cultivation, the cells were fixed with 4% paraformaldehyde (Sigma–Aldrich, CAS# 30525‐89‐4) for 30 min. Then the cells were dyed with crystal violet (Sigma–Aldrich, CAS# 548‐62‐9) for 15 min. After dyeing, cells were cleaned three times by PBS (Thermo Fisher, Cat# 10010023), and the number of clones was imaged and recorded.

### Cell Culture

All cell lines were cultured in a humidified incubator at 37 °C with 5% carbon dioxide, with DMEM medium (BasalMedia) including streptomycin, penicillin, and 10% fetal bovine serum (FBS, Gibco). Cell lines HepG2, Bel‐7402, A549, HGC‐27, Sk‐Hep‐1, Huh‐7, and L929 were purchased from Cell Bank of Shanghai Institute of Cell Biology. Cell lines Mcf‐7, BHT‐101, 95‐D, HeLa, BRL, GES‐1, MRC‐5, and HEK‐293T were purchased from Precella Wuhan. All cell lines were tested for mycoplasma before experiment and had passage numbers of ≤10.

### In Vivo Experiments

The Balb/c Nude mice were purchased from Charles River. HepG2 cells were cultured in the incubator. Then the HepG2 cells were subcutaneous injected in the nude mice, and the amounts of cells inoculated per mouse was 5 × 10^6^ cells. Next, the inoculated mice were placed in the IVC animal room and were randomly divided into seven groups when the tumor volume reached to 100 mm^3^. The tumor volume was calculated as V = (length × width^2^)/2. Then treated nude mice with different reagents. The solvent for the control group, Halorotetin B group, and positive control group was 95% saline, 3% Tween‐80, and 2% DMSO. The tumor volume and body weight were detected every three days and tumor measurements were performed by an investigator who was blinded to the group allocations. The reagents were treated for four times per week (Monday, Wednesday, Friday, and Sunday). After the administration, the tumor and organs were collected, photographed. Next, the tumor and organ tissue were collected for IHC staining and H&E staining. Except for the mice that died in the positive control group (Doxorubicin, 20 mg kg^−1^), other mice were not excluded from the data statistics. All animal studies and handling were performed in accordance with Scientific Ethics Review Board of Qingdao Marine Biomedical Research Institute (E‐MBHB‐2025‐2‐20).

### Tumor Spheroid Experiments

The tumor cells were seeded on the 96‐well plate, and the cell density adjusted to 1.5×10^5^ cells / mL. The plate was then placed in a centrifuge for centrifugation. Next, the plate was placed into incubator. The medium was changed every two days and observed the morphology of tumor spheroids. When the tumor spheroids were formed, treated them with Halorotetin B.

### RNA Extraction and RNA‐Seq

HepG2 cells for RNA extraction were centrifuged in 1.5 mL RNase‐free centrifuge tubes for several seconds using a low‐speed centrifuge to remove the supernatant. RNAsio Plus (Takara, Cat# 9108Q) was added to the centrifuge tube and then the cells were fully lysed by vortexing and shaking. Then chloroform was added to the centrifuge tube, vortexed, and shaken until the liquid turned pink. Then the centrifuge tubes were put on ice for 10 min. Subsequently, the supernatant was taken into a new 1.5 mL RNase‐free centrifuge tube after centrifugation at 12 000 rpm for 15 min at 4 °C using a refrigerated high‐speed centrifuge. Then the isopropanol was added. Centrifuge tubes were centrifuged at 12 000 rpm for 15 min at 4 °C using a refrigerated high‐speed centrifuge after leaving for 15 min at room temperature. The supernatant was removed, and 75% ethanol (RNase‐free) was added to the precipitate. Centrifuge tubes were centrifuged at 12 000 rpm for 15 min at 4 °C using a refrigerated high‐speed centrifuge. The supernatant was removed, the residual ethanol was volatilized entirely at room temperature, and DEPC‐treated water (Biological Industries, Cat# 7732‐18‐5) was added to the centrifuge tube to obtain the RNA of HepG2 cells. Finally, the RNA was sent to company for RNA‐Seq (BIOMARKER TECHNOLOGIES, Beijing, China). Clean reads obtained from BIOMARKER were aligned to the genome GRCh38.p14 (accession: GCF_000001405.40) from NCBI using Bowtie2 (2.4.4). Alignment files were converted to BAM format and sorted with Samtools (1.7). Gene quantification was then performed using the FeatureCounts (2.0.6). The obtained count files were subjected to differential expression analysis using DESeq2 (1.10.1). Genes with a p‐adjust value lower than 0.05 and log2FoldChange bigger than 1 or lower than −1 were defined as differential expressed genes (DEG).

### KEGG Analysis

Kyoto Encyclopedia of Genes and Genomes analysis was performed using an online platform (https://www.bioinformatics.com.cn). The RNA‐seq data to this tool and obtained the associated pathways were submitted.

### Cell Cycle Analysis

Tumor cells were seeded on the 6‐well plate. Then treated them with tested compound and incubated with the compound for 24 h. After that, tumor cells were fixed with 70% ethanol and stained with propidium iodide/RNase (Beyotime, Cat# C1052) staining solution at incubator for 30 min. The cell cycle was analyzed by flow cytometry (BECKMAN COULTER, CytoFLEX, California, USA).

### Target Identification (PELSA)

HepG2 cells were cultured and harvested for total protein extraction. The protein concentration was determined using the BCA assay. For each sample, 50 µg of protein was aliquoted for the experiment. The protein solution was treated with a stock solution of the drug to a final concentration of 20 µm, while the control group received an equal volume of the blank solvent. The mixture was incubated at room temperature for 20 min with shaking at 600 rpm. Subsequently, 5 µL of trypsin (at a concentration of 5 µg µL^−1^) was added and immediately mixed thoroughly. Digestion was carried out at 37 °C for 1 min with shaking at 1000 rpm. The reaction was terminated by boiling the samples for 10 min. Guanidine hydrochloride was then added to achieve a final concentration of 6 m. Following reduction and alkylation, the samples were processed using a 10 kDa molecular weight cut‐off ultrafiltration tube. They were centrifuged at 14 000 × g to collect the flow‐through. Trifluoroacetic acid (TFA) was added to the filtrate to a final concentration of 1%, followed by centrifugation at 16 000 × g for 10 min. The resulting supernatant, containing the peptides, was desalted using a C18 Cartridge. The peptide concentration was measured, and the samples were vacuum‐dried. An appropriate amount of peptide from each sample was subjected to chromatographic separation using a Vanquish Neo UHPLC system (Thermo Scientific). The separated peptides were then analyzed by data‐independent acquisition (DIA) mass spectrometry on an Orbitrap Astral mass spectrometer (Thermo Scientific). Proteins corresponding to peptides with a *p*‐value of less than 0.05 and a fold change (FC) of less than 1 were identified as potential candidate target proteins.

### Target Identification (OTTER)

The RNA‐Seq data and the keyword were submitted to the OTTER (http://otter‐simm.com/otter.html). The workflow of OTTER consists of three steps. First step, for each differentially expressed genes, OTTER scans the keyword in all PubMed abstracts of this gene. Then a text score was calculated for this gene. Second step, OTTER takes into account the protein‐protein interactions (PPI) among these differentially expressed genes, then the PPI score was calculated. Third step, final scores are calculated using the sum of text scores and PPI scores. After a few days, the most possible targets were got of Halorotetin B and verified them by using CETSA.

### Cellular Thermal Shift Assay (CETSA)

Tumor cells were seeded on the 6‐well plate. Then treated them with the tested compound. After incubated for 24 h, cells were collected for protein extraction. The protein supernatant was divided into seven groups. Each group was heated using PCR apparatus (Bio‐Rad, T100 Thermal Cycler, California, USA). Then the degradation of protein was detected by Western blot.

### Surface Plasmon Resonance (SPR)

A solvent correction was performed using running buffer matching the analyte. Each analyte was diluted to a series of concentrations in a 96‐well plate. The samples were injected over the target protein‐immobilized chip surface sequentially from the lowest to the highest concentration. The flow rate was maintained at 30 µL min^−1^ with an association/dissociation contact time of 360 s. After the passage of each concentration, the sensor chip was regenerated for 5 min using a 10 mm glycine‐HCl solution (pH *2.0*). This cycle was repeated until all concentrations for every analyte had been analyzed. The resulting sensorgrams were globally fitted to a 1:1 Langmuir binding model using the Biacore Insight Evaluation Software (Cytiva, Marlborough, MA, USA) to determine the association and dissociation rate constants.

### Microscale Thermophoresis (MST)

The MST measurements for binding of Halorotetin B to UBE2C were performed as described previously. Briefly, recombinant human UBE2C protein (AntibodySystem, Cat# YHA19001) labeling the protein RED‐tris‐NTA dye was mixed with Halorotetin B at different concentrations. Then, all samples were incubated for 1 h and analyzed by MST device (NanoTemper, Monolith NT.115, Munich, Germany).

### Enzymatic Activity Detection

By using the ubiquitylation assay kit (Abcam, Cat. No. ab139467). The activity of wide‐type UBE2C protein or Halorotetin B‐binding UBE2C protein was detected through E2‐Ub complex formation. Assay reagents were combined and incubated at 37 °C for 4 h. Next, assays with non‐reducing gel loading buffer were quenched and performed Western blot experiment.

### Purification of Wild‐Type and Ser51‐Mutant UBE2C Proteins

First, a plasmid with a GFP tag was transfected into HEK‐293T cells, which were then cultured at 37 °C for 24 h. After 24 h, protein expression was confirmed using fluorescence microscopy. Following successful expression, the HEK‐293T cells treated with Halorotetin B for 24 h. Then the cells were lysed to extract the total protein. The protein lysate was then incubated with GFP‐tagged magnetic beads (Engibody, Cat. No. AT1773) at 4 °C overnight. After incubation, the beads were washed thoroughly to remove non‐specifically bound proteins. Then loading buffer was added to the beads, and the samples were boiled at 100 °C for 10 min. Finally, the purified protein was analyzed by SDS‐PAGE, followed by Coomassie Brilliant Blue staining to assess its purity.

### Molecular Docking

The crystal structure of UBE2C (PDB id: 4YII) were downloaded from RCSB Protein Data Bank (PDB, https://www.rcsb.org/). Then removed the ligand (APC2) of UBE2C protein by Pymol software (Pymol 2.5.4, https://www.pymol.org/). The water molecules from UBE2C protein were also removed. Next, the AutoDockTools software was used for molecular docking of UBE2C protein and Halorotetin B. The model with the least docking energy was selected to exhibit the docking site. Of the top ten models with the lowest docking energy, only six produced docking sites. Finally, the mutation plasmids were constructed according to these sites.

### Plasmid Construction and Transfection

The coding sequences of human UBE2C were amplified and cloned into the pcDNA3.1‐eGFP vector. Then, according to the docking site information, The mutation plasmids on the pcDNA3.1‐eGFP‐UBE2C vector were constructed. Subsequently, the plasmids were sequenced to make sure that each site mutated successfully. Next, the plasmids into HEK‐293T cells were transfected by Lipofectamine 3000 (Thermo Fisher, Cat# L300008), and performed cytotoxicity detection of Halorotetin B and protein extraction.

### Gene Silencing (RNAi)

The siRNAs for UBE2C were purchased from GenePharma (Shanghai, China), and the sequences were as follows:

1. Sense‐CCUUCCCUGAAUCAGACAATT, Antisense‐UUGUCUGAUUCAGGGAAGGTT.
2. Sense‐GGUAUAAGCUCUCGCUAGATT, Antisense‐UCUAGCGAGAGCUUAUACCTT.
3. Sense‐AUUGAUAGUCCCUUGAACATT, Antisense‐UGUUCAAGGGACUAUCAAUTT.
4. Sense‐CUAGCGUCGCCGCCGCCCGTT, Antisense‐CGGGCGGCGGCGACGCUAGTT.

The negative control was used as a control (Sequences: Sense‐UUCUCCGAACGUGUCACGUTT, Antisense‐ACGUGACACGUUCGGAGAATT). Cells were seeded at 6‐well plate and cultured for 24 h. Cells were transfected with four siRNA duplexes by Lipofectamine 3000 and incubated for 48 h.

### Quantitative Real‐Time PCR

Total mRNA was reverse transcribed into cDNA using the R223‐01 HiScript II Q RT SuperMix Kit (Vazyme, Cat# R223‐01). Quantitative RT‐PCR was performed using LightCycler 96 Instrument (Roche, Basel, Switzerland), and the sequences of the primers were as follows:

Cyclin B1: Sense‐AATACCTGATGGAACTAACTAT, Antisense‐GCATAACTGGAAGAAGAGA.

Securin: Sense‐GATGACTGAGAAGACTGTTA, Antisense‐GAGGATTGAAGGGAAAGAA.

### Cell Senescence Detection

Tumor cells were seeded at 6‐well plate and cultured for 24 h. Then the cells were treated with Halorotetin B and cultured for 24 h. Cell senescence was detected by a senescence 𝛽‐galactosidase staining kit (Beyotime, Cat# C0602). The cells were incubated with fixative buffer for 15 min and stained with working staining solution at 37 °C for 12 h. The positively stained cells were imaged by microscope.

### Western Blot Analysis

Tumor cells were seeded at a 6‐well plate and cultured for 24 h. Then the cells were treated with tested compound for 24 h. All the cells were collected for Western blot analysis. Total cellular proteins were extracted with Cell lysis buffer for Western and IP (Beyotime, Cat# P0013). Manually adjust the internal parameters to be consistent in each experiment. Next, protein was separated by 10% SDS‐PAGE and transferred onto PVDF membranes (Merck, Cat# IPFL00010). Next, the membranes were blocked in 5% nonfat milk for 1 h and then incubated with specific primary antibodies at 4 °C overnight. Secondary antibodies were added and incubated for 2 h at room temperature. The immunoreactive bands were imaged by an imaging system (Tanon, Shanghai, China).

### Immunohistochemistry (IHC)

Tumor from xenograft mice were harvested. Next, the tumor tissues were fixed with 4% paraformaldehyde. The tumor tissue sections were embedded in paraffin and then deparaffinized in xylene and treated with alcohol and distilled water. After antigen retrieval, endogenous peroxidase was blocked. Subsequently, BSA (Servicebio, Cat# GC305010) was blocked at room temperature for 30 min, and the antibodies (cyclin B1‐1:200, securin‐1:200, Ki‐67‐1:500) were incubated at 4 °C overnight. Next, the secondary antibodies (1:300) were incubated at room temperature for 50 min and DAB (Servicebio, Cat# G1212) were used for detection, and the slides were counterstained with hematoxylin (Servicebio, Cat# G1004). Images were captured with microscope.

### H&E Staining

The vital organs (Heart, Liver, Spleen, Lung, Kidney) from xenograft mice were harvested. Then the organs were fixed with 4% paraformaldehyde. Then the sections were embedded in paraffin and then deparaffinized. Next, the frozen sections were removed from the −20 °C refrigerator and restored to room temperature, fixed with tissue fixating solution (Servicebio, Cat# G1101) for 15 min, and then rinsed with running water. Subsequently, hematoxylin and eosin (Servicebio, Cat# G1076) were stained sequentially. After the staining was completed, the sections were dehydrated and sealed. Finally, Images were captured with microscope and then analyzed.

### Cell Migration

In the beginning, draw a positioning line on the 24‐well plate (Thermo Fisher, Cat# 142475) to ensure that the shooting position of 0 and 48 h was consistent. Then the tumor cells were seeded on the 24‐well plate. After the cells attached to the wall, drawn the lines between cells and photographs were taken to record the size of the scratches. Then the tested compound was added to the medium and incubated with tumor cells for 48 h. The medium was FBS‐free. Next, the scratch area was imaged and counted.

### Cell Invasion

The matrix (Corning Matrigel, Cat# 354248) was spread in the Transwell (Corning, Cat# 354480). The ration of matrix and FBS‐free medium was 1:8. Then incubated for 3 h. Next, removed the excess liquid and added medium to Transwell. 30 min later, removed the liquid, and added the complete medium to the lower chambers. Meanwhile, the Transwell were filled with tumor cells and the tested compound. After being incubated and allowed to migrate for 24 h, non‐migratory cells above the upper chambers were removed with cotton swabs. Then the lower Transwell surfaces were fixed with 4% paraformaldehyde and stained with crystal violet for 20 min. After that, PBS washed the Transwell three times. The cells were imaged and counted using a microscope.

### Schematic Diagram and Mechanism Diagram Drawing

Schematic diagram and mechanism diagram were created in BioRender (https://BioRender.com).

### Statistical Analysis

Statistical differences between groups were analyzed with analysis of student's *t*‐test or variance (ANOVA), as appropriate, using GraphPad Prism 9 software (RRID: SCR_002798). All data represented in plots represent mean ± s.e.m. or mean ± SD. Data distribution was to be normal after testing. All in vitro assays were performed in biological replicates. The IC_50_ fitting curve was drawn as follows: nonlinear regression was performed after logarithmic transformation of the concentration. The formula used for calculating IC_50_: Y = Bottom + (Top × Bottom) / [1 + 10^(Log (IC50) × X)^] × HillSlope, Top was manually set to 1 and The Bottom was manually set to 0, the fitting curve of IC_50_ obtained X and Y. No data were excluded. The xenograft tumor experiments were randomized. The numbers of biologically independent experiments, the number of individual mice and details of statistical tests performed can be found in the respective figure legends or method section. Data analysis and collection were not performed blind to the conditions of the experiments.

## Conflict of Interest

The authors declare no conflict of interest.

## Author Contributions

S.H. performed Conceptualization, Sample collection, Investigation, Formal analysis, Writing – original draft, and Writing – review & editing; J.L. performed Formal analysis, Visualization, and Writing – original draft; Y.Z. performed Extraction and isolation of H.B.; Y.Z. performed Structure elucidation of H.B.; P.L. performed Bioinformatics analysis; M.H. performed RNA extraction and partial PCR assays; B.D. performed Conceptualization, Funding acquisition, Project administration, and Writing – review & editing.

## Supporting information



Supporting Information

Supporting Information

Supporting Information

## Data Availability

RNA sequencing data have been deposited on the NCBI Gene Expression Omnibus under PRJNA1247715. Any additional information required to reanalyze the data reported in this paper is available from the lead contact upon request.
