# Supplementary material for: Halorotetin B, A Novel Terpenoid Compound Derived from Marine Ascidian, Suppresses Tumor Growth by Targeting the Cell Cycle Regulator UBE2C
Source: Adv Sci (Weinh). 2025 Dec 12;13(12):e15652. doi: 10.1002/advs.202515652 (PMC12948284; doi:10.1002/advs.202515652)
Supplement: Supplementary file 1 — Supporting Information [file ADVS-13-e15652-s003.docx]

**Supporting Information**

**Halorotetin B, a novel terpenoid compound derived from marine ascidian, suppresses tumor growth by targeting the cell cycle regulator UBE2C**

Shanhao Han^1^, Jianhui Li^1^, Yuting Zhu^1^, Penghui Liu^1^, Yaoyao Zheng^3^, Muchun He^4^, and Bo Dong^1,2,5,*^

^1^Fang Zongxi Center for Marine EvoDevo, MoE Key Laboratory of Marine Genetics and Breeding, College of Marine Life Sciences, Ocean University of China, Qingdao 266003, China

^2^Laboratory for Marine Biology and Biotechnology, Qingdao National Laboratory for Marine Science and Technology, Qingdao 266237, China

^3^Department of Pharmacy, Affiliated Hospital of Shandong University of Traditional Chinese Medicine, Jinan 250011, China

^4^Liaoning Key Laboratory of Marine Animal Immunology and Disease Control, Dalian Ocean University, Dalian 116023, China

^5^Institute of Evolution & Marine Biodiversity, Ocean University of China, Qingdao 266003, China

*Correspondence: [bodong@ouc.edu.cn](mailto:bodong@ouc.edu.cn)


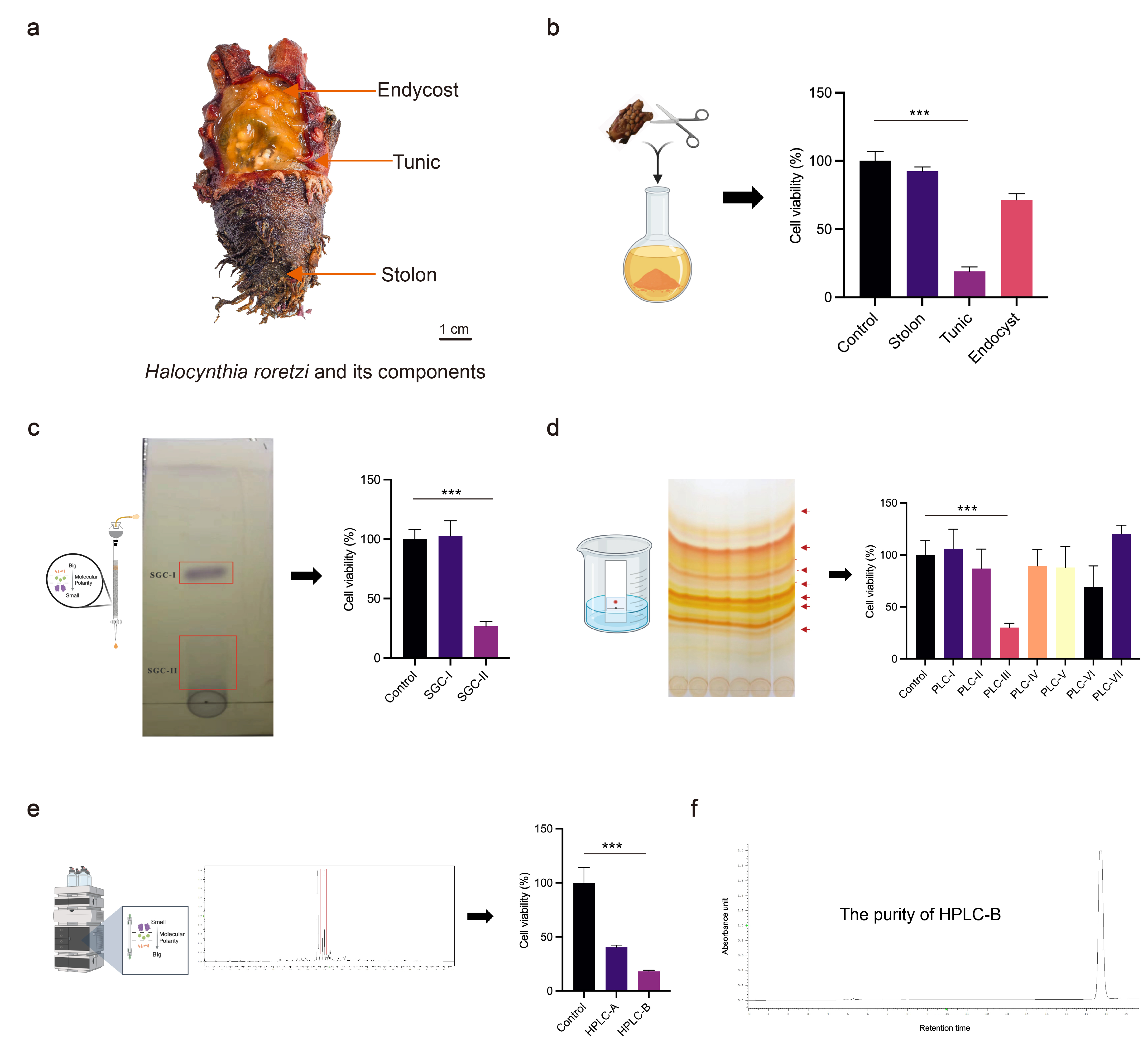


**Figure S1. Discovery of the anti-tumor small-molecule from *Halocynthia roretzi*.**

(**a**) The *H. roretzi* and its three structure parts. The endycost is the edible part, the tunic is composed of cellulose, and the stolon plays attachment role in the ocean environment. Bar = 1 cm. (**b**) The tumor cell cytotoxicity of different parts extraction from *H. roretzi* (HepG2 cells, 200 μL/mL). Data are presented as mean ± SD. Significance was determined by one-way ANOVA, *** *p* < 0.001, n = 3 biologically independent samples. (**c**) The two parts of tunic extraction obtainted by silica gel column chromatography, the SGC-II was the active part (HepG2 cells, 500 μg/mL). Data are presented as mean ± SD. Significance was determined by one-way ANOVA, *** *p* < 0.001, n = 3 biologically independent samples. (**d**) The different parts of SGC-II obtained by PLC separation, and the PLC-III exhibited the strongest tumor cell cytotoxicity (HepG2 cells, 100 μg/mL). Data are presented as mean ± SD. Significance was determined by one-way ANOVA, *** *p* < 0.001, n = 3 biologically independent samples. (**e)** The different parts of PLC-III obtained by semipreparative-HPLC. HPLC-B exhibited higher cytotoxicity to tumor cells (HepG2 cells, 10 μg/mL). Data are presented as mean ± SD. Significance was determined by one-way ANOVA, *** *p* < 0.001, n = 3 biologically independent samples. (**f)** The purity of HPLC-B was detected before structure identification.

a


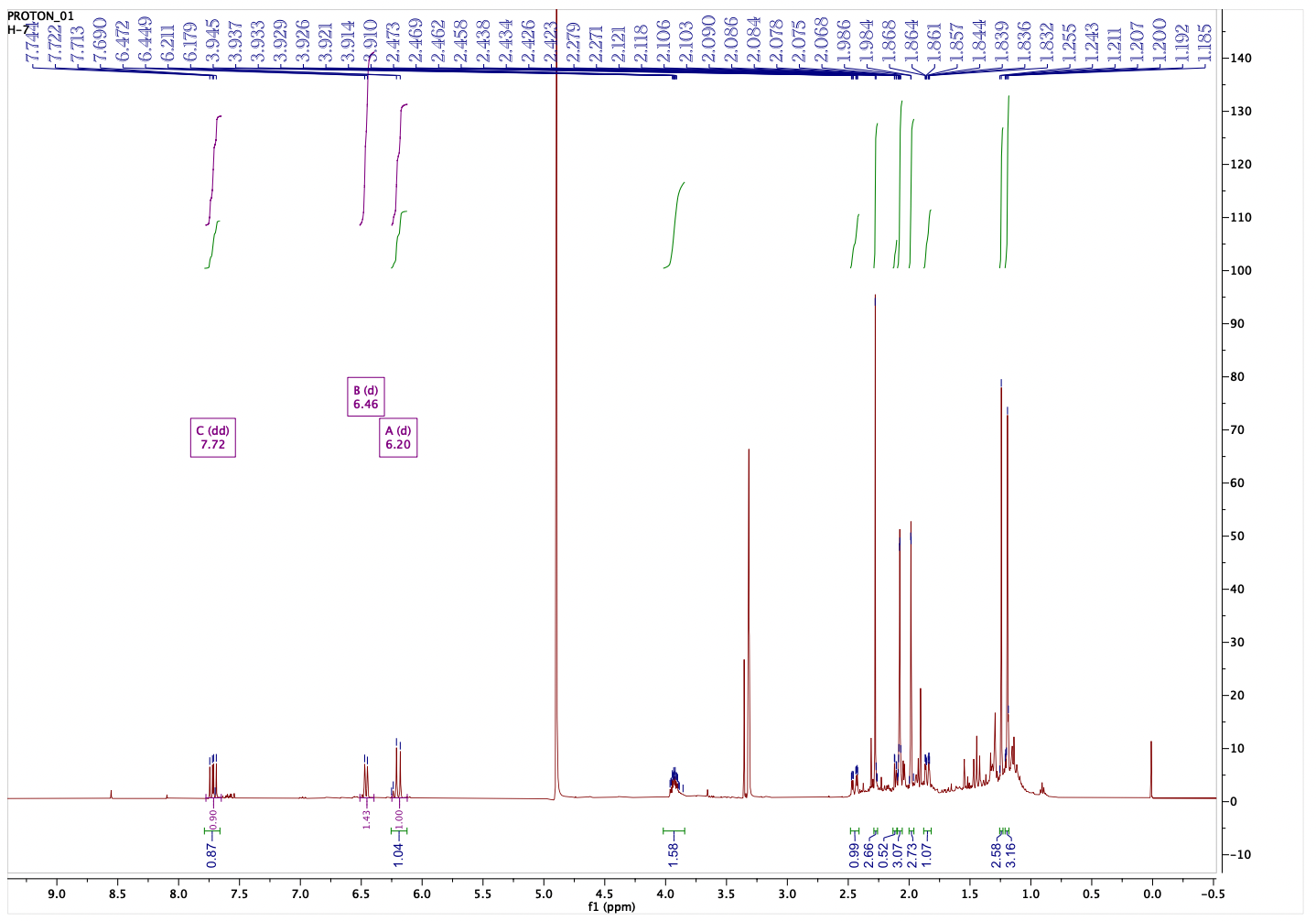


^1^H NMR (600 MHz, Methanol-*d*_4_) δ_H_ 1.43 (1H, t, *J* = 12.1 Hz, H-2a), 1.84 (1H, ddd, *J* = 12.1, 3.6, 2.0 Hz, H-2b), 3.92 (1H , dddd, *J* = 11.8, 9.4, 5.6, 3.5 Hz, H-3), 2.09 (1H, m, H-4a), 2.44 (1H, dd, *J* = 17.9, 5.3 Hz, H-4b), 6.45 (1H, d, *J* = 11.2Hz, H-10), 7.71 (1H, dd, *J* = 15.6, 11.2 Hz, H-11), 6.18 (1H, d, *J* = 15.6 Hz, H-12), 2.27 (3H, s, H-14), 1.18 (3H, s, H-15), 1.23 (3H, s, H-16), 1.97 (3H, s, H-17), 2.07 (3H, s, H-18).

b


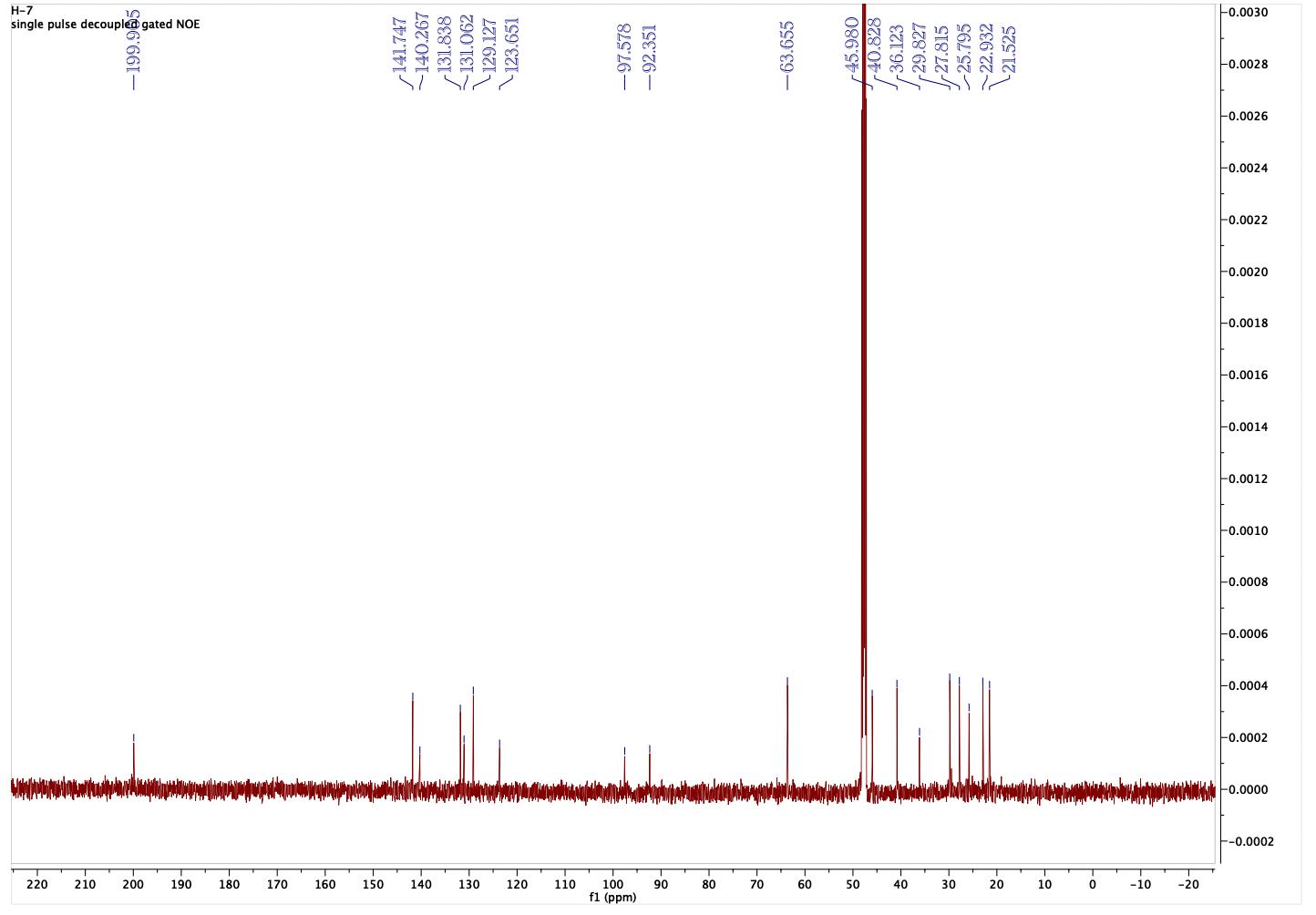


^13^C NMR (150 MHz, Methanol-*d*_4_) δ_C_ 36.1 (C-1), 46.0 (C-2), 63.6 (C-3), 40.8 (C-4), 140.3 (C-5), 123.6 (C-6), 97.6 (C-7), 92.3 (C-8), 131.1 (C-9), 131.8 (C-10), 141.7 (C-11), 129.1 (C-12), 199.9 (C-13), 25.8 (C-14), 27.8 (C-15), 29.8 (C-16), 21.5 (C-17), 22.9 (C-18).

c


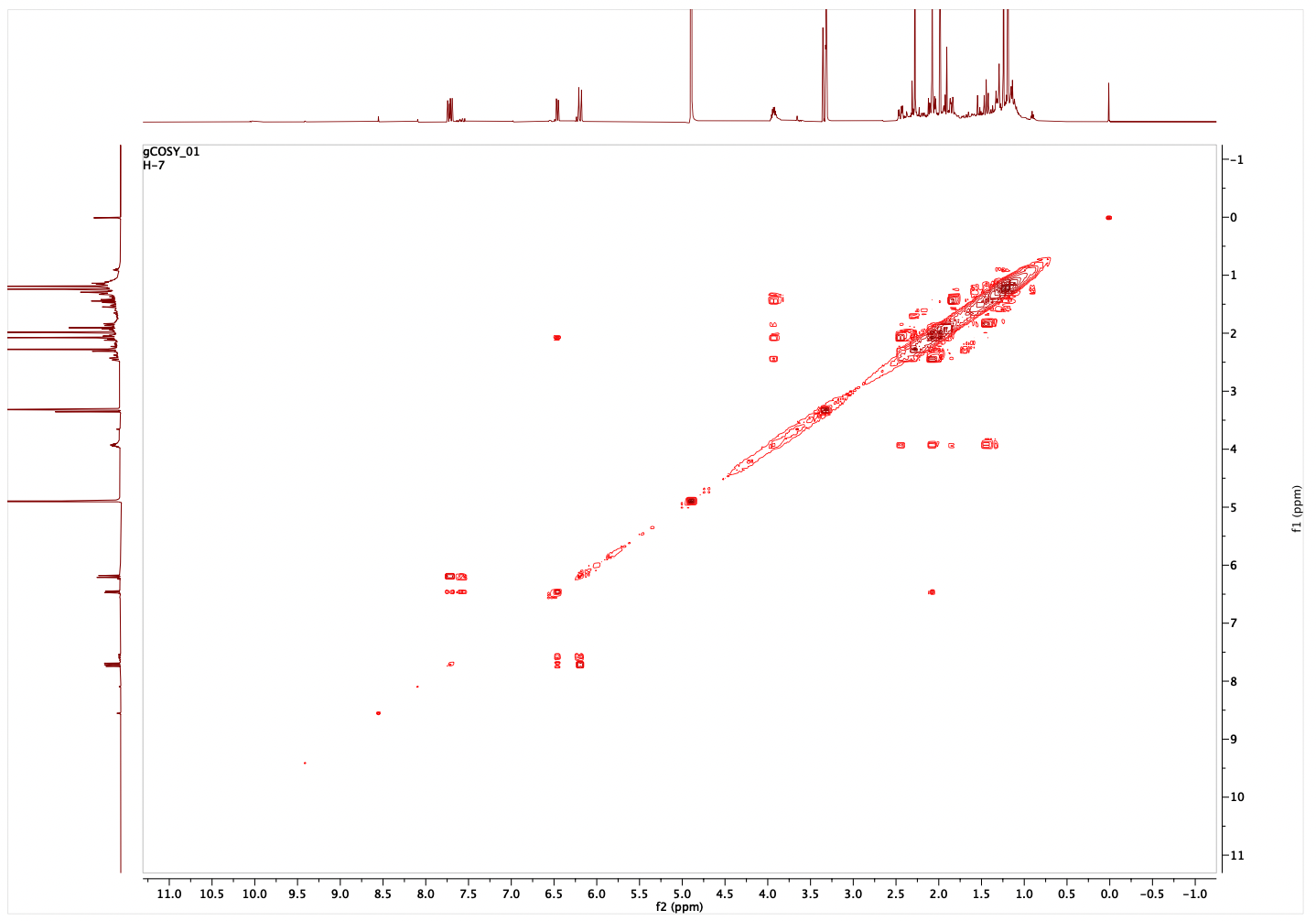


d


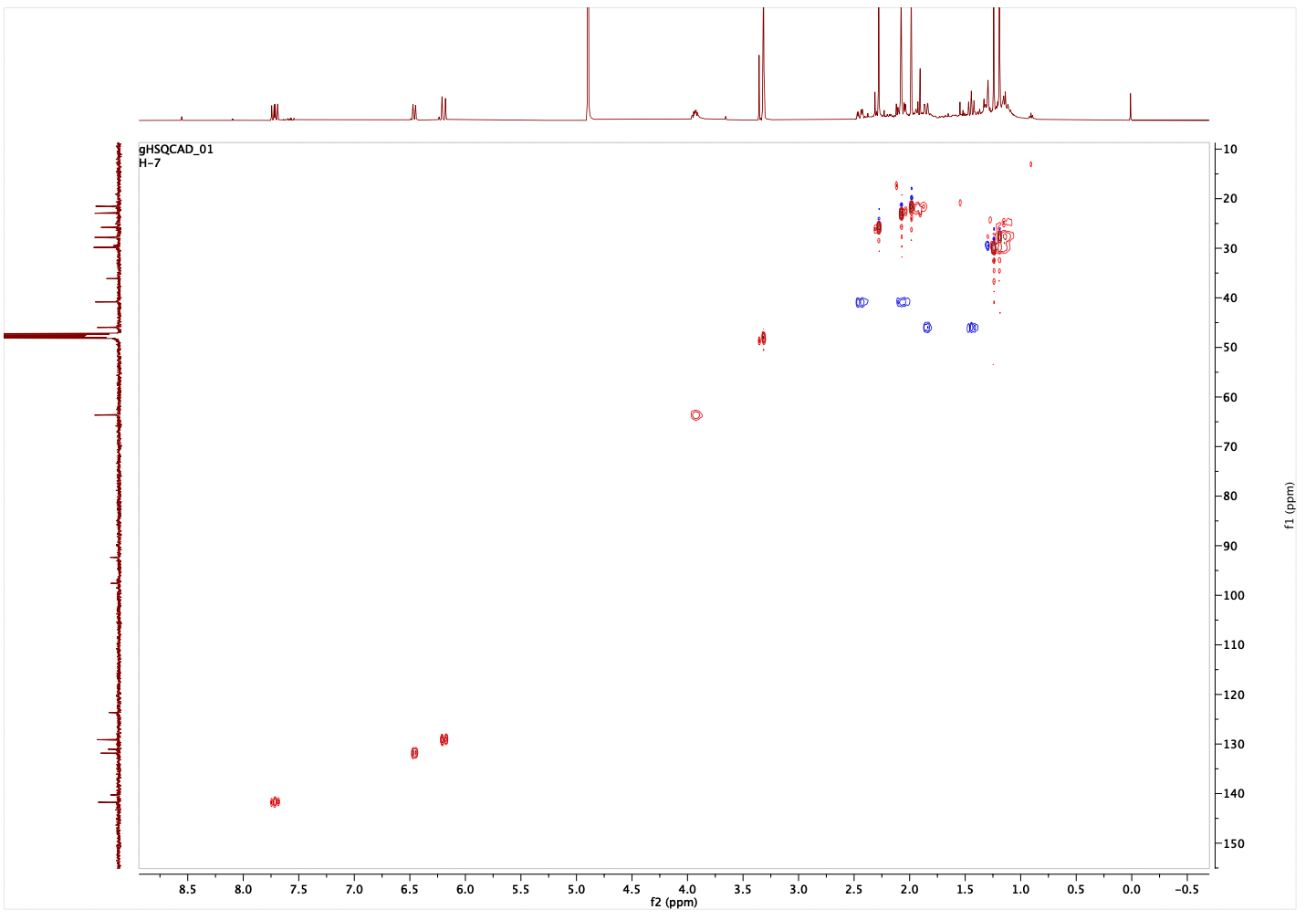


e


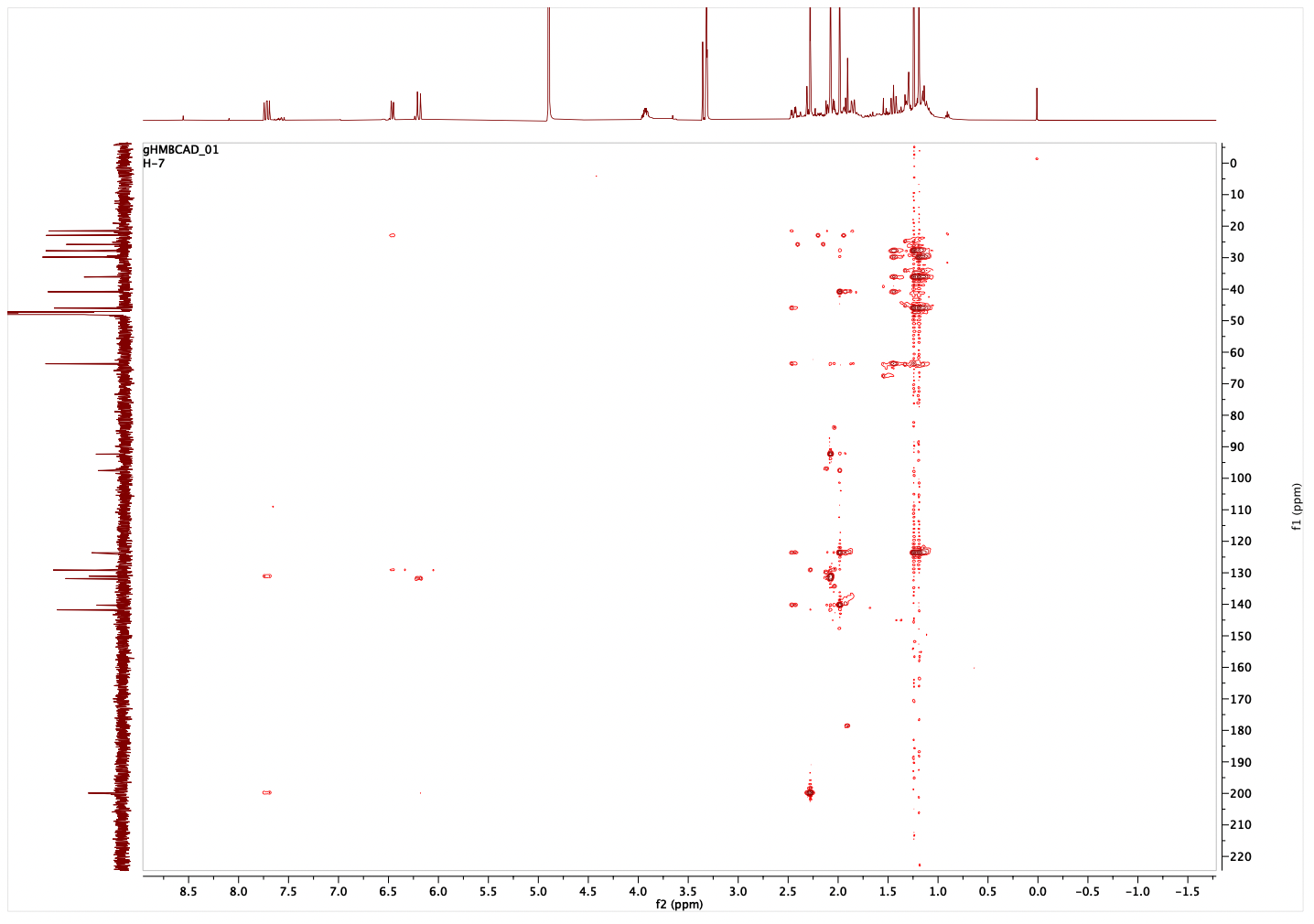


f


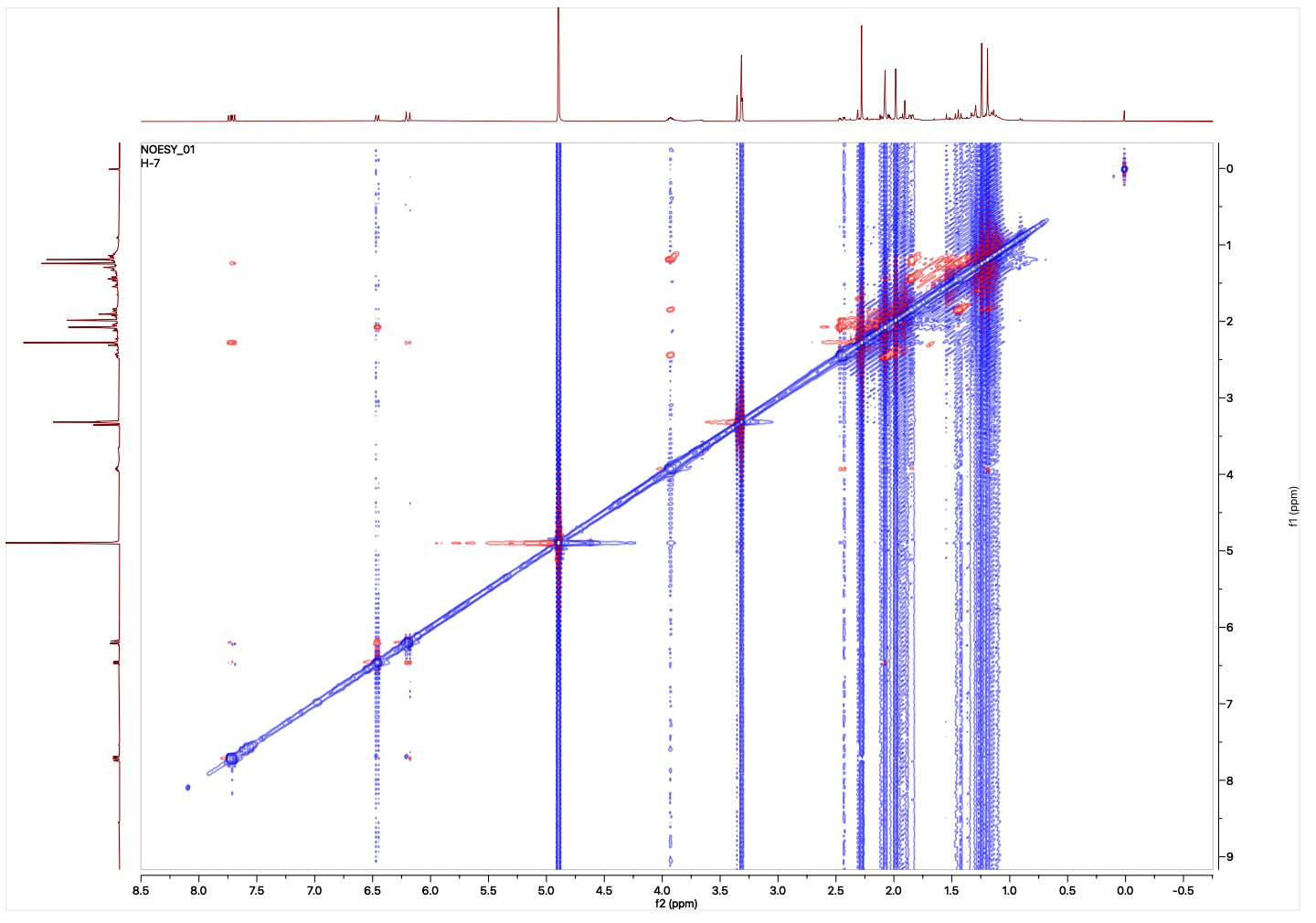


g


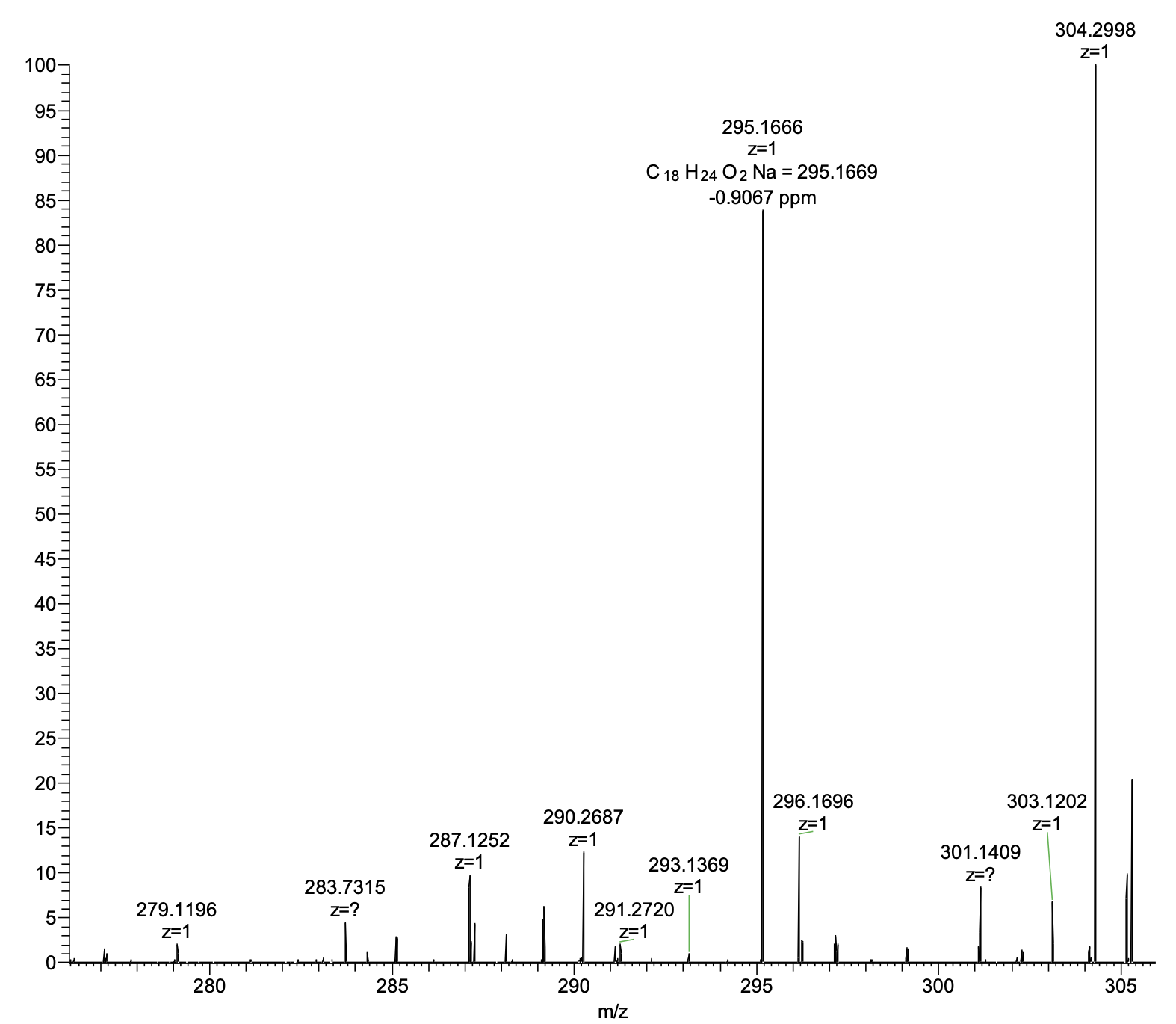


HRESIMS [M + Na]^+^ *m/z* 295.1666 (calcd for C_18_H_24_O_2_Na, 295.1669).

**Figure S2. NMR and HRESIMS spectrum of Halorotetin B.**

(**a**) ^1^H NMR spectrum of Halorotetin B in Methanol-*d*_4_. (**b**) ^13^C NMR spectrum of Halorotetin B in Methanol-*d*_4_. (**c**) ^1^H-^1^H COSY spectrum of Halorotetin B in Methanol-*d*_4_. (**d)** HSQC spectrum of Halorotetin B in Methanol-*d*_4_. (**e)** HMBC spectrum of Halorotetin B in Methanol-*d*_4_. (**f)** NOESY spectrum of Halorotetin B in Methanol-*d*_4_. (**g)** HRESIMS [M+Na]^+^ spectrum of Halorotetin B.


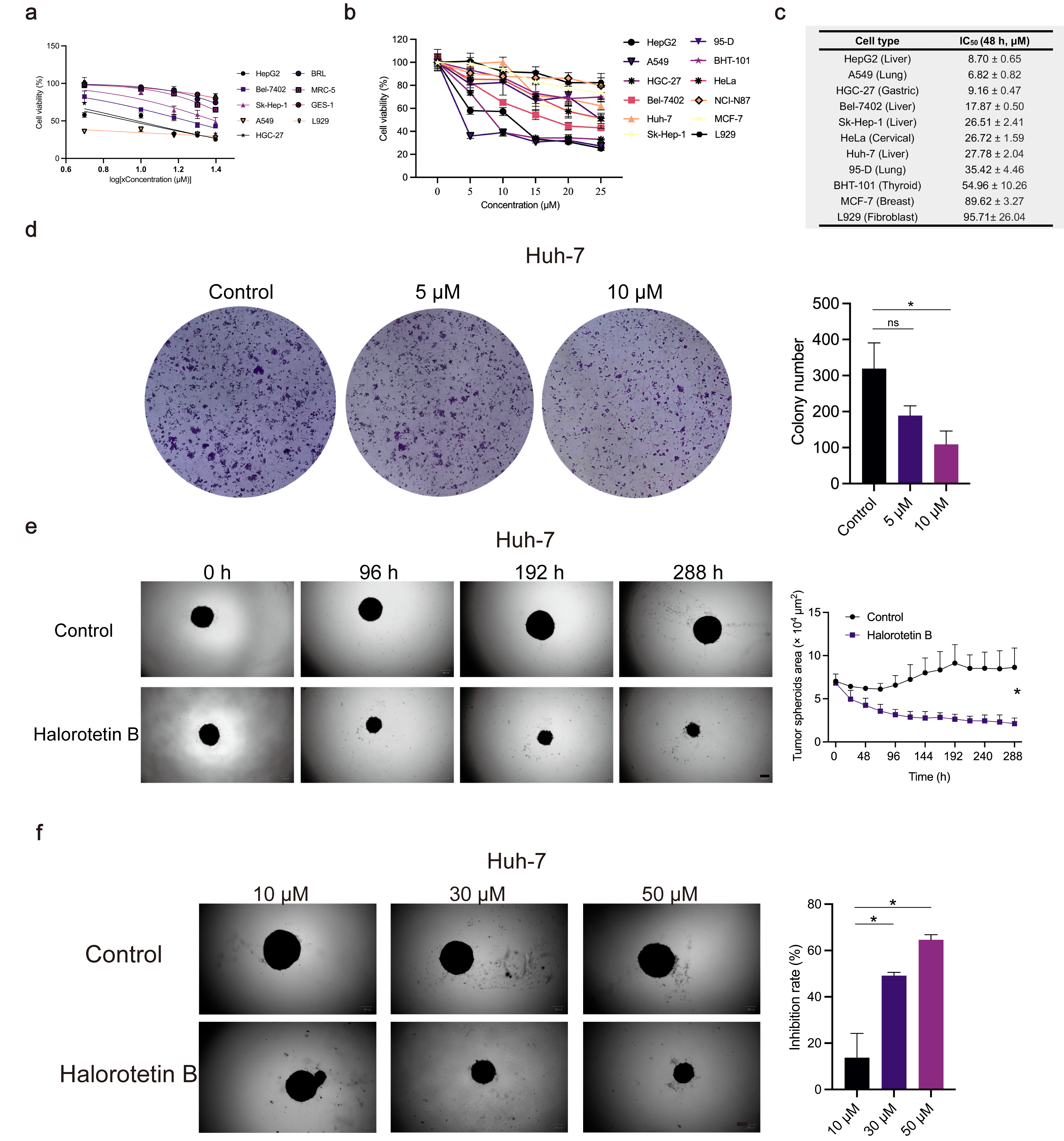


**Figure S3. Halorotetin B inhibits the growth of tumor cell and tumor spheroid.**

**(a)** Fitting curve for calculating IC_50_ value of tumor cell lines and non-maglinant cell lines. (**b**) The inhibitory effect of Halorotetin B on different tumor cell lines after treatment with Halorotetin B for 48 h. (**c**) The IC_50_ value of Halorotetin B on different tumor cell lines. (**d**) Halorotetin B decreases colony formation ability of Huh-7 cells, the cells were stained with crystal violet and quantified. Data are presented as mean ± SD. Significance was determined by one-way ANOVA, * *p* < 0.05, ns *p* > 0.05, n = 3 biologically independent samples. (**e)** Halorotetin B (60 μM) inhibits the growth of Huh-7 tumor spheroids, scale bar = 200 μm. Data are presented as mean ± SD. Significance was determined by two-way ANOVA, * *p* < 0.05, n = 3 biologically independent samples. (f) Halorotetin B inhibited the proliferation of tumor spheroids in a dose-dependent manner. In the statistical data, the treatment time with Halorotetin B was 288 h, scale bar = 200 μm. Data are presented as mean ± SD. Significance was determined by one-way ANOVA, * *p* < 0.05, n = 3 biologically independent samples.


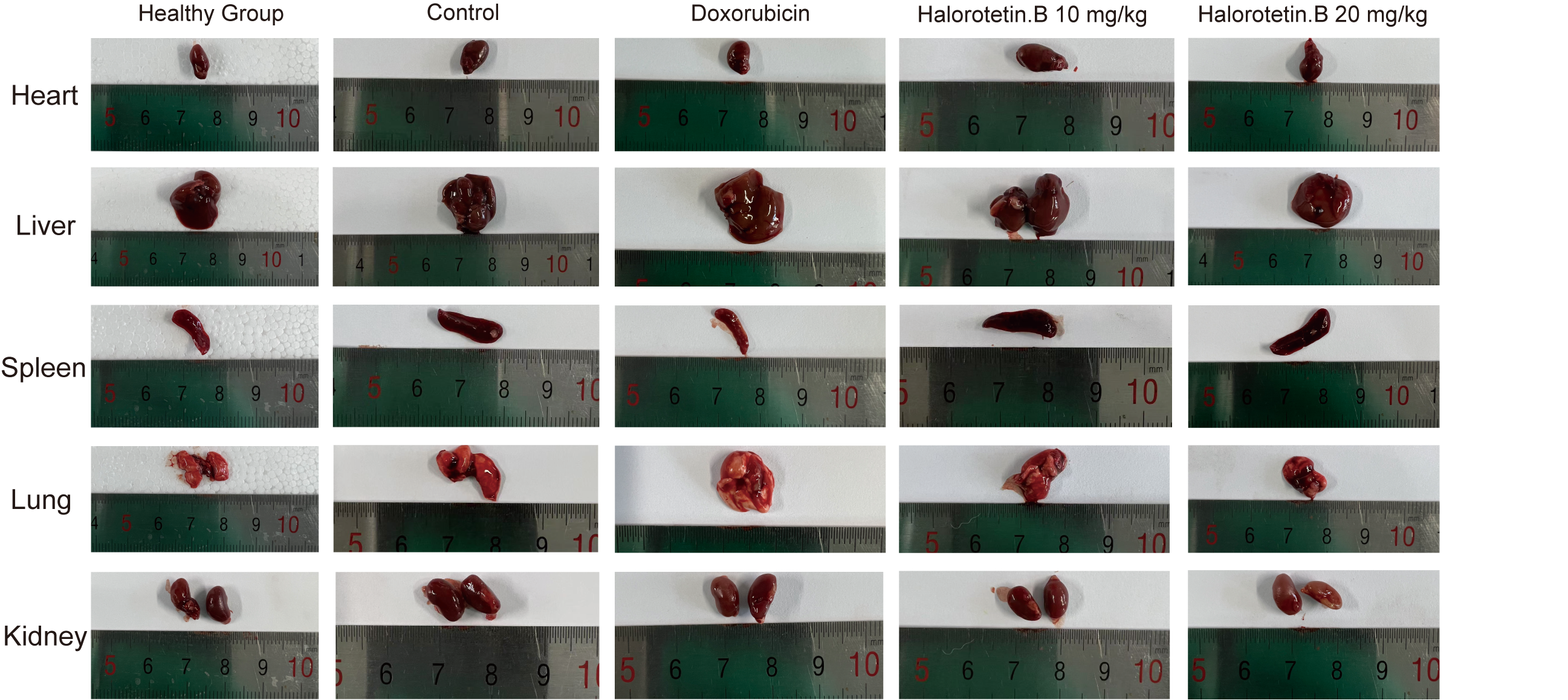


**Figure S4. Halorotetin B inhibits tumor progression.**

The images of heart, liver, spleen, lung, and kidney of xenograft mouse models. No significant abnormalities were observed after Halorotetin B treatment.


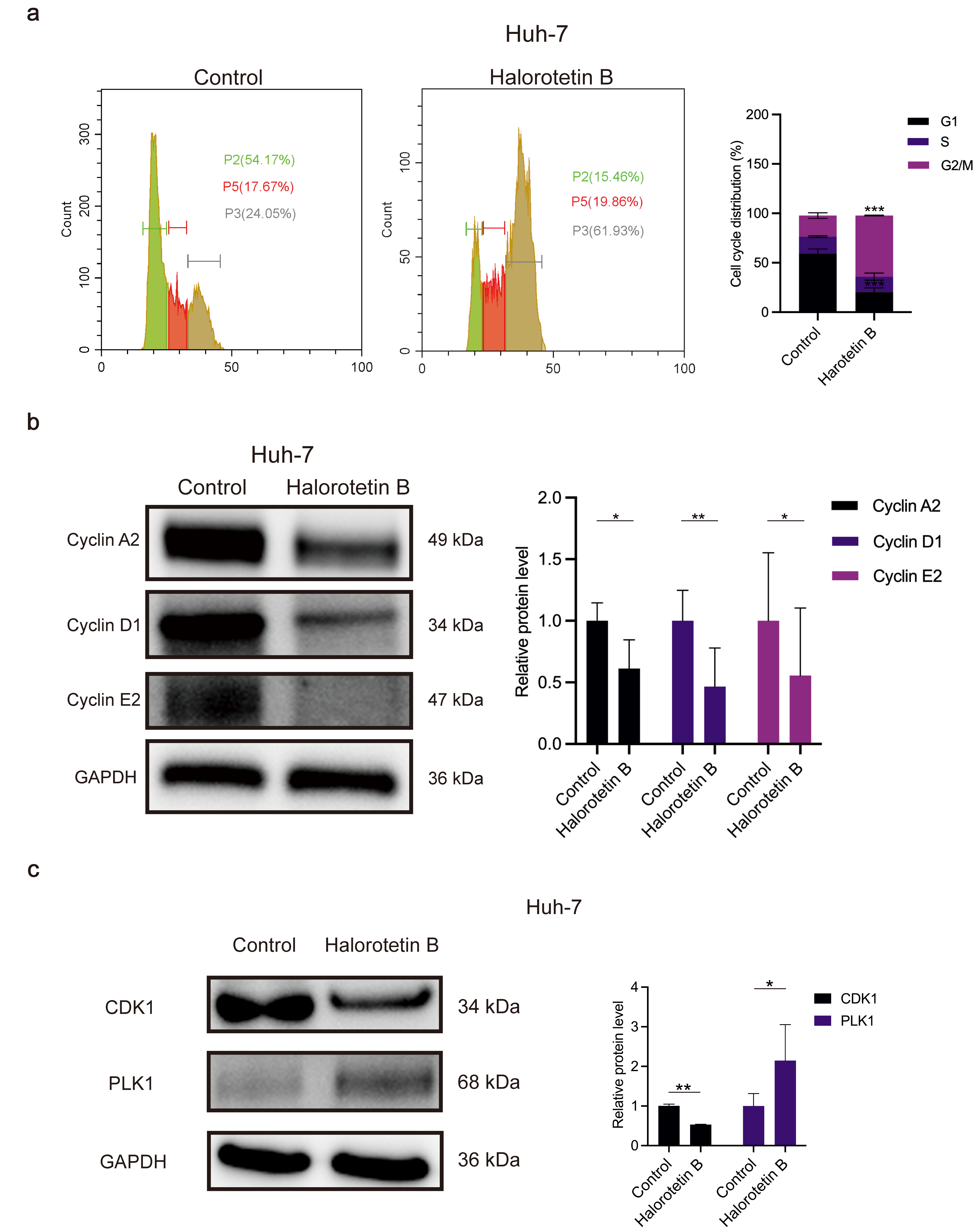


**Figure S5. Halorotetin B induces cell cycle arrest of tumor cells.**

(**a**) The cell cycle distribution was analyzed by flow cytometry, Halorotetin B concentration: 20 μM, and Halorotetin B treatment time: 24 h. Data are presented as mean ± SD. Significance was determined by one-way ANOVA, *** *p* < 0.001, n = 3 biologically independent samples. (**b**) Western blot analysis of G1 or S phase cell cycle-associated proteins in Huh-7 cells exposed to Halorotetin B (20 μM) for 24 h. Data are presented as mean ± SD. Significance was determined by two-tailed *t*-test, * *p* < 0.05, ** *p* < 0.01, n = 3 biologically independent samples. (c) Western blot analysis of the expression level of G2 or M phase cell cycle-associated proteins in Huh-7 cells exposed to Halorotetin B (20 μM) treatment for 24 h. Data are presented as mean ± SD. Significance was determined by two-tailed *t*-test, * *p* < 0.05, ** *p* < 0.01, n = 3 biologically independent samples.


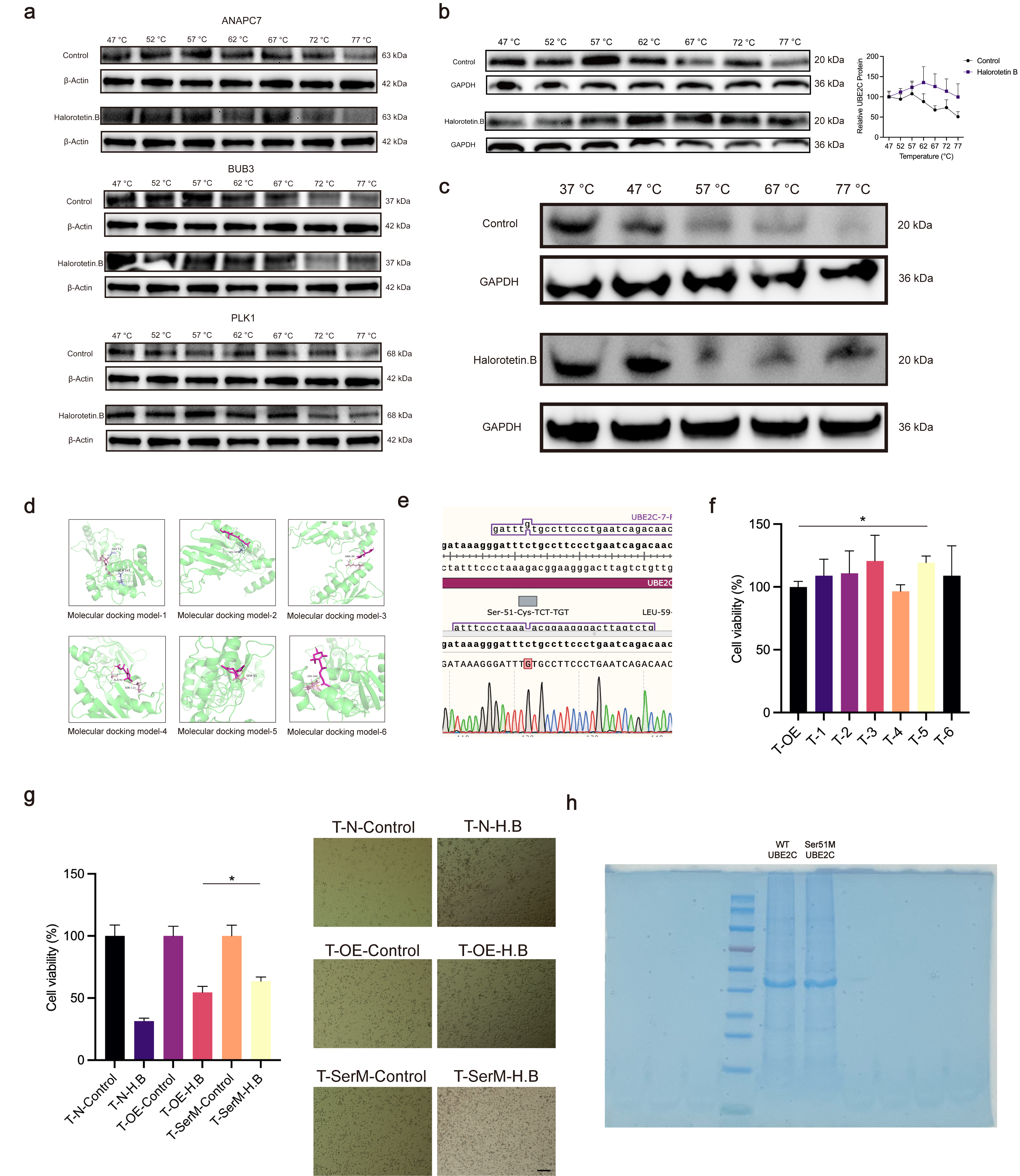


**Figure S6. UBE2C is a direct target of Halorotetin B.**

(**a**) The most potential candidate targets were analyzed by CETSA. (**b**) The CETSA assay determined the thermal stabilization of UBE2C interaction with Halorotetin B (20 μM) in Huh-7 cells. Halorotetin B treatment time: 24 h. (**c**) The CETSA assay in tumor tissues. For the Halorotetin B treatment group, the dose of 20 mg/kg was chosen. (**d**) Molecular docking models with docking sites from the top 10 docking models with the smallest docking energy. (**e**) The Ser51 of UBE2C was mutated into cysteine. (**f**) The inhibitory effect of Halorotetin B on HEK-293T cells after treatment with 20 μM Halorotetin B for 24 h with the transfection of different plasmids. T-OE: Transfection with UBE2C overexpression plasmid. T-1: Transfection with model 1 amino acid mutation plasmid. T-2: Transfection with model 2 amino acid mutation plasmid. T-3: Transfection with model 3 amino acid mutation plasmid. T-4: Transfection with model 4 amino acid mutation plasmid. T-5: Transfection with model 5 amino acid mutation plasmid. T-6: Transfection with model 6 amino acid mutation plasmid. Data are presented as mean ± SD. Significance was determined by one-way ANOVA, * *p* < 0.05, n = 3 biologically independent samples. (**g**) The inhibitory effect of Halorotetin B on HepG2 cells after treatment with 20 μM Halorotetin B for 24 h with the transfection of different plasmids. T-N: HEK-293T cells without plasmid transfection. T-OE: HEK-293T cells with UBE2C overexpression plasmid transfection. T-SerM: HEK-293T cells with Ser51 mutation plasmid transfection. Data are presented as mean ± SD. Significance was determined by two-way ANOVA, * *p* < 0.05, n = 3 biologically independent samples. (**h**) The purity of wild-type UBE2C protein (left) and Ser51-mutant UBE2C protein (right).


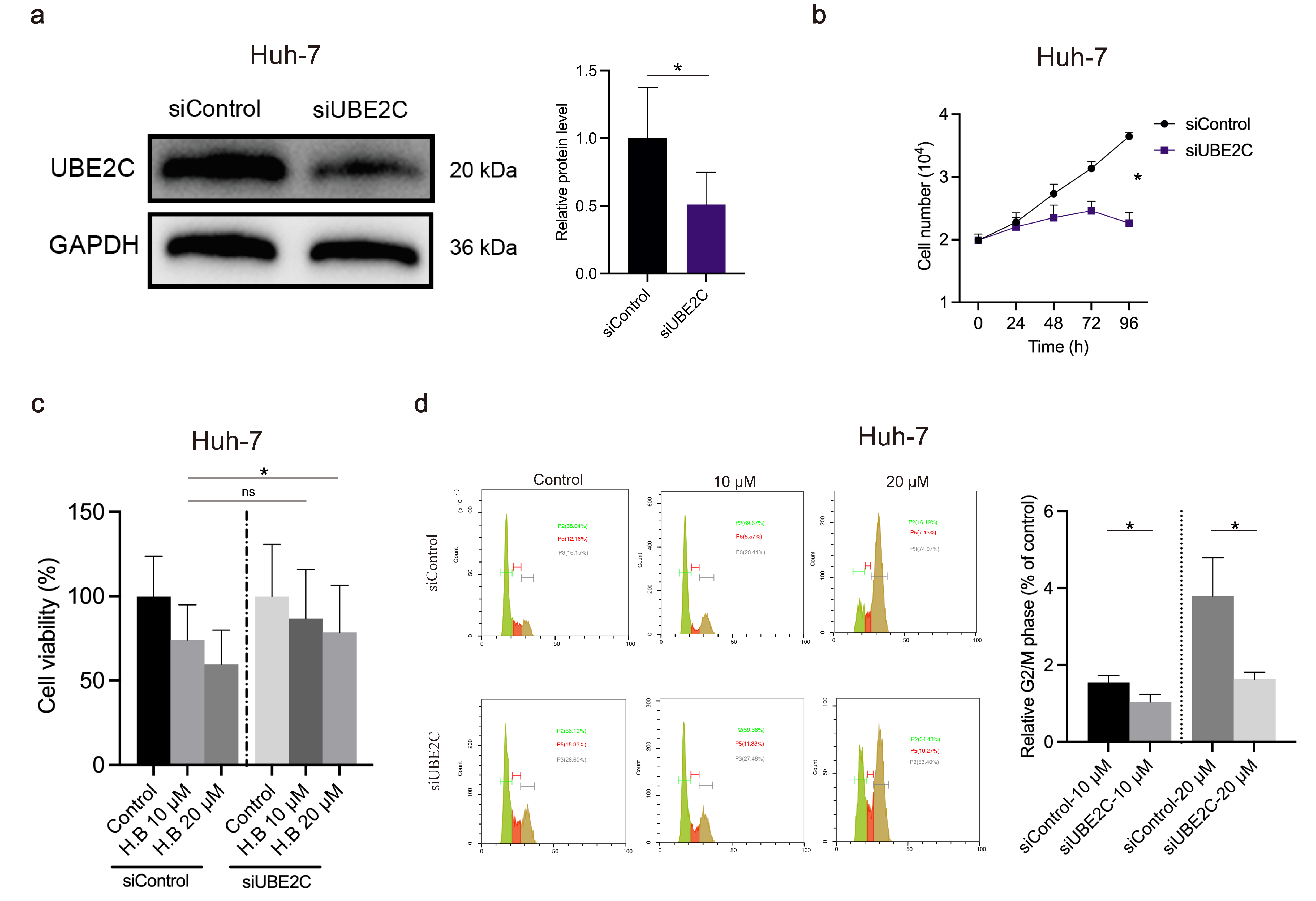


**Figure S7. UBE2C overexpression is associated with tumor progression and anti-tumor efficacy of Halorotetin B.**

(**a**) The protein level of UBE2C after siRNA treatment for 48 h. The concentration of control siRNA and UBE2C siRNA: 100 pM. Data are presented as mean ± SD. Significance was determined by two-tailed *t*-test, * *p* < 0.05, n = 3 biologically independent samples. (**b**) Influence of siRNA knockdown of UBE2C on Huh-7 cells proliferation. Data are presented as mean ± SD. Significance was determined by two-way ANOVA, * *p* < 0.05, n = 3 biologically independent samples. (**c**) Cell viability assays in Huh-7 cells transfected with siRNA targeting UBE2C or control siRNA. Halorotetin B treatment time: 48 h. Data are presented as mean ± SD. Significance was determined by two-way ANOVA, ns *p* > 0.05, * *p* < 0.05, n = 3 biologically independent samples. (**d**) Cell cycle distribution assays of Halorotetin B on UBE2C wild type cells and UBE2C knockdown cells. The Huh-7 cells were treated with control or the indicated concentrations of Halorotetin B for 24 h. Data are presented as mean ± SD. Significance was determined by two-way ANOVA, * *p* < 0.05, n = 3 biologically independent samples.


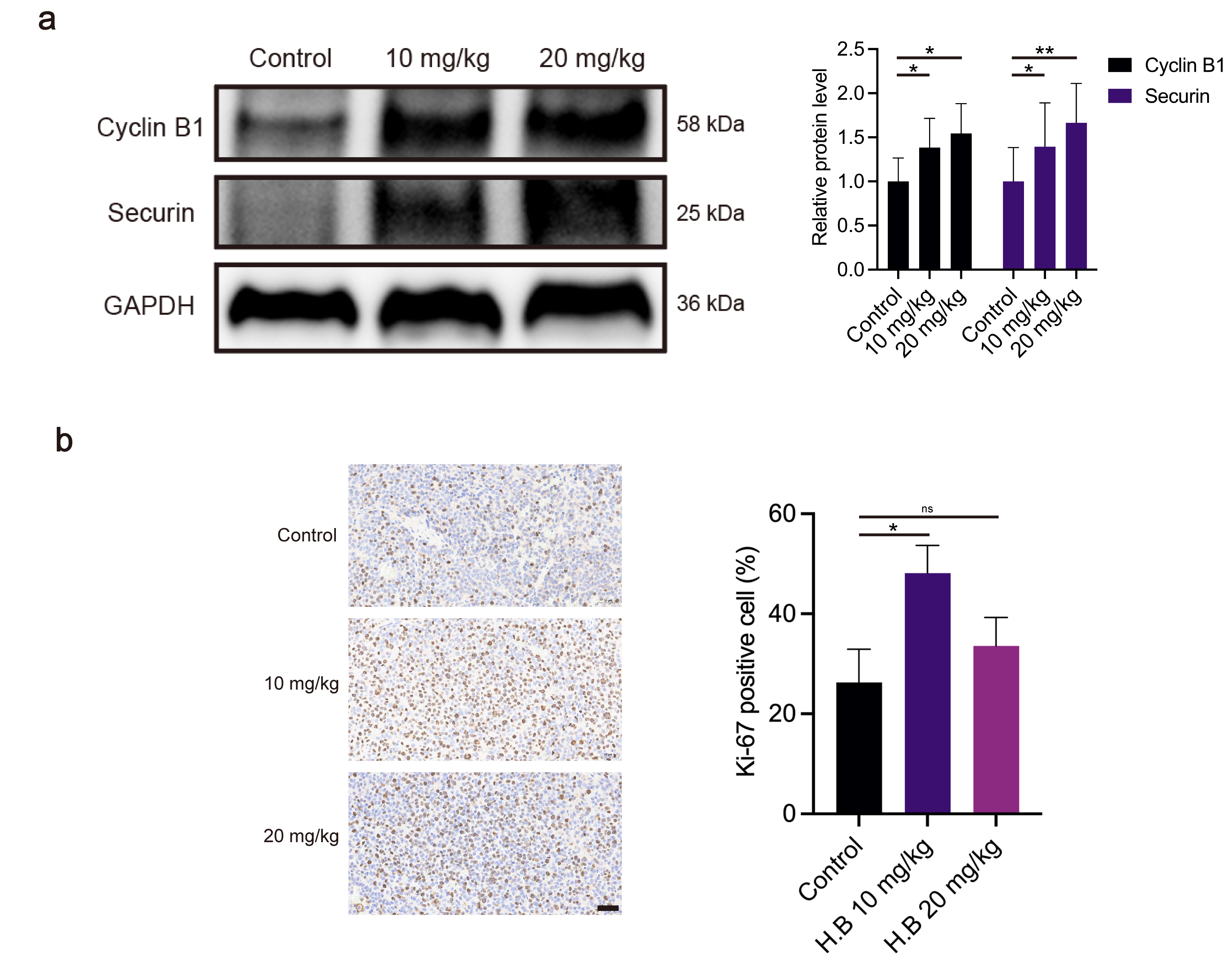


**Figure S8. Halorotetin B arrests tumor cells at the cell cycle M phase.**

(**a**) Western blot analyzed the protein level of cyclin B1 and securin in tumor tissues after treatment with Halorotetin B or solvent. Data are presented as mean ± SD. Significance was determined by one-way ANOVA, * *p* < 0.05, ** *p* < 0.01, n = 3 biologically independent samples. (**b**) Representative images of Ki-67 immunohistochemical staining of tumor tissues after treatment with Halorotetin B or solvent, scale bar = 50 μm. The number of Ki-67 positive cells was counted in the whole section. Data are presented as mean ± SD. Significance was determined by one-way ANOVA, ns *p* > 0.05, * *p* < 0.05, n = 3 sections/group.


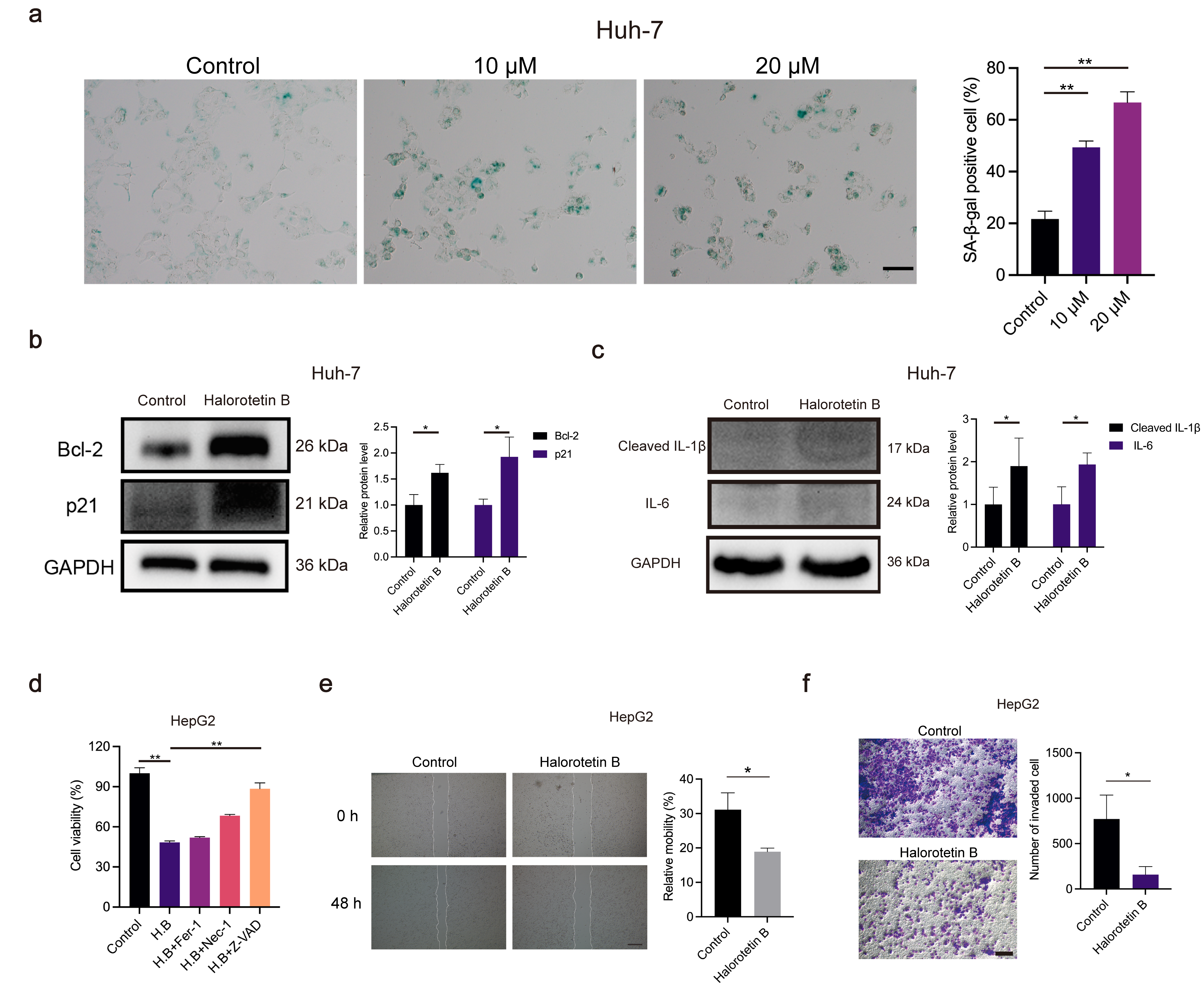


**Figure S9. Halorotetin B induces senescence of tumor cells.**

(**a**) 𝛽-galactosidase staining and quantification reveals that treated with Halorotetin B for 24 h induced cell senescence of Huh-7 cells, scale bar = 100 μm. Data are presented as mean ± SD. Significance was determined by one-way ANOVA, ** *p* < 0.01, n = 3 biologically independent samples. (**b**) Western blot analyzed the cell senescence associated proteins level treated with Halorotetin B (20 μM) for 24 h. Data are presented as mean ± SD. Significance was determined by two-tailed *t*-test, * *p* < 0.05, n = 3 biologically independent samples. (**c**) Western blot analyzed the senescence-associated secretory phenotype (SASP) level treated with Halorotetin B (20 μM) for 24 h. Data are presented as mean ± SD. Significance was determined by two-tailed *t*-test, * *p* < 0.05, n = 3 biologically independent samples. (**d**) The inhibitory effect of Halorotetin B on HepG2 cells in the presence of ferrostatin-1 (Fer-1, 2 μM), necostatin (Nec-1, 1 μM), or Z-VAD-FMK (Z-VAD, 10 μM). Halorotetin B treatment time: 48 h. Data are presented as mean ± SD. Significance was determined by two-way ANOVA, ** *p* < 0.01, n = 3 biologically independent samples. (**e**) Treatment with Halorotetin B (5 μM) for 24 h decreases migration ability of HepG2 cells, the white box represents area of cell scratch, scale bar = 200 μm. Data are presented as mean ± SD. Significance was determined by two-tailed *t*-test, * *p* < 0.05, n = 3 biologically independent samples. (**f**) Treatment with Halorotetin B (5 μM) for 24 h decreases invasion ability of HepG2 cells, scale bar = 200 μm. Data are presented as mean ± SD. Significance was determined by two-tailed *t*-test, * *p* < 0.05, n = 3 biologically independent samples.
