# Supplementary material for: Halorotetin B, A Novel Terpenoid Compound Derived from Marine Ascidian, Suppresses Tumor Growth by Targeting the Cell Cycle Regulator UBE2C
Source: Adv Sci (Weinh). 2025 Dec 12;13(12):e15652. doi: 10.1002/advs.202515652 (PMC12948284; doi:10.1002/advs.202515652)
Supplement: Supplementary file 3 — Supporting Information [file ADVS-13-e15652-s002.pdf]

Supporting Information

Halorotetin B, a novel terpenoid compound derived from marine ascidian, suppresses tumor growth by targeting the cell cycle regulator UBE2C

Shanhao Han[1], Jianhui Li[1], Yuting Zhu[1], Penghui Liu[1], Yaoyao Zheng[3], Muchun He[4], and Bo Dong[1,2,5,*]

[1]Fang Zongxi Center for Marine EvoDevo, MoE Key Laboratory of Marine Genetics and Breeding, College of Marine Life Sciences, Ocean University of China, Qingdao 266003, China

[2]Laboratory for Marine Biology and Biotechnology, Qingdao National Laboratory for Marine Science and Technology, Qingdao 266237, China

[3]Department of Pharmacy, Affiliated Hospital of Shandong University of Traditional Chinese Medicine, Jinan 250011, China

[4]Liaoning Key Laboratory of Marine Animal Immunology and Disease Control, Dalian Ocean University, Dalian 116023, China

[5]Institute of Evolution & Marine Biodiversity, Ocean University of China, Qingdao 266003, China

*Correspondence: bodong@ouc.edu.cn

Uncropped Blots

In the western blot analyses for manuscript entitled "Halorotetin B, a novel terpenoid compound derived from marine ascidian, suppresses tumor growth by targeting the cell cycle regulator UBE2C", all protein markers were obtained from Vazyme Biotech (Cat. No. MP102-01). The relevant molecular weights are shown in the following picture:

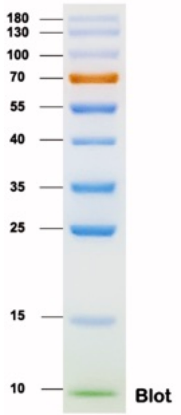

Cell cycle associated proteins:

HepG2 cell

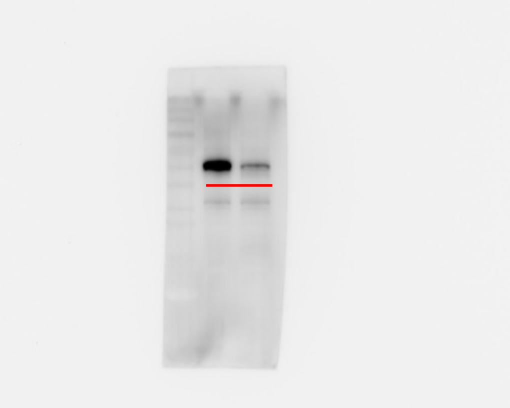
Cyclin A2-HepG2-1

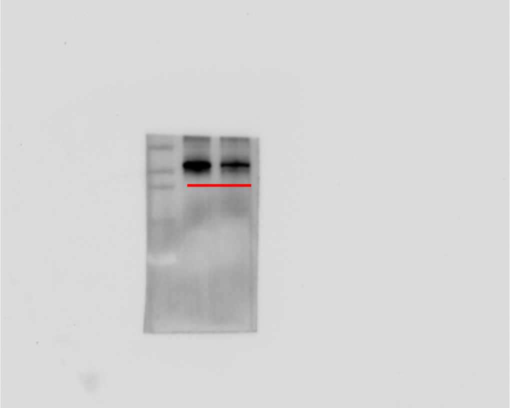
Cyclin D1-HepG2-1

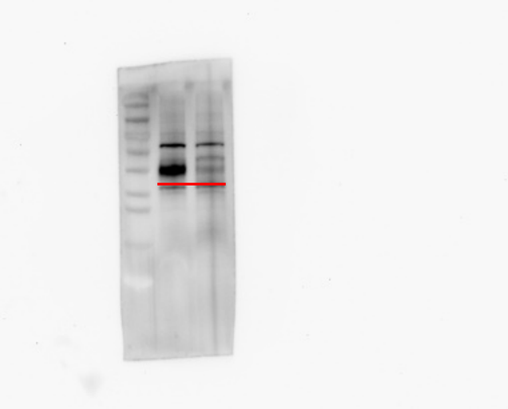
Cyclin E2-HepG2-1

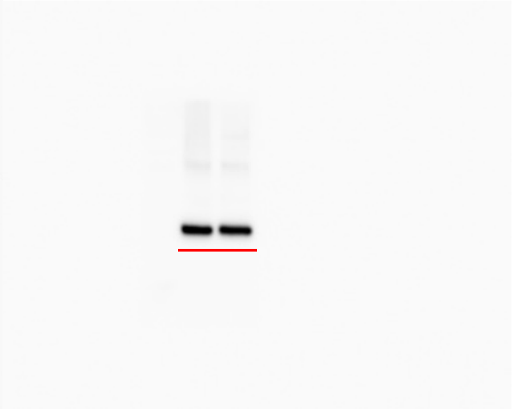
GAPDH-HepG2-1

The first independent replicate experiment (-1) was used to present in the manuscript, which related to Fig. 4d.

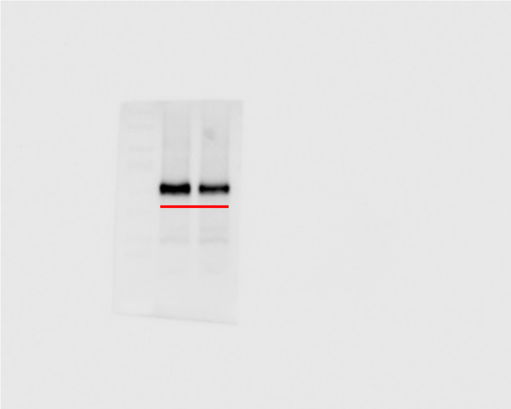
Cyclin A2-HepG2-2

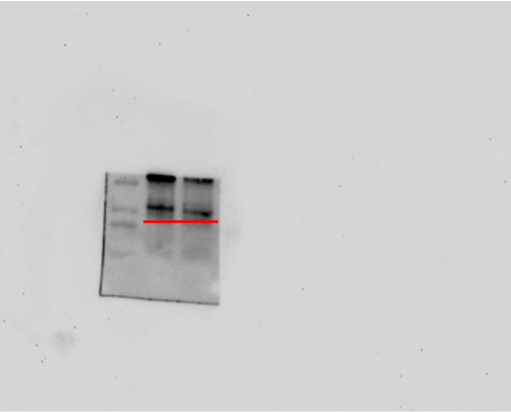
Cyclin D1-HepG2-2

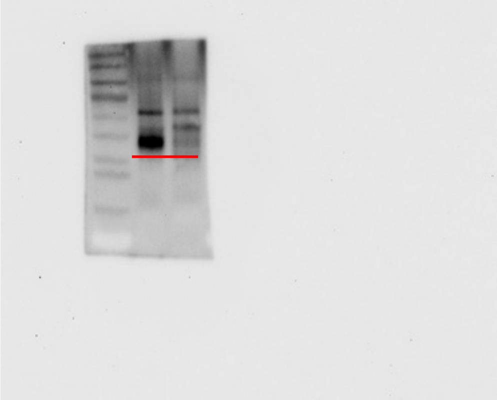

Cyclin E2-HepG2-2

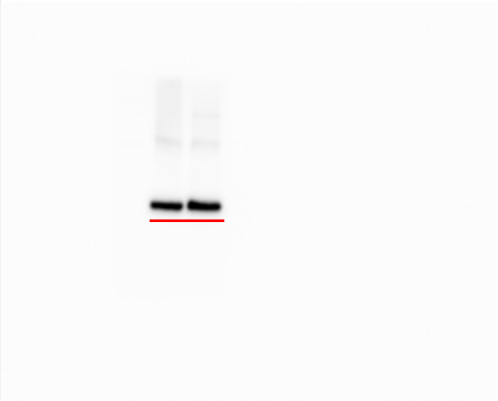

GAPDH-HepG2-2

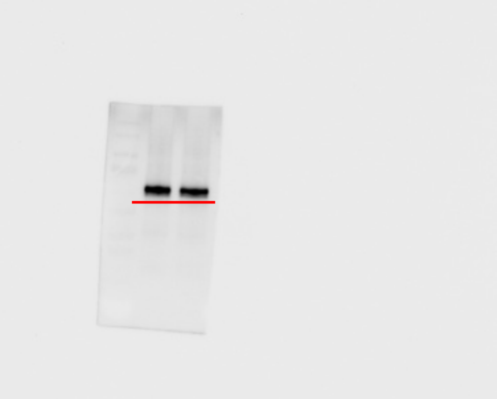

Cyclin A2-HepG2-3

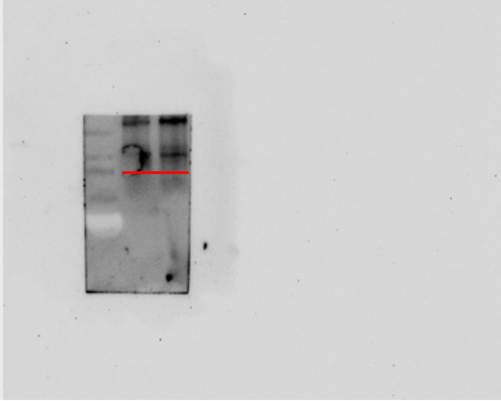

Cyclin D1-HepG2-3

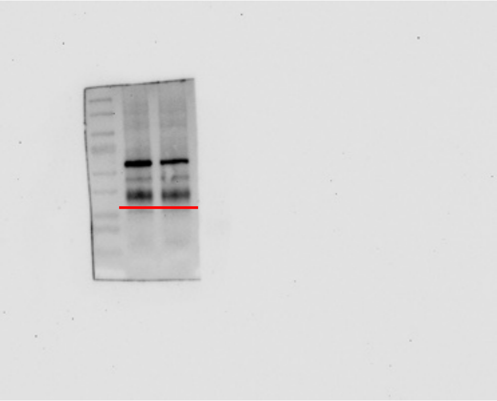

Cyclin E2-HepG2-3

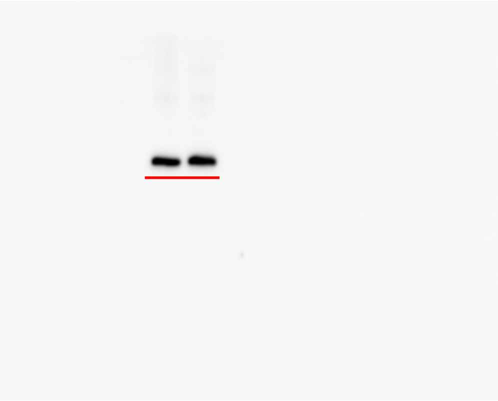

GAPDH-HepG2-3

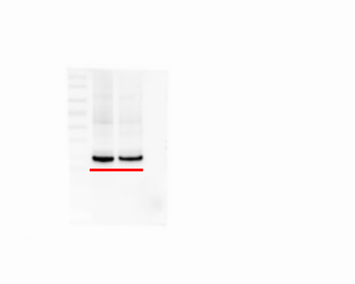

CDK1-HepG2-1

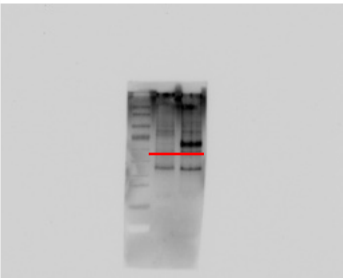

PLK1-HepG2-1

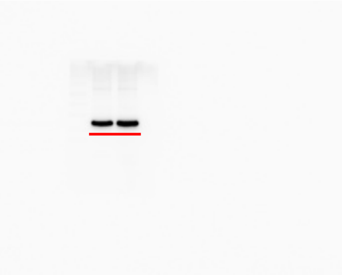

GAPDH-HepG2-1

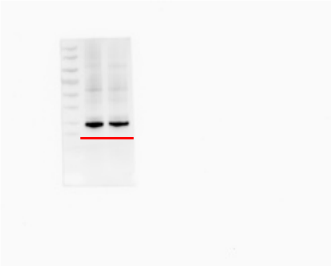

CDK1-HepG2-2

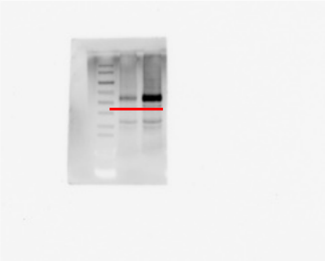

PLK1-HepG2-2

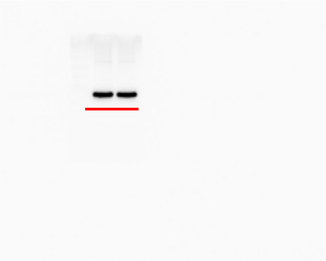

GAPDH-HepG2-2

The second independent replicate experiment (-2) was used to present in the manuscript, which related to Fig. 4e.

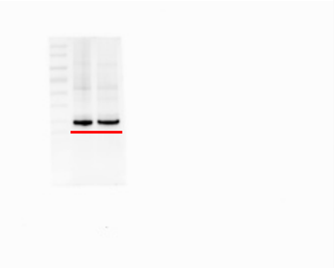

CDK1-HepG2-3

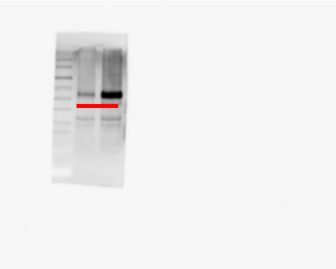

PLK1-HepG2-3

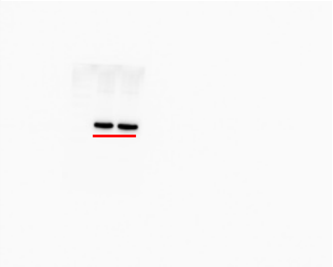

GAPDH-HepG2-3

Bel-7402 cell

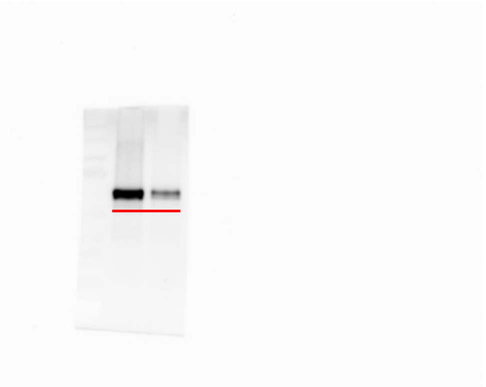

Cyclin A2-Bel-7402-1

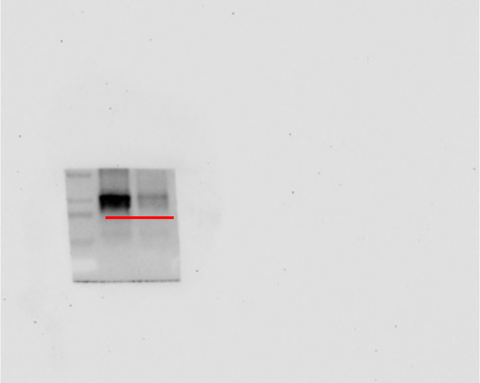

Cyclin D1-Bel-7402-1

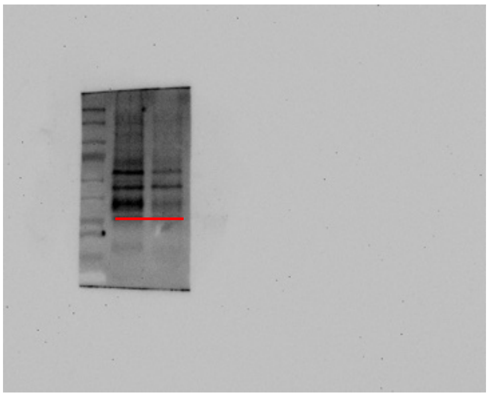

Cyclin E2-Bel-7402-1

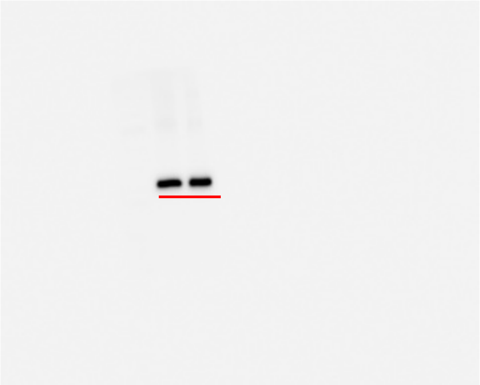

GAPDH-Bel-7402-1

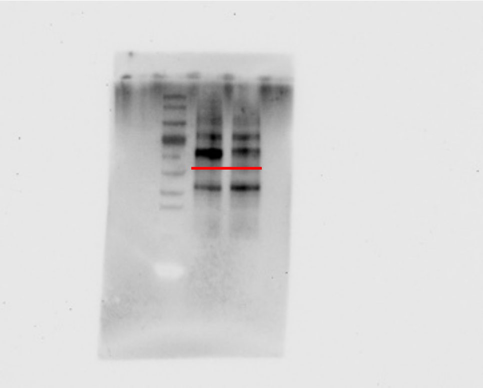

Cyclin A2-Bel-7402-2

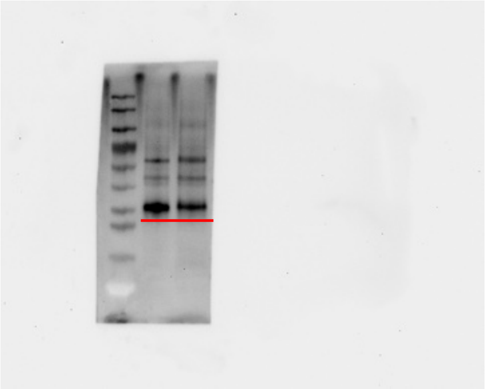

Cyclin D1-Bel-7402-2

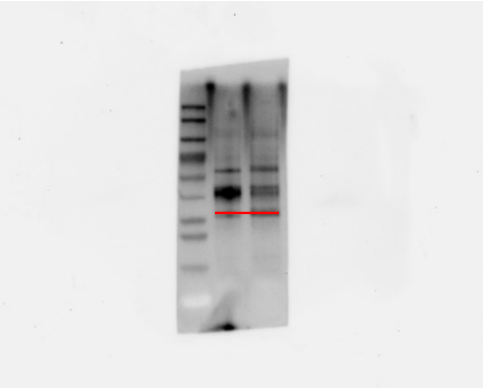

Cyclin E2-Bel-7402-2

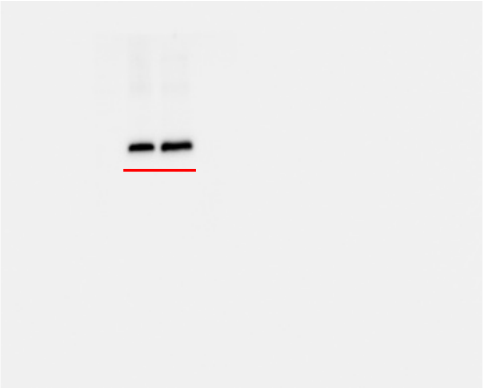

GAPDH-Bel-7402-2

The second independent replicate experiment (-2) was used to present in the manuscript, which related to Fig. 4d.

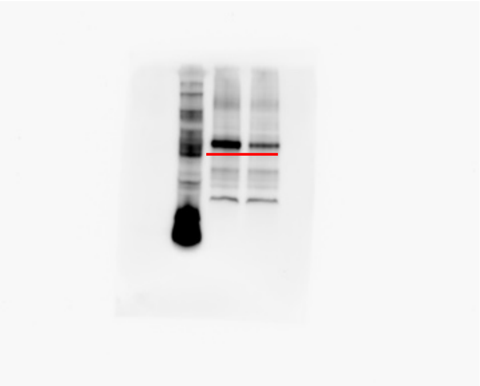

Cyclin A2-Bel-7402-3

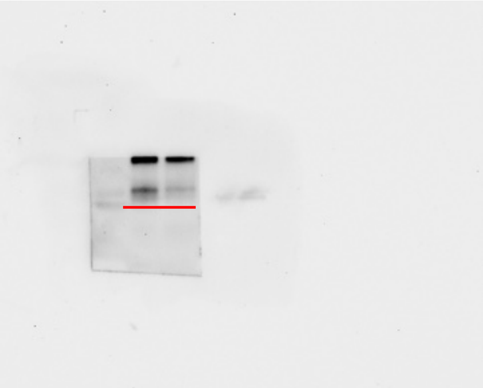

Cyclin D1-Bel-7402-3

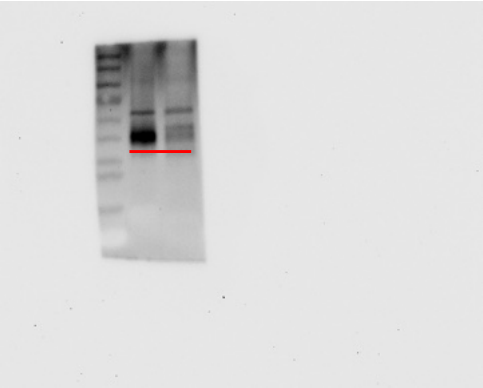

Cyclin E2-Bel-7402-3

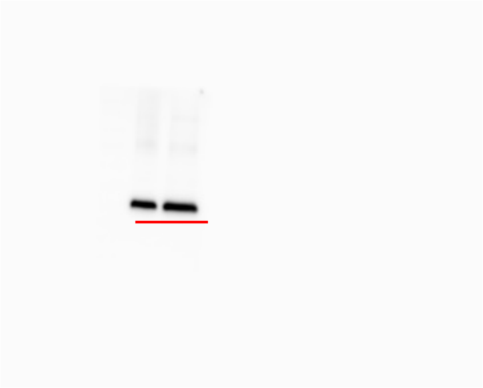

GAPDH-Bel-7402-3

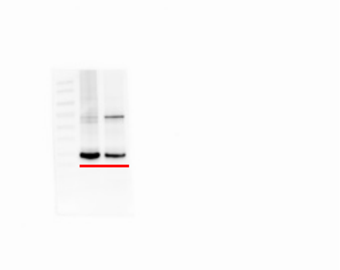

CDK1-Bel-7402-1

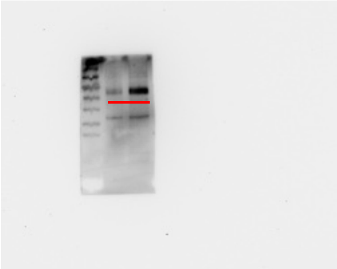

PLK1-Bel-7402-1

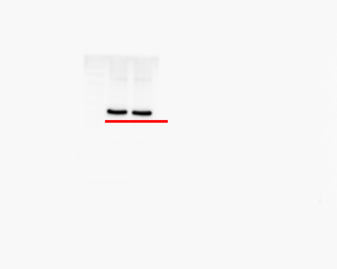

GAPDH-Bel-7402-1

The first independent replicate experiment (-1) was used to present in the manuscript, which related to Fig. 4e.

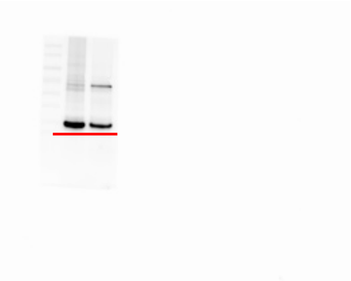

CDK1-Bel-7402-2

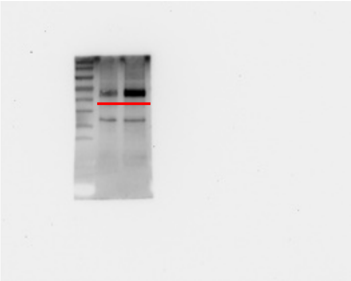

PLK1-Bel-7402-2

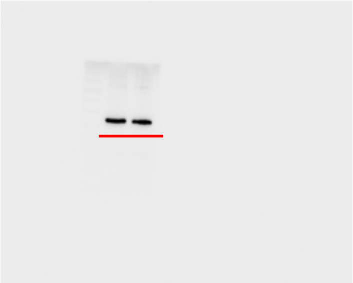

GAPDH-Bel-7402-2

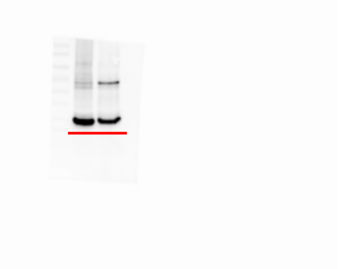

CDK1-Bel-7402-3

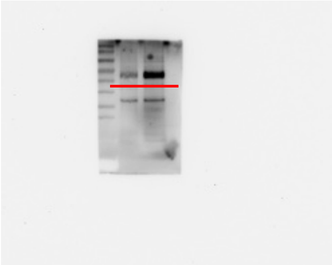

PLK1-Bel-7402-3

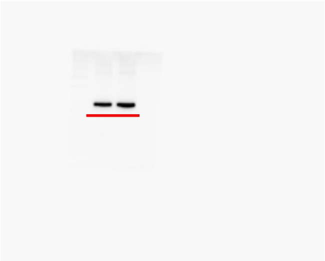

GAPDH-Bel-7402-3

Huh-7 cell

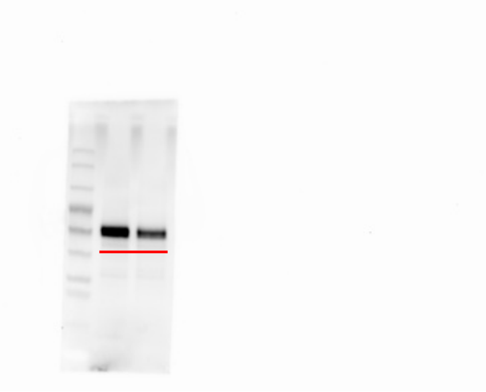

Cyclin A2-Huh-7-1

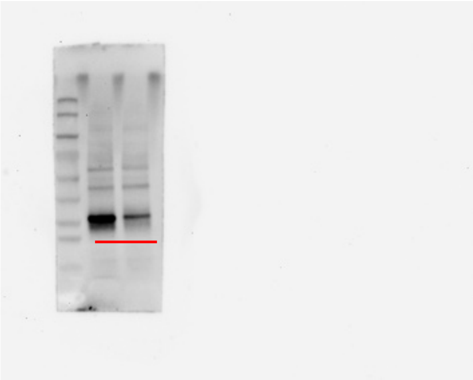

Cyclin D1-Huh-7-1

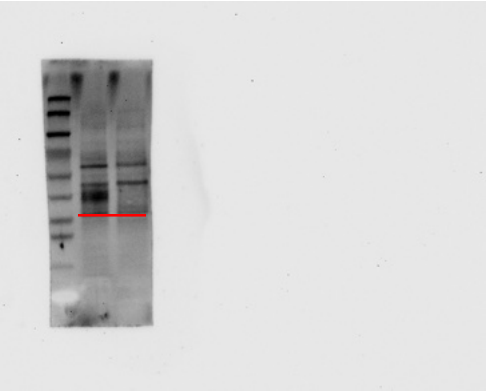

Cyclin E2-Huh-7-1

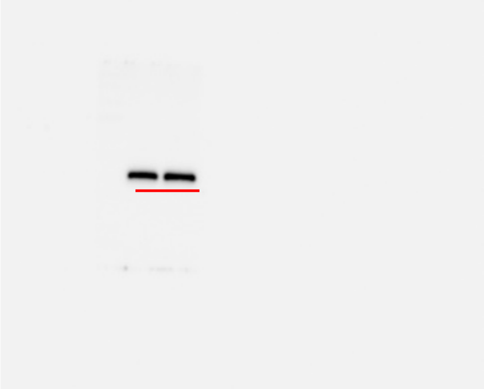

GAPDH-Huh-7-1

The first independent replicate experiment (-1) was used to present in the manuscript, which related to Fig. S5b.

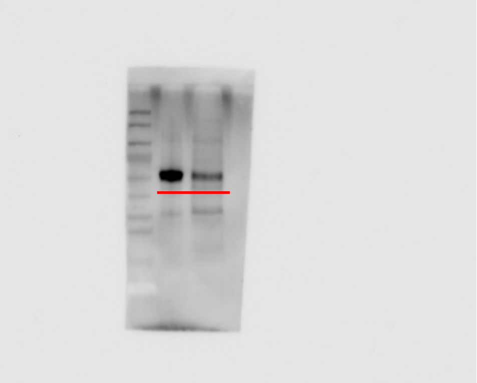

Cyclin A2-Huh-7-2

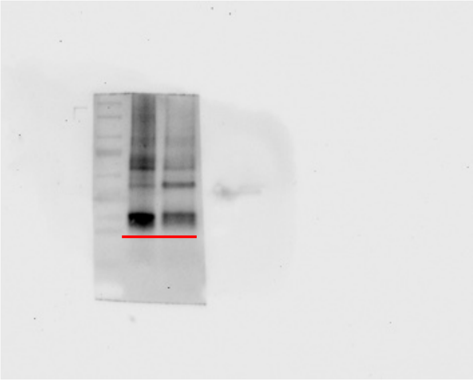

Cyclin D1-Huh-7-2

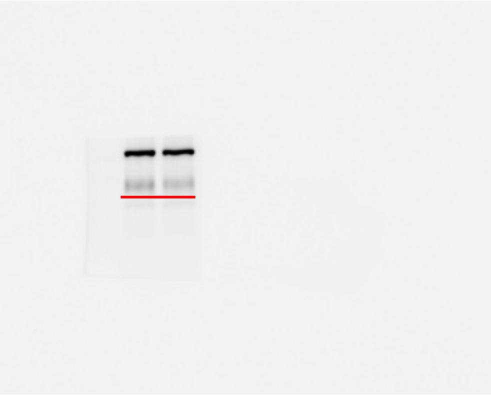

Cyclin E2-Huh-7-2

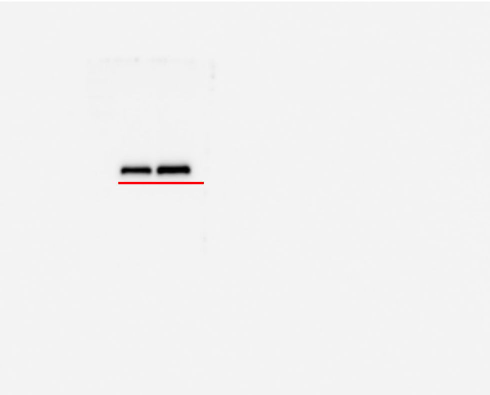

GAPDH-Huh-7-2

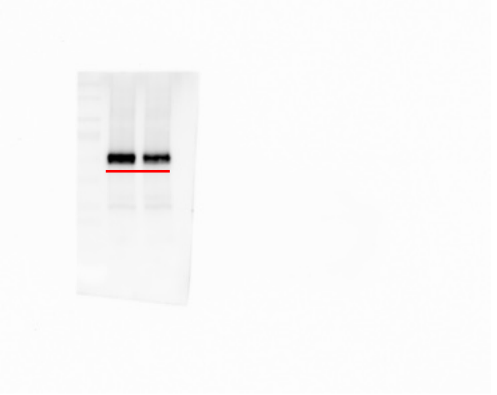

Cyclin A2-Huh-7-3

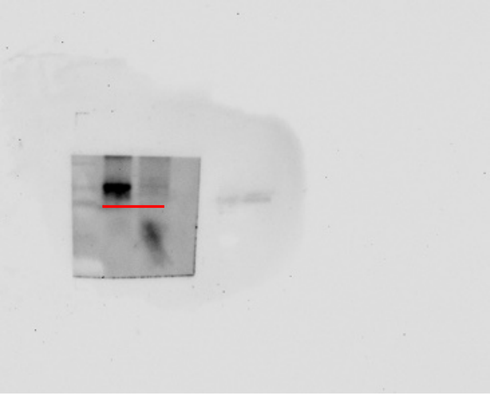

Cyclin D1-Huh-7-3

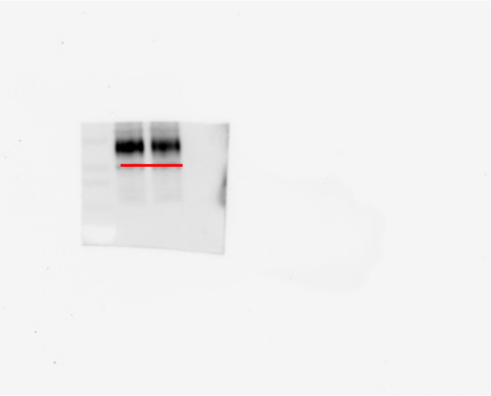

Cyclin E2-Huh-7-3

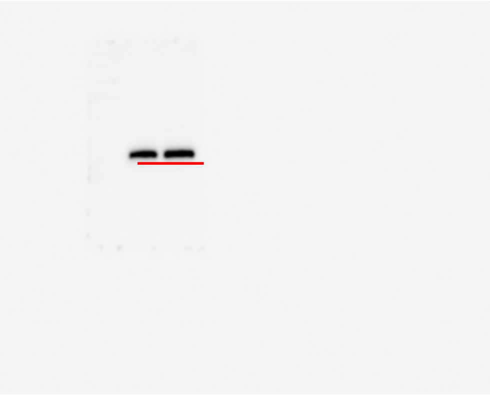

GAPDH-Huh-7-3

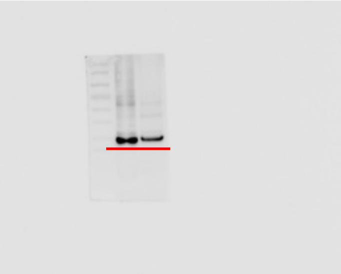

CDK1-Huh-7-1

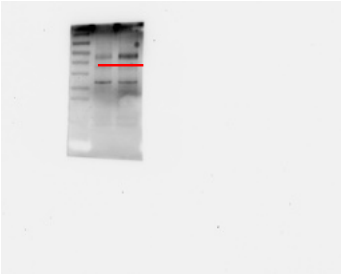

PLK1-Huh-7-1

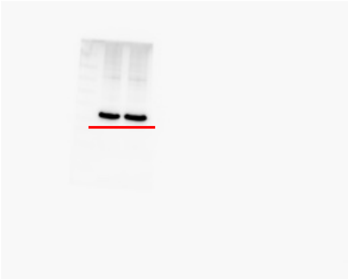

GAPDH-Huh-7-1

The first independent replicate experiment (-1) was used to present in the manuscript, which related to Fig. S5c.

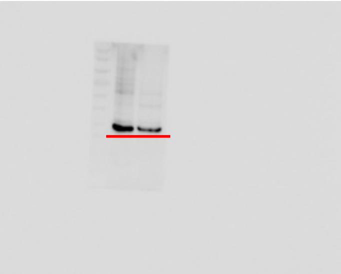

CDK1-Huh-7-2

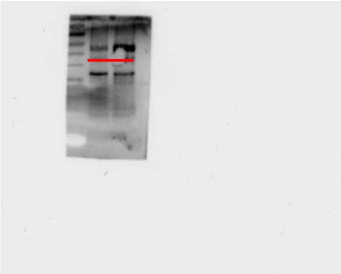

PLK1-Huh-7-2

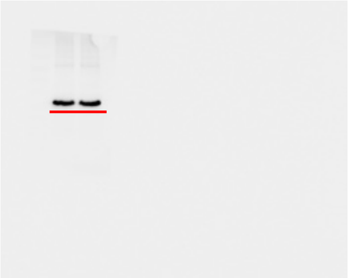

GAPDH-Huh-7-2

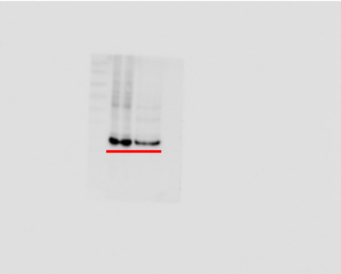

CDK1-Huh-7-3

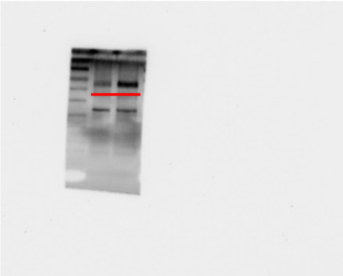

PLK1-Huh-7-3

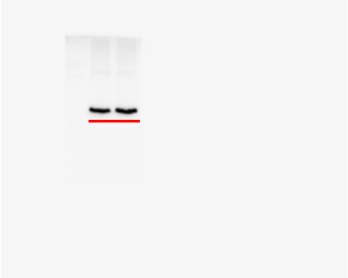

GAPDH-Huh-7-3

Cell cycle associated proteins, related to Fig. 4d, 4e, S5b, and S5c.

Cellular Thermal Shift Assay (CETSA)

HepG2 cell

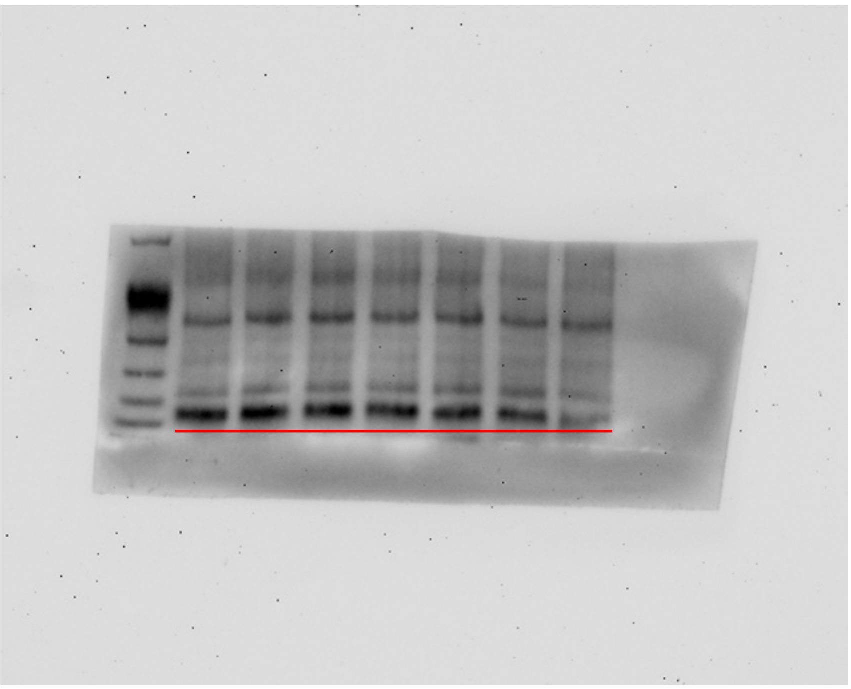

UBE2C-Control-HepG2-1

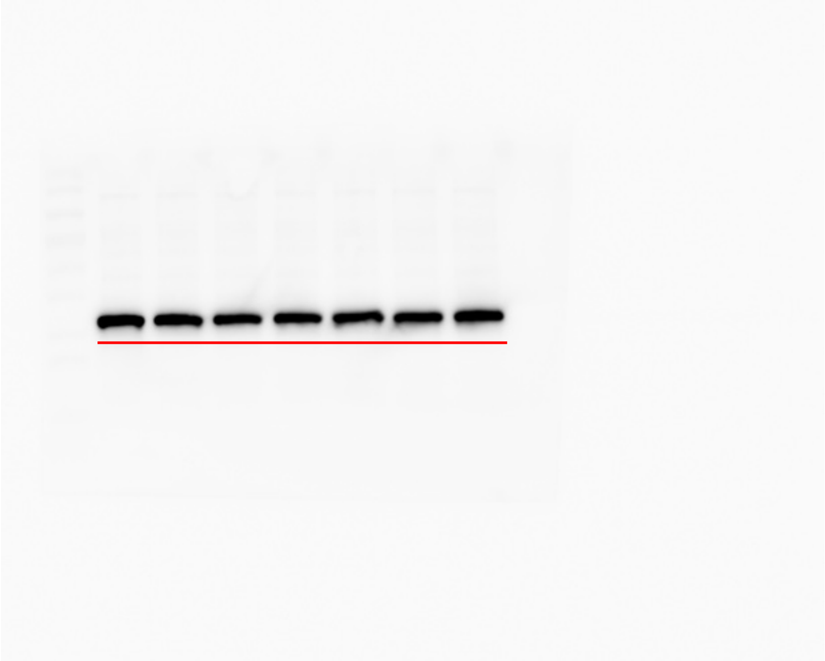

GAPDH-Control-HepG2-1

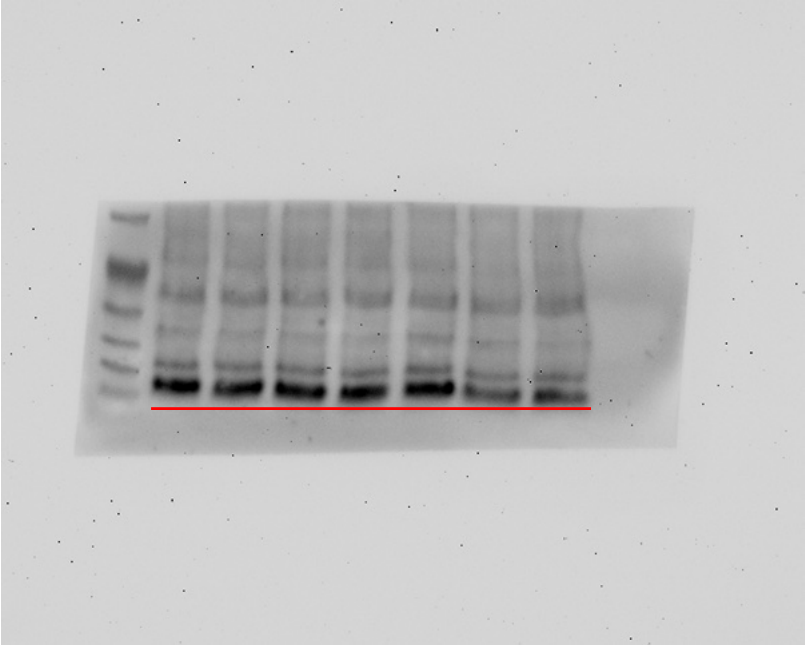

UBE2C-Halorotetin B-HepG2-1

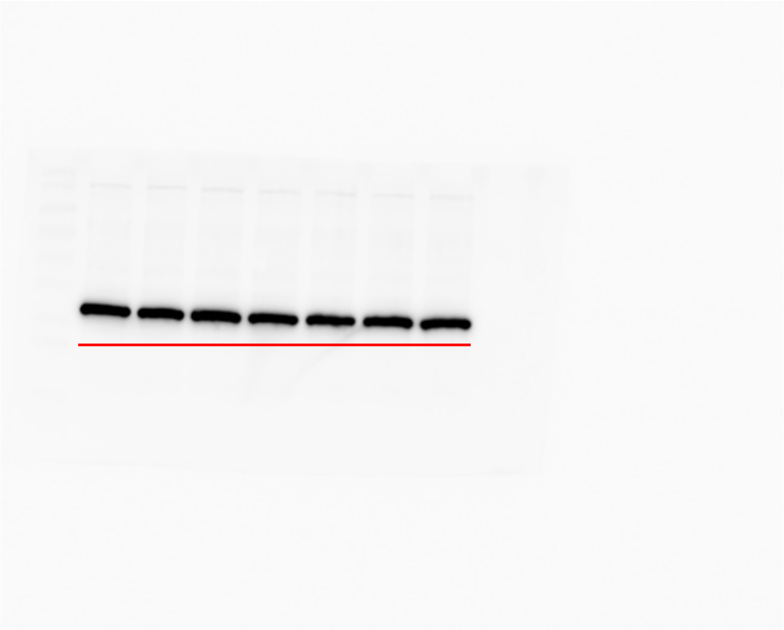

GAPDH-Halorotetin B-HepG2-1

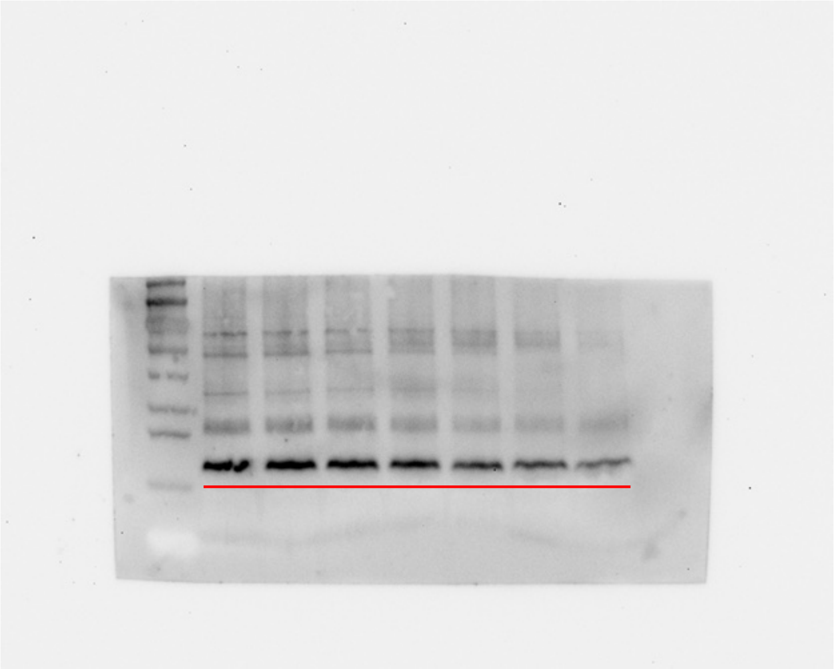

UBE2C-Control-HepG2-2

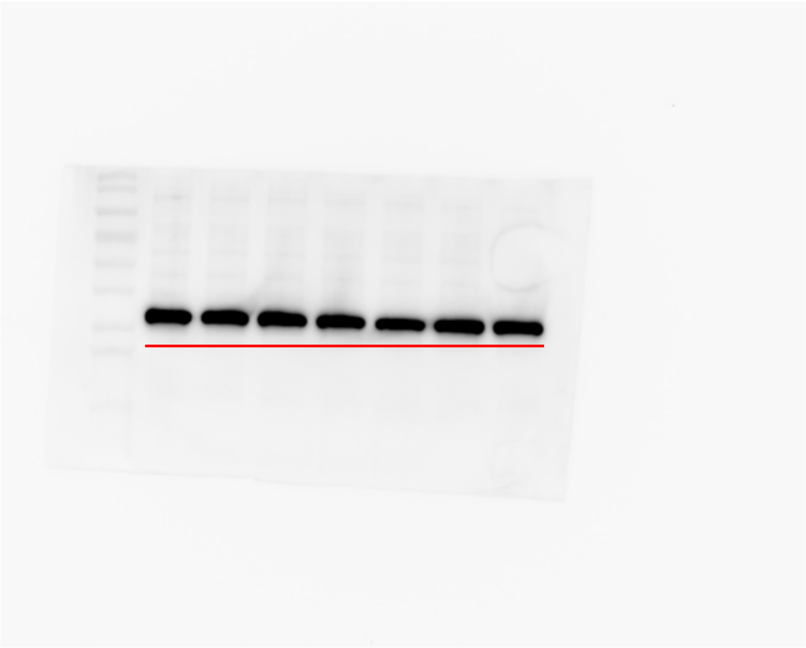

GAPDH-Control-HepG2-2

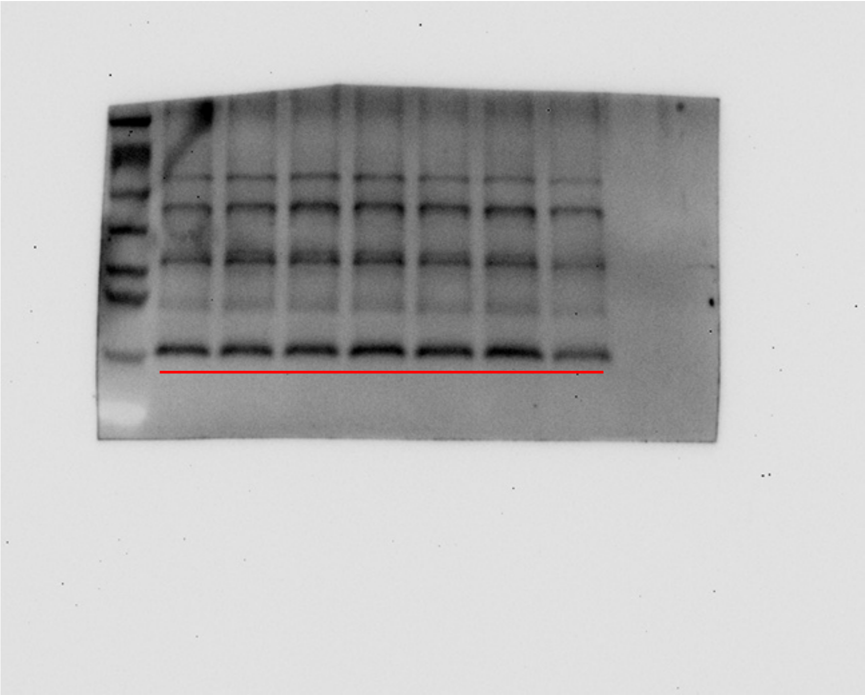

UBE2C-Halorotetin B-HepG2-2

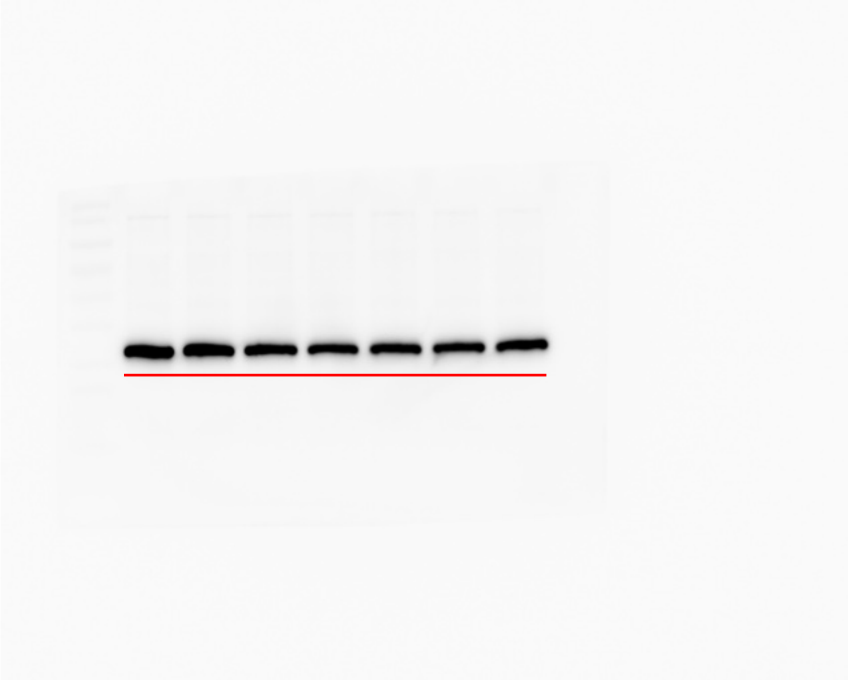

GAPDH-Halorotetin B-HepG2-2

The second independent replicate experiment (-2) was used to present in the manuscript, which related to Fig. 5d.

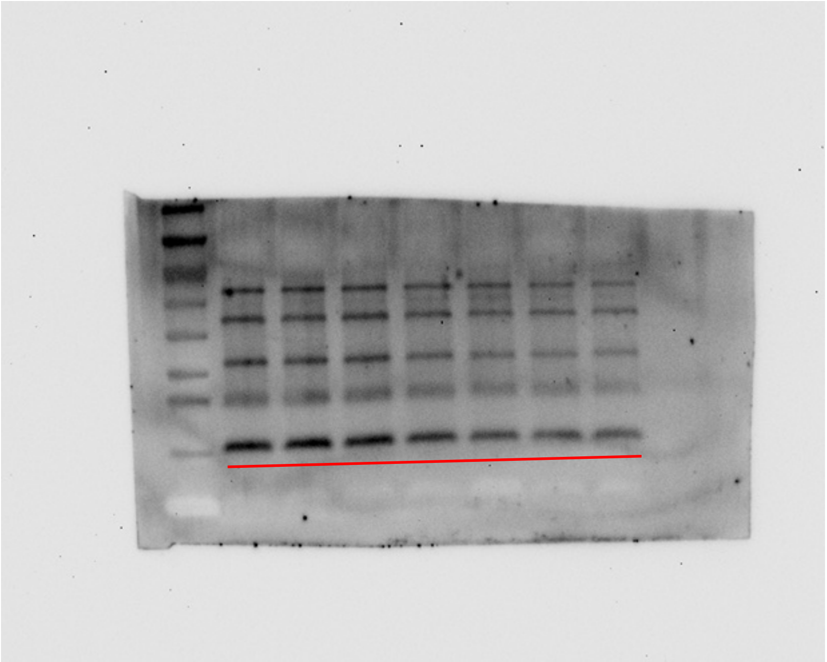

UBE2C-Control-HepG2-3

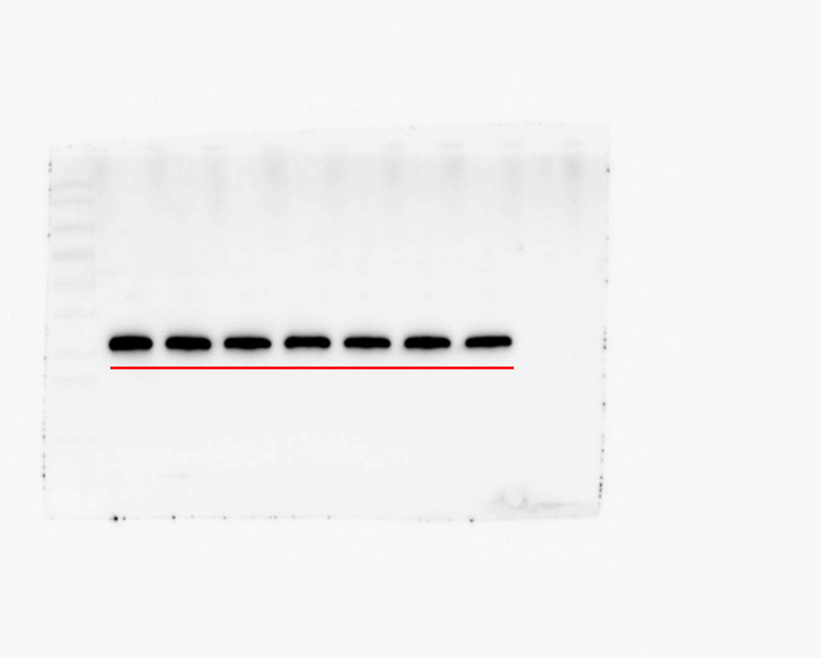

GAPDH-Control-HepG2-3

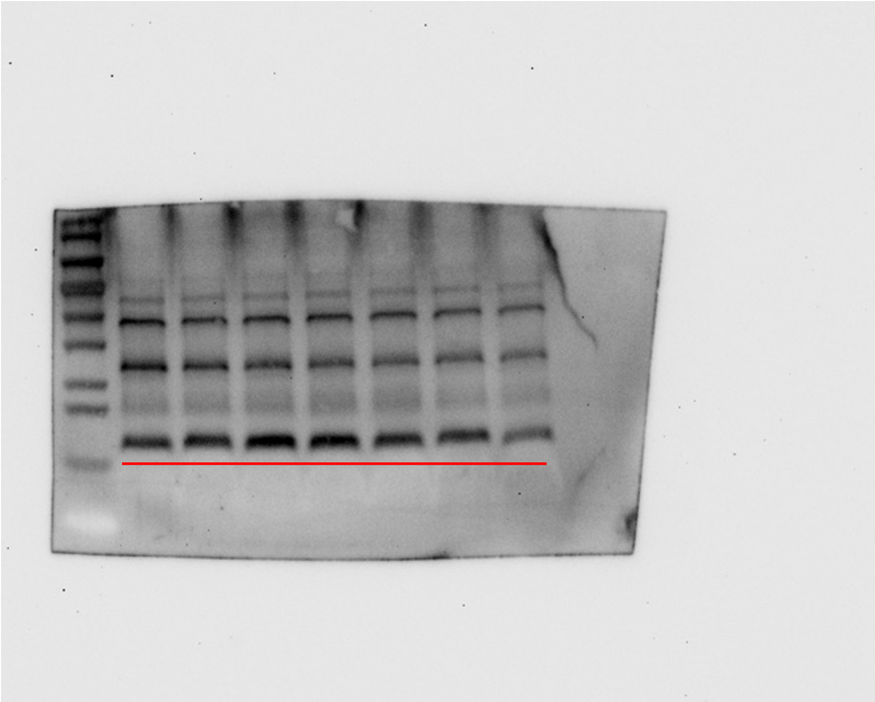

UBE2C-Halorotetin B-HepG2-3

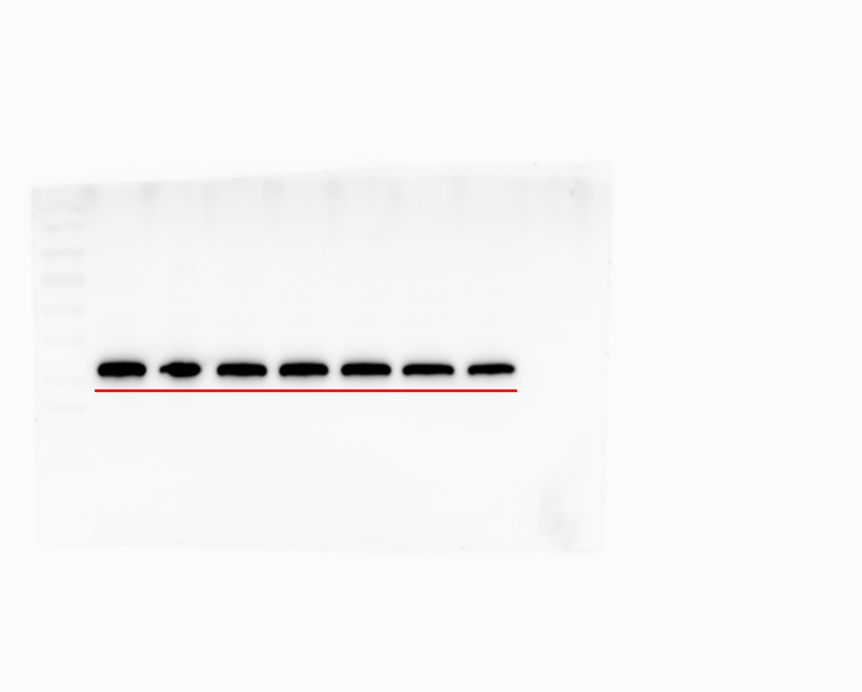

GAPDH-Halorotetin B-HepG2-3

CETSA experiments of HepG2 cell

Bel-7402 cell

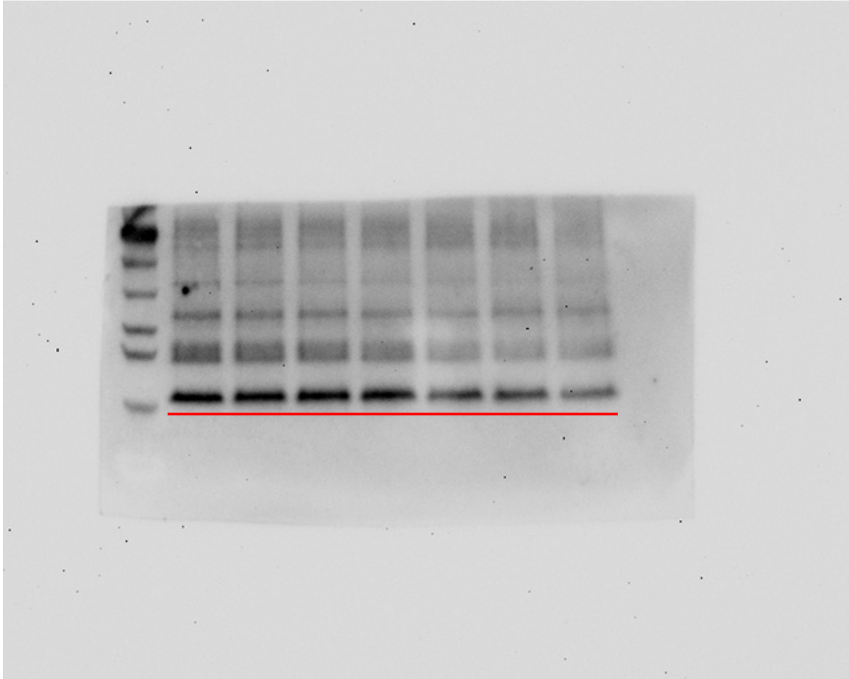

UBE2C-Control-Bel-7402-1

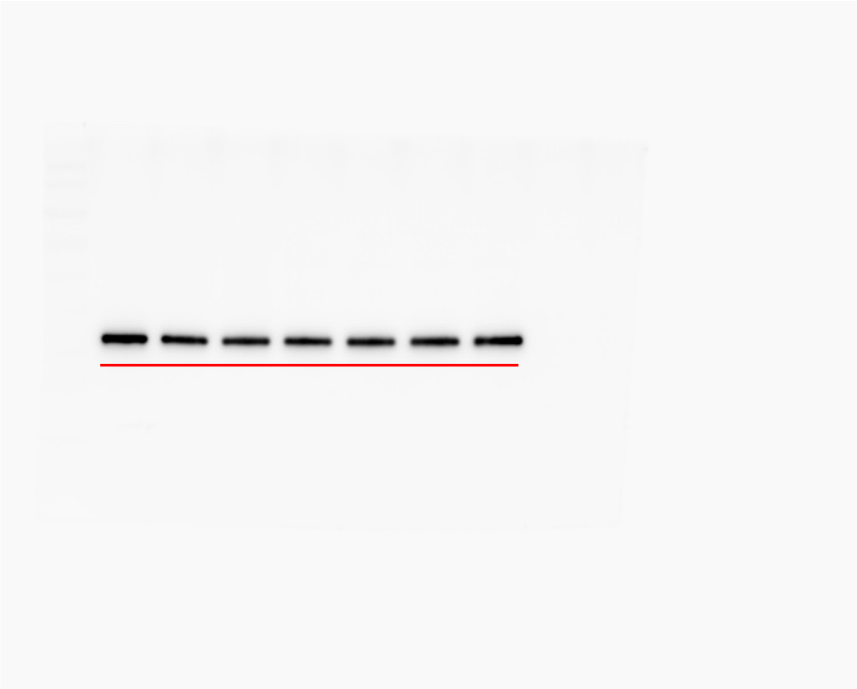

GAPDH-Control-Bel-7402-1

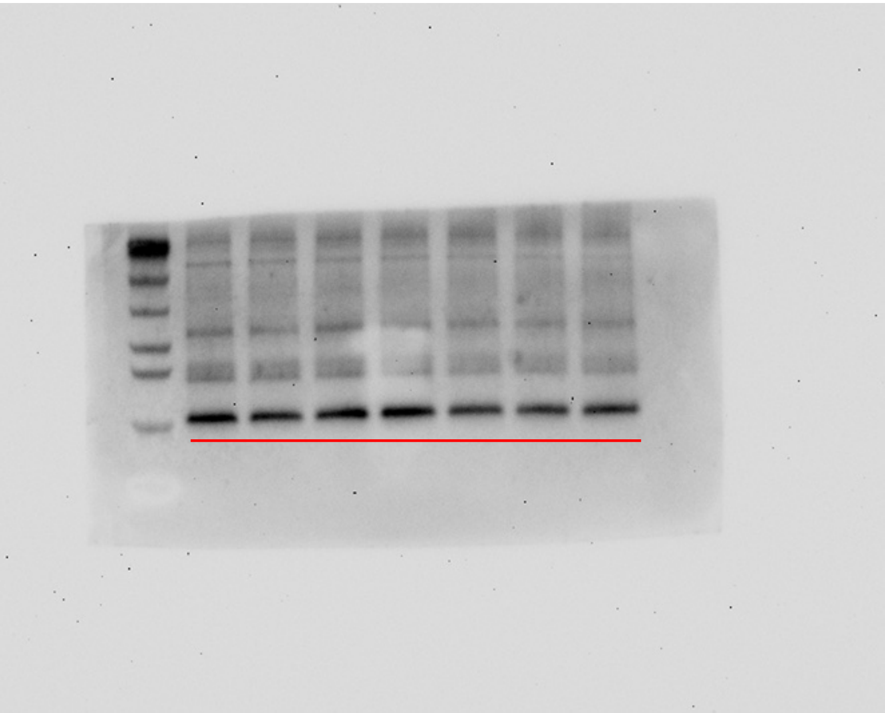

UBE2C-Halorotetin B-Bel-7402-1

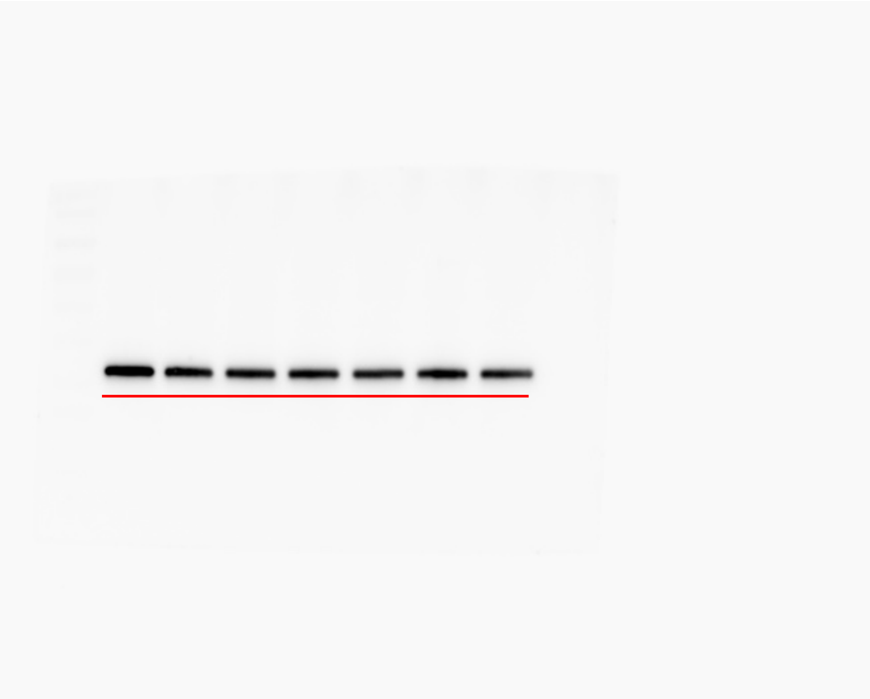

GAPDH-Halorotetin B-Bel-7402-1

The first independent replicate experiment (-1) was used to present in the manuscript, which related to Fig. 5d.

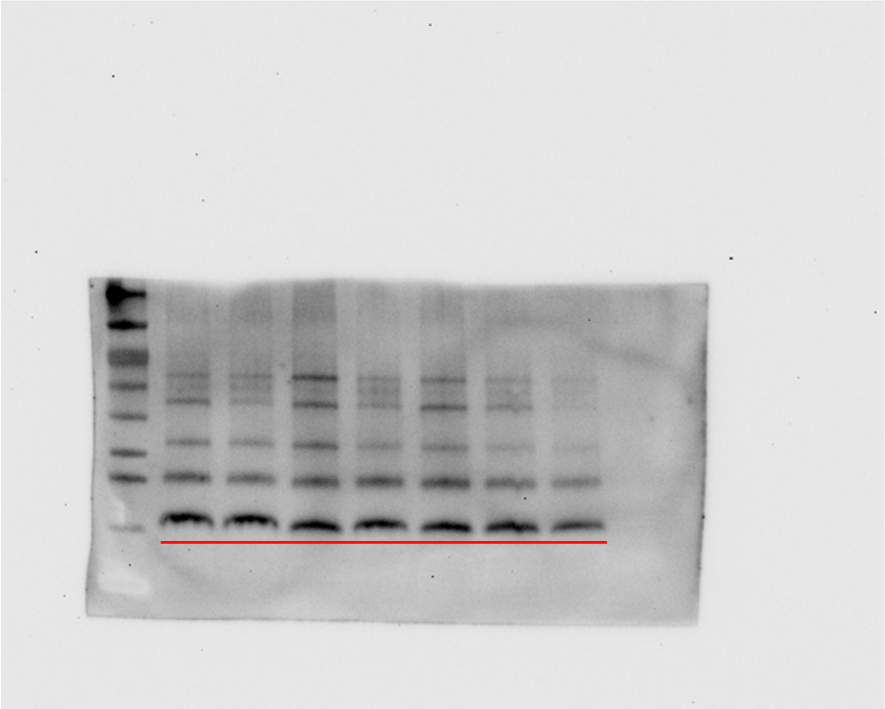

UBE2C-Control-Bel-7402-2

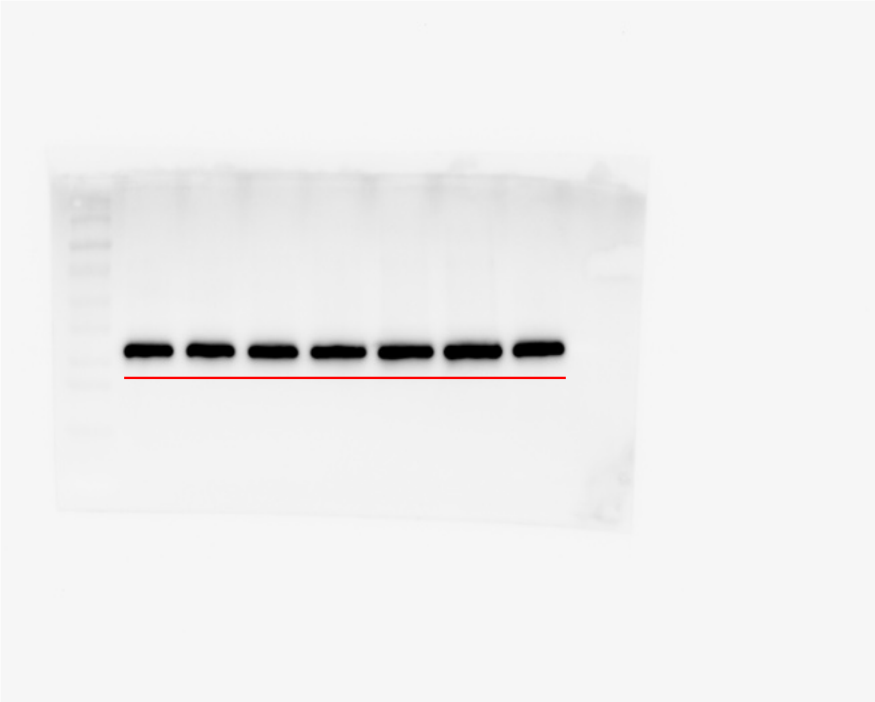

GAPDH-Control-Bel-7402-2

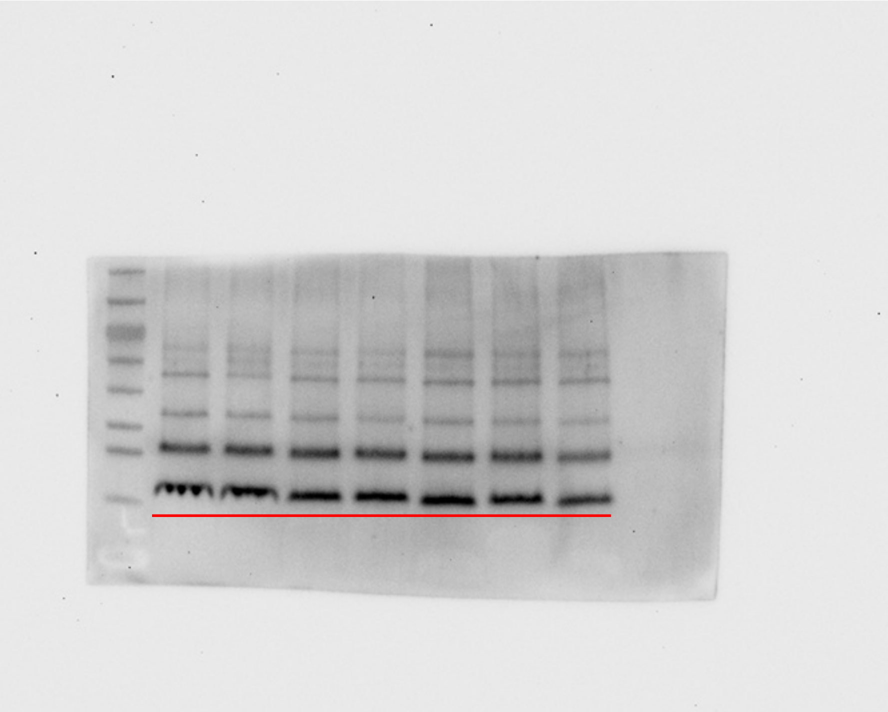

UBE2C-Halorotetin B-Bel-7402-2

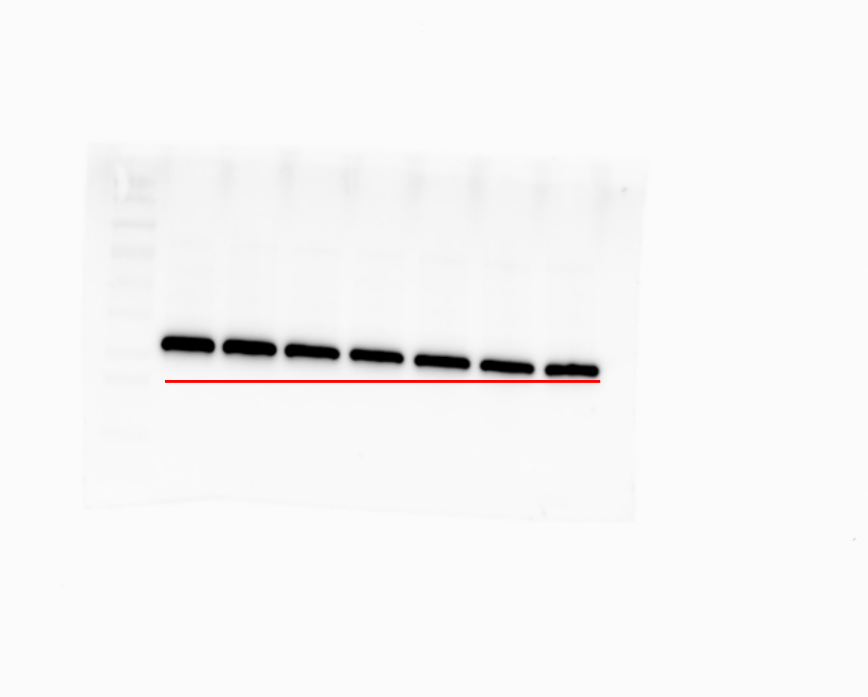

GAPDH-Halorotetin B-Bel-7402-2

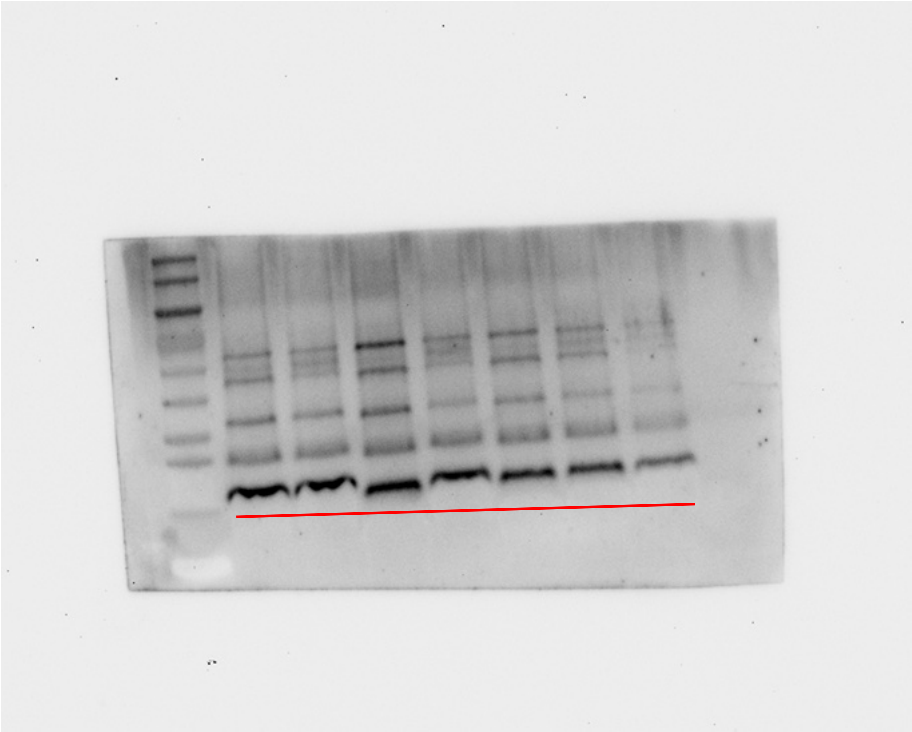

UBE2C-Control-Bel-7402-3

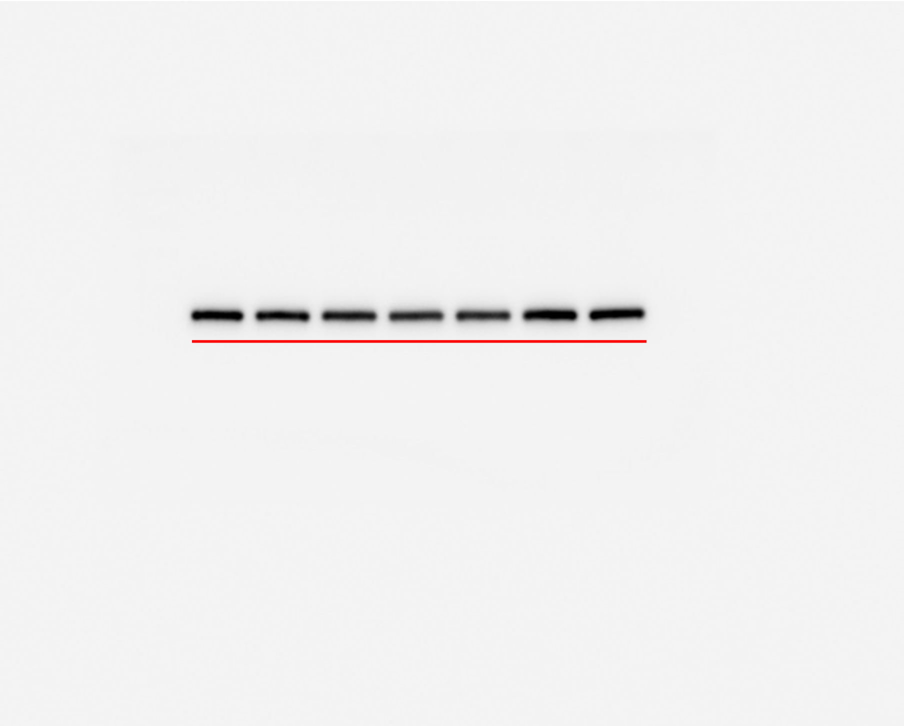

GAPDH-Control-Bel-7402-3

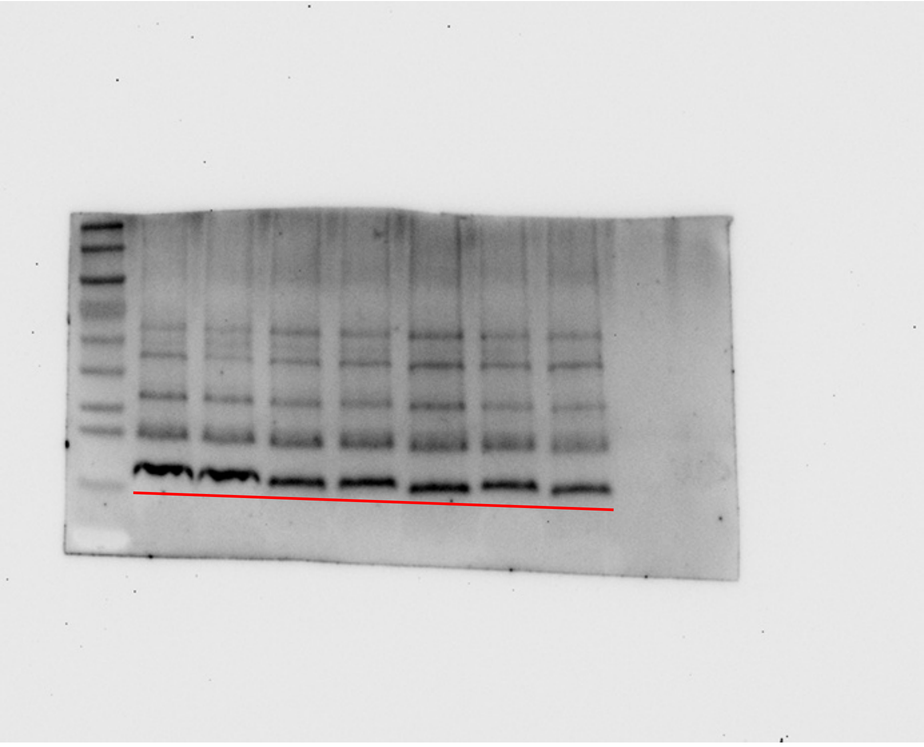

UBE2C-Halorotetin B-Bel-7402-3

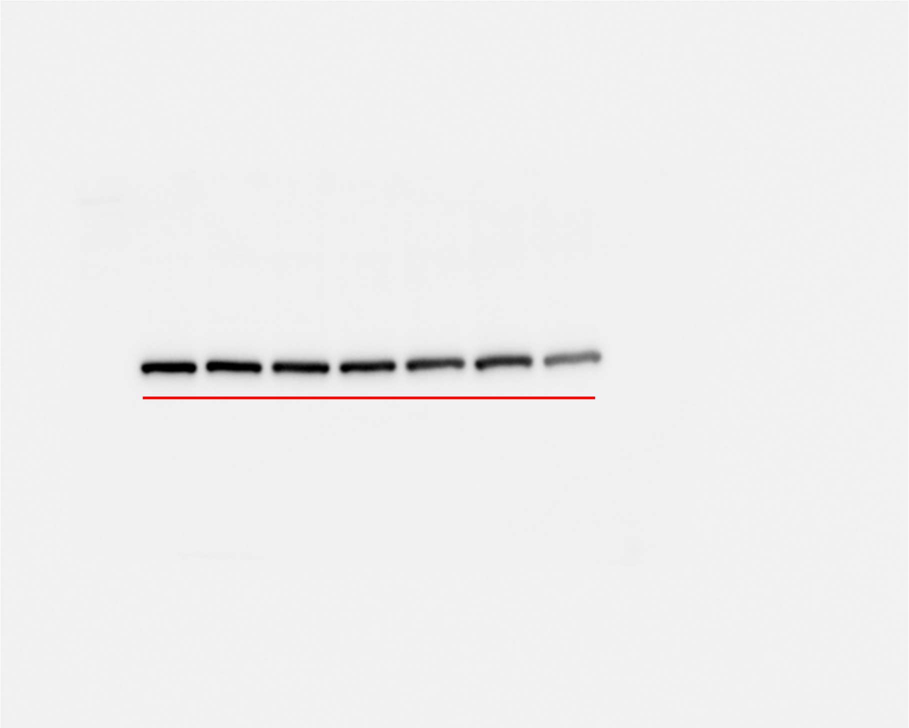

GAPDH-Halorotetin B-Bel-7402-3

CETSA experiments of Bel-7402 cell

Huh-7 cell

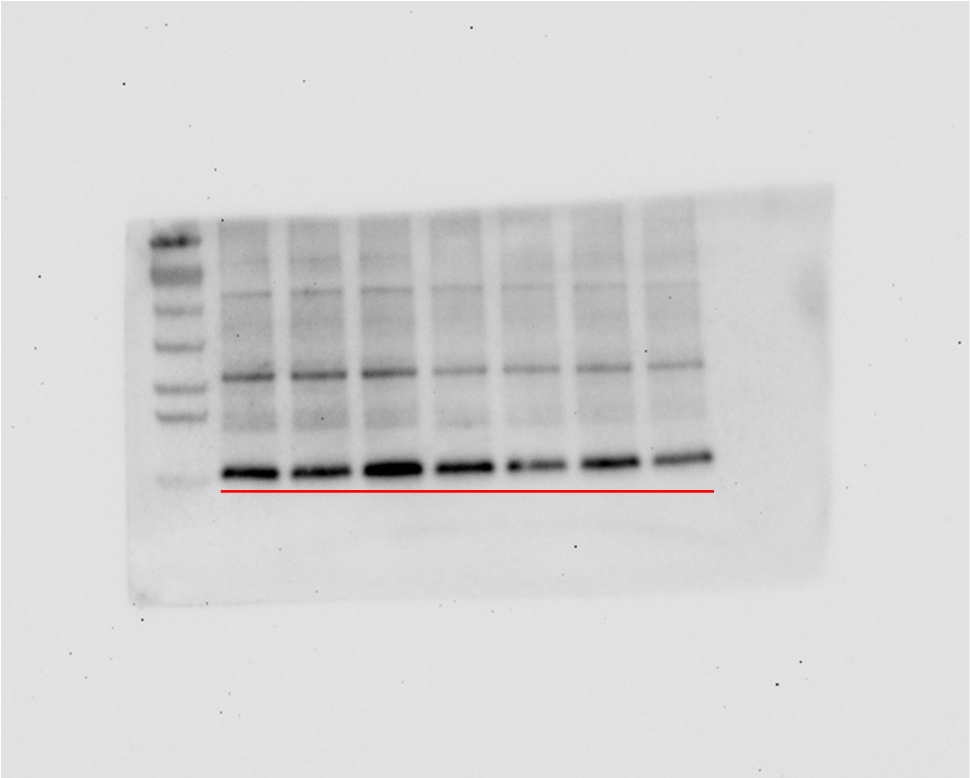

UBE2C-Control-Huh-7-1

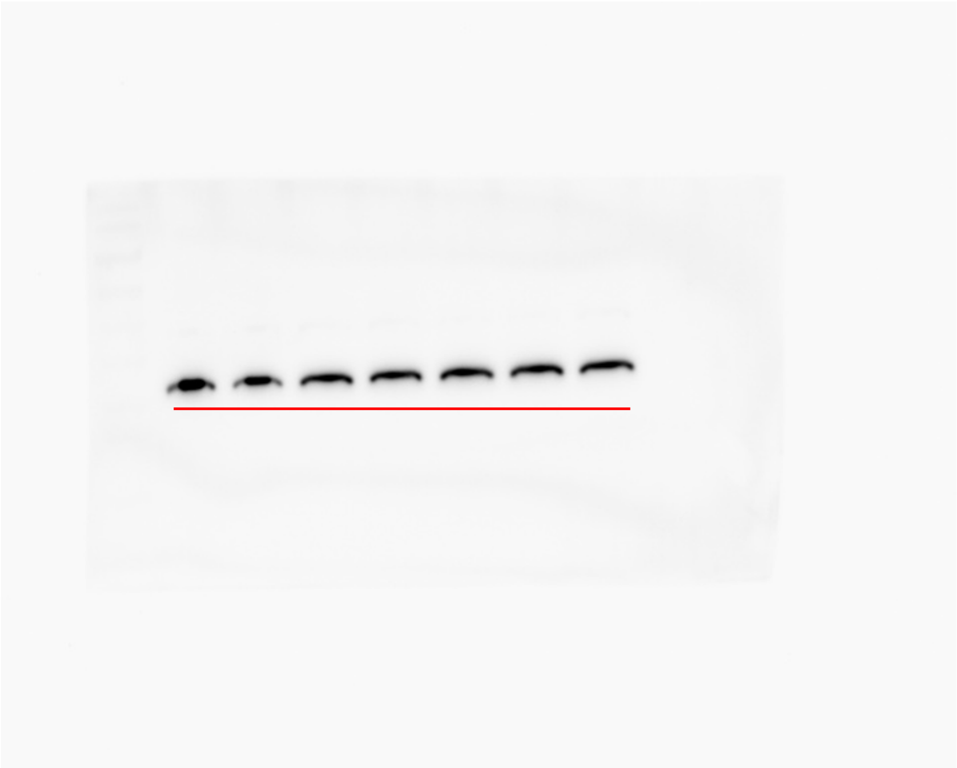

GAPDH-Control-Huh-7-1

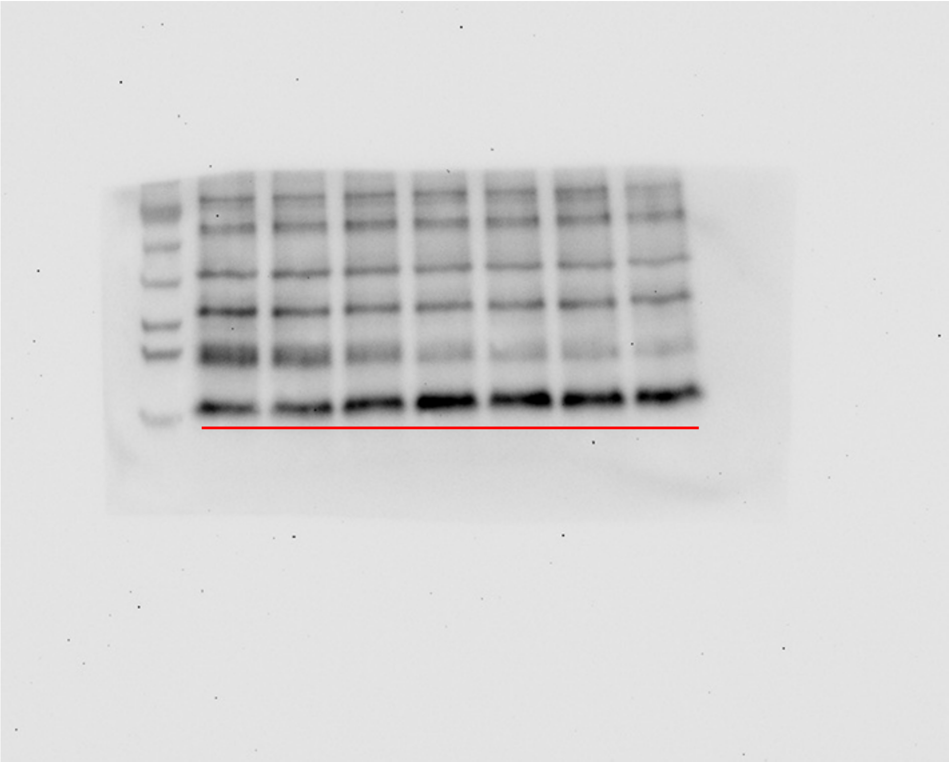

UBE2C-Halorotetin B-Huh-7-1

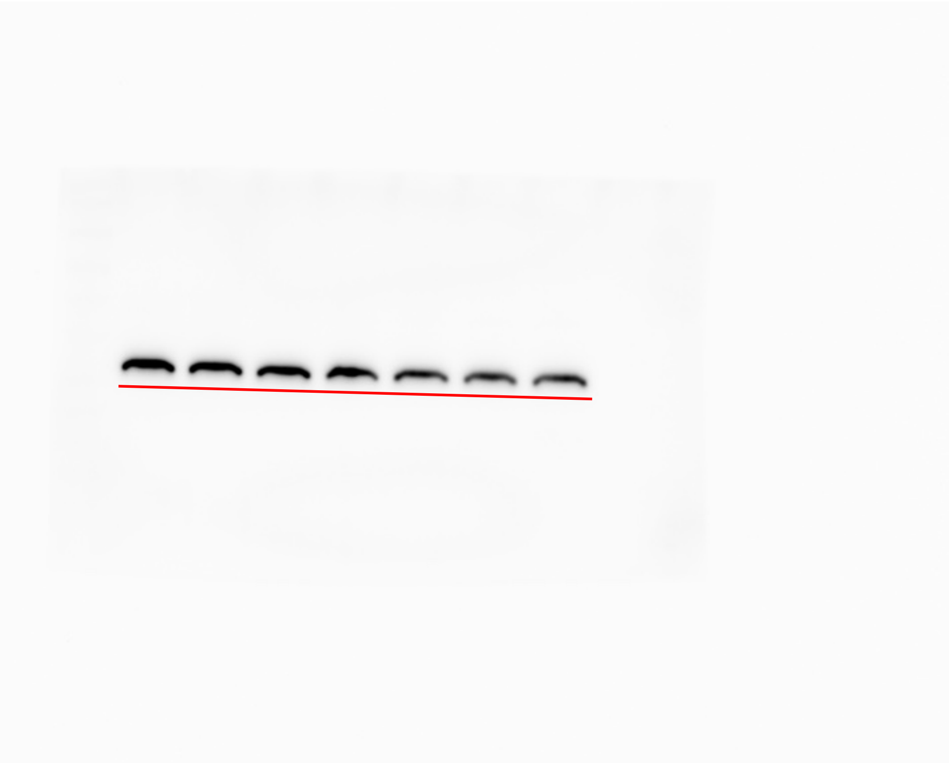

GAPDH-Halorotetin B-Huh-7-1

The first independent replicate experiment (-1) was used to present in the manuscript, which related to Fig. S6b.

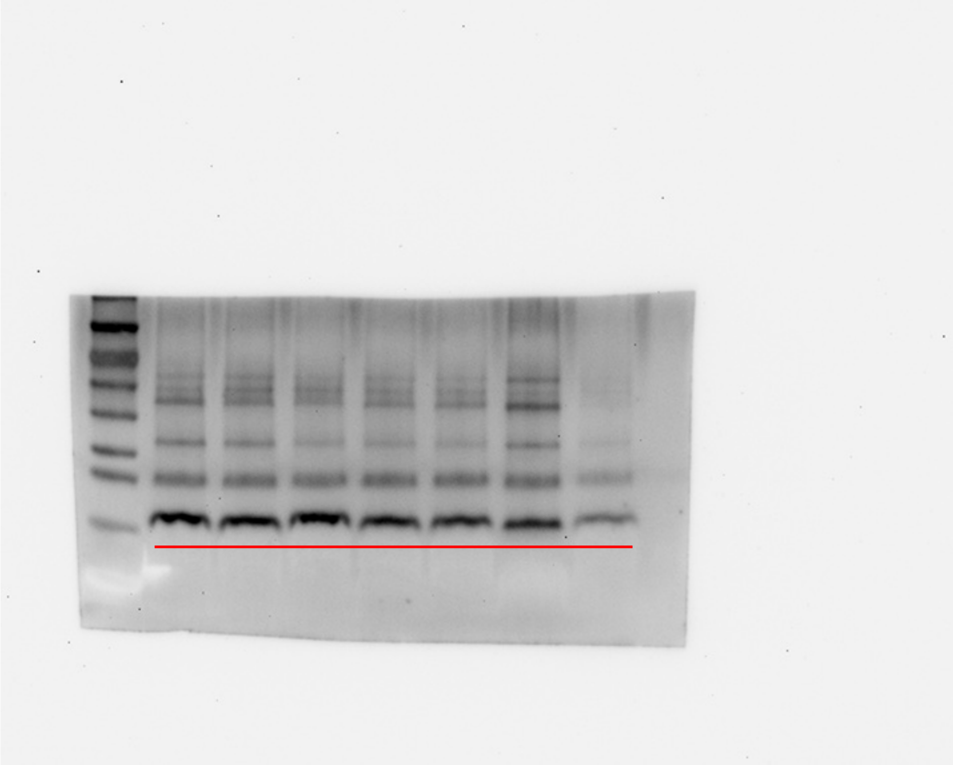

UBE2C-Control-Huh-7-2

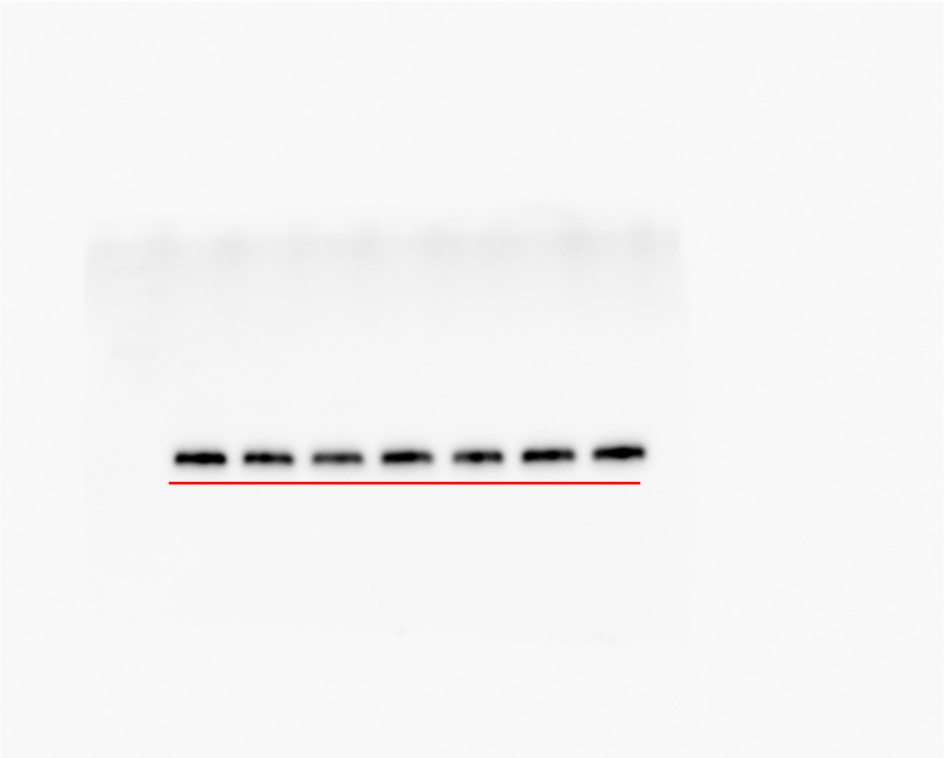

GAPDH-Control-Huh-7-2

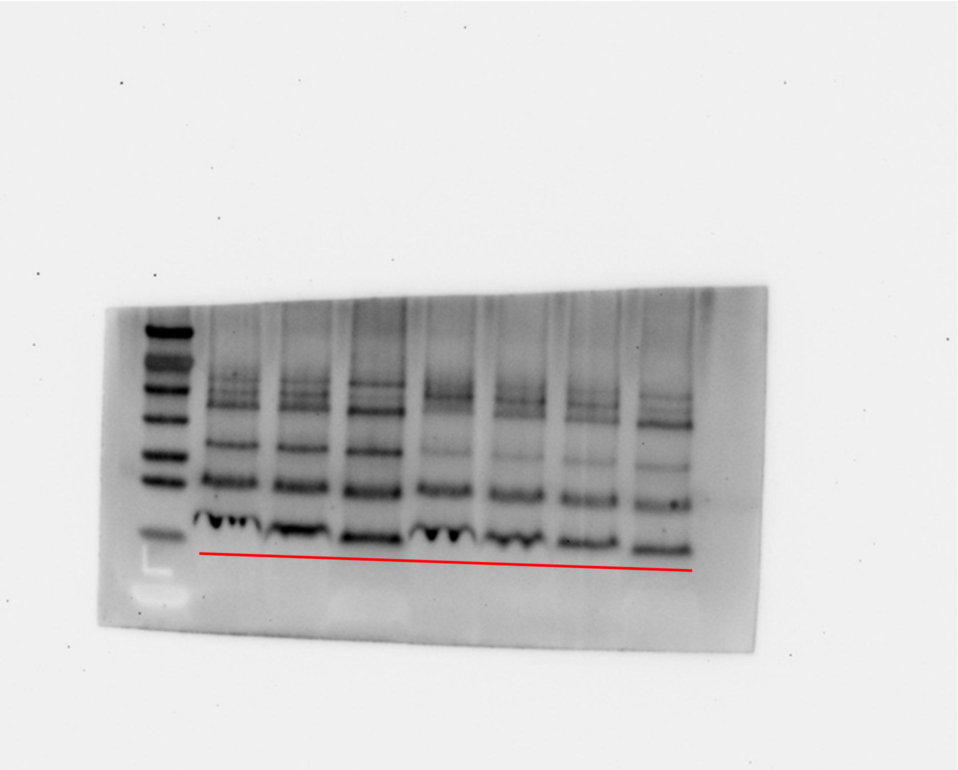

UBE2C-Halorotetin B-Huh-7-2

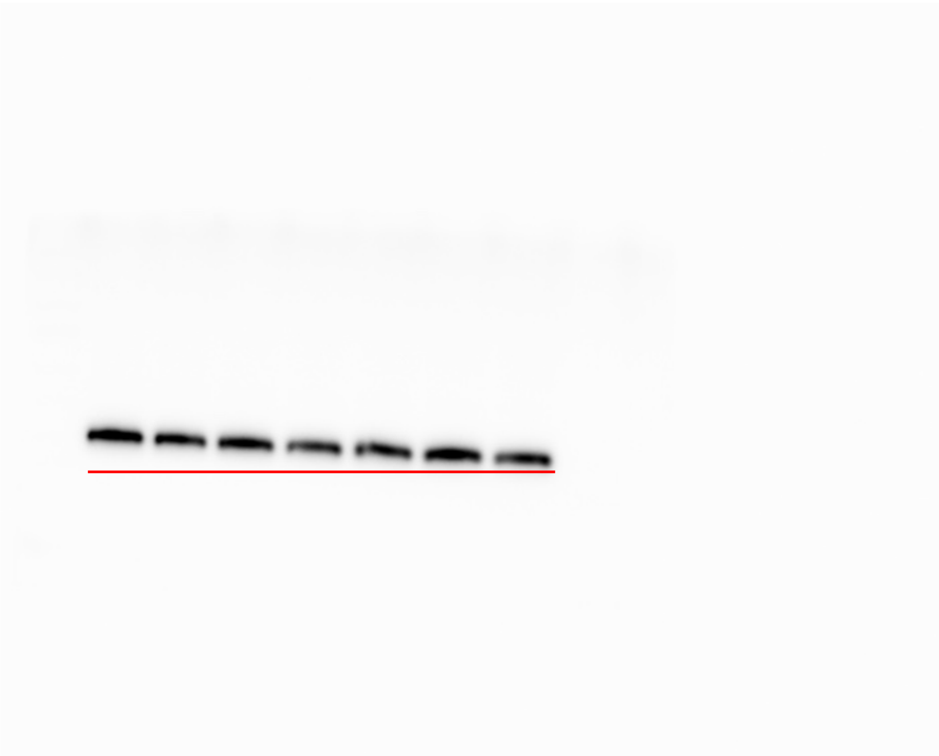

GAPDH-Halorotetin B-Huh-7-2

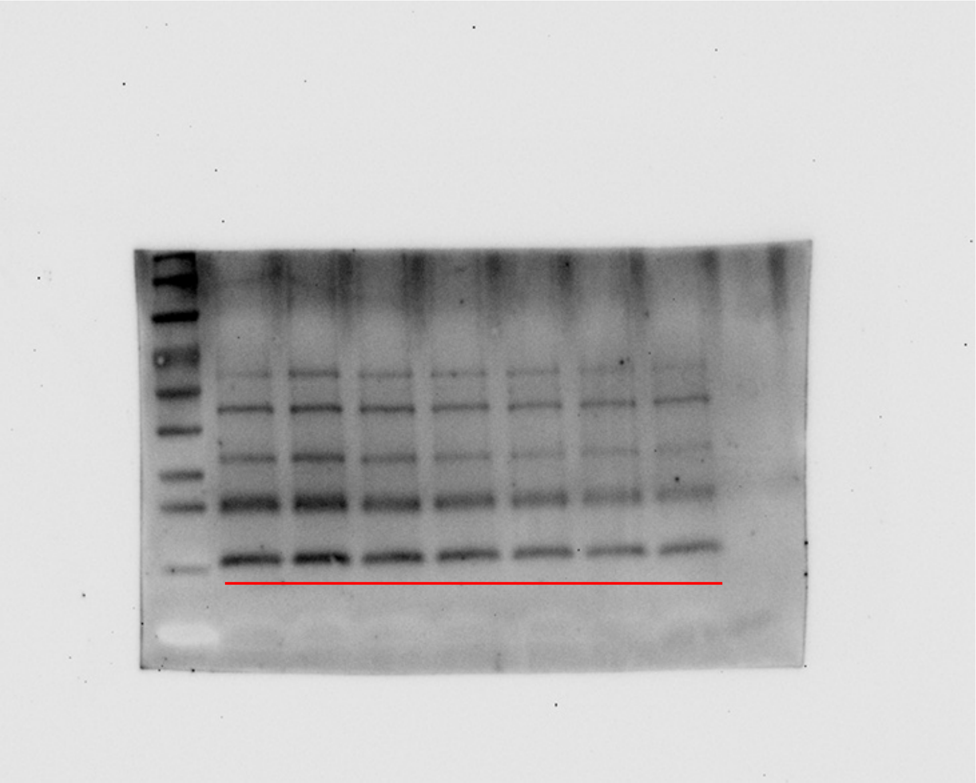

UBE2C-Control-Huh-7-3

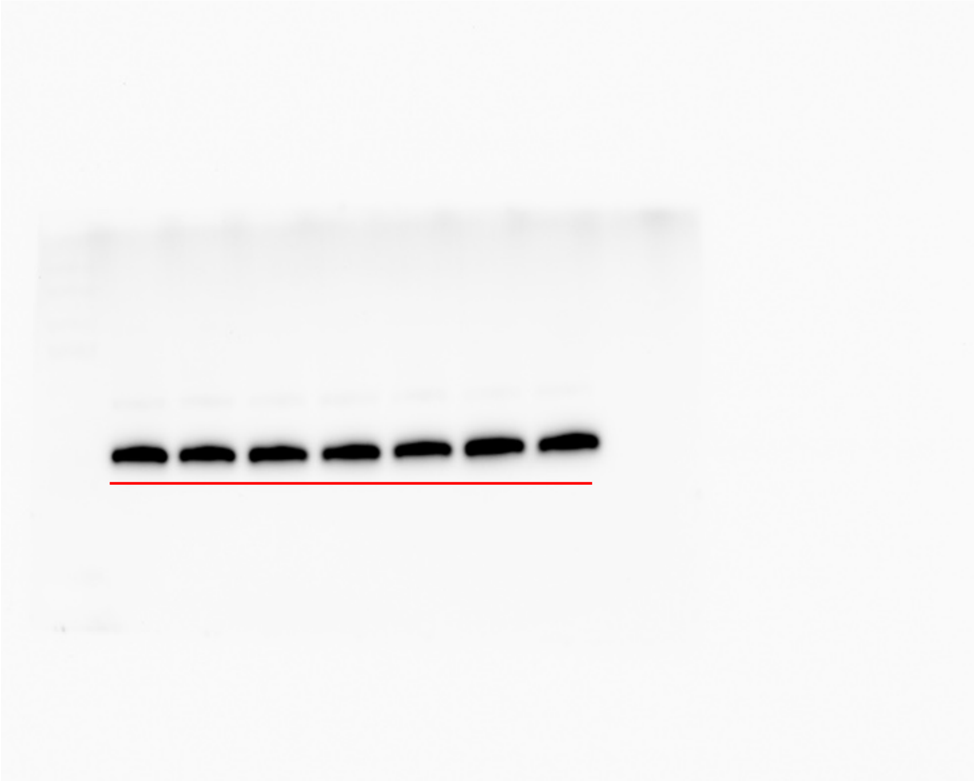

GAPDH-Control-Huh-7-3

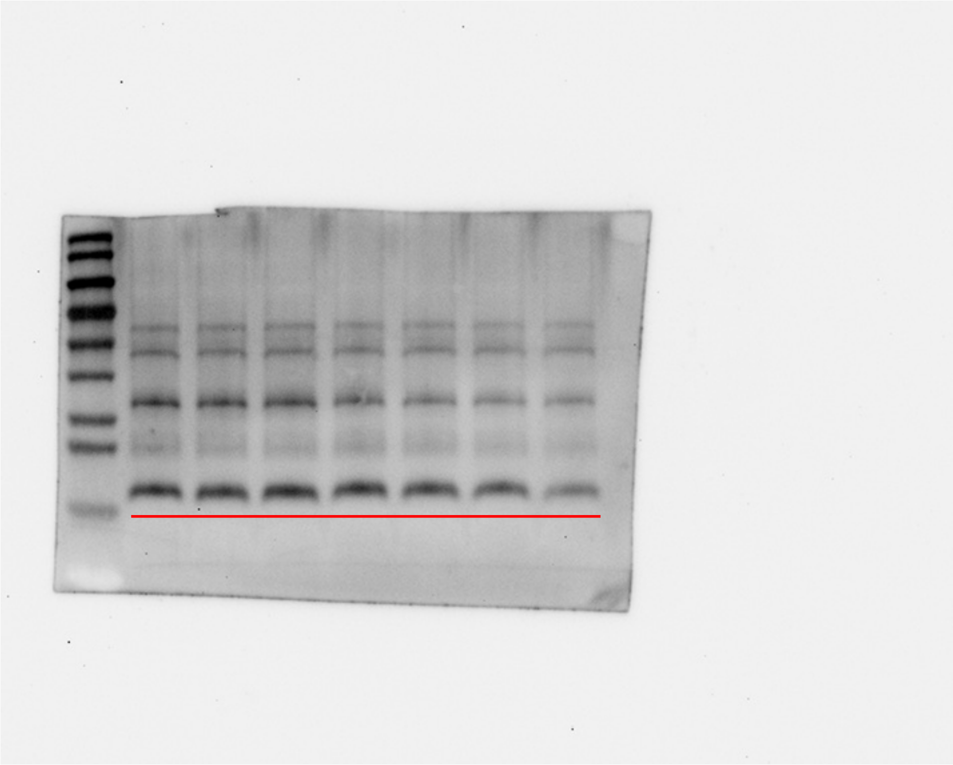

UBE2C-Halorotetin B-Huh-7-3

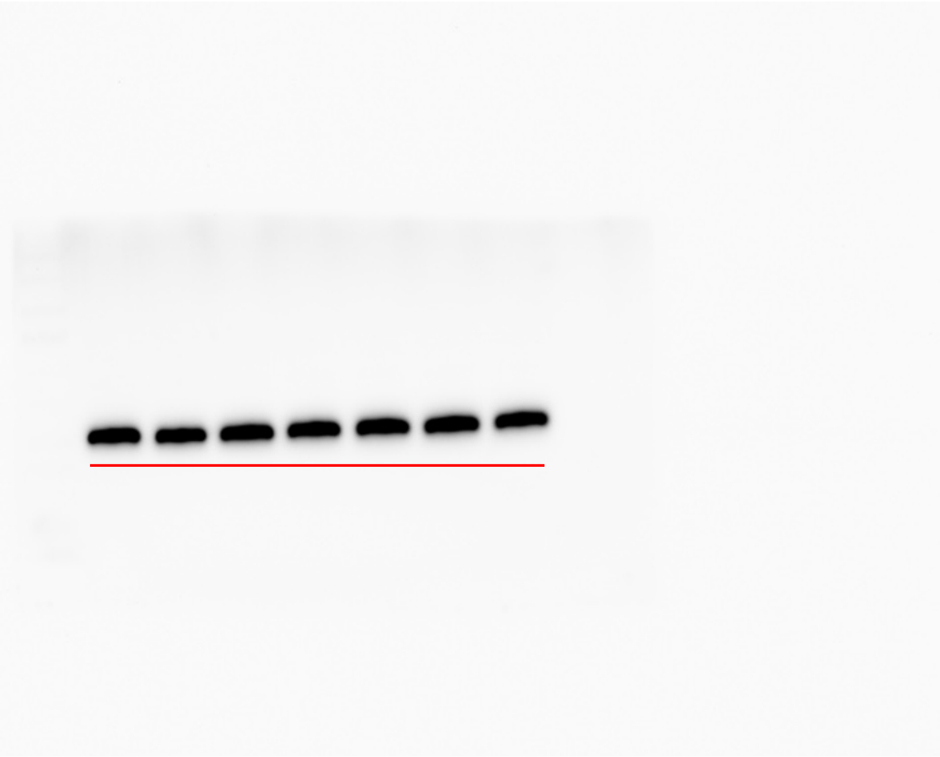

GAPDH-Halorotetin B-Huh-7-3

CETSA experiments of Huh-7 cell

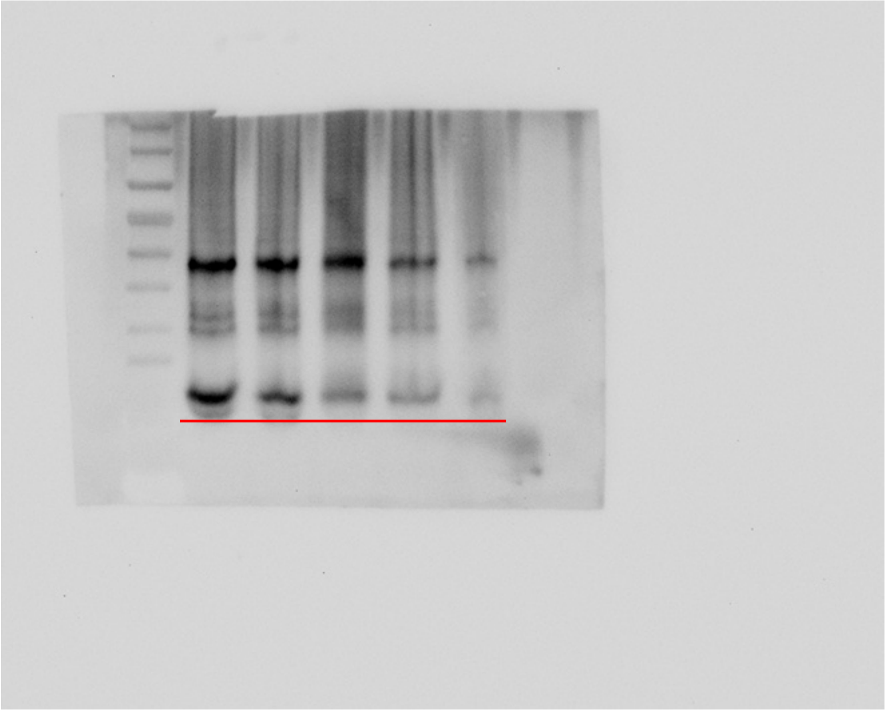

UBE2C-Control-1 (*In vivo*)

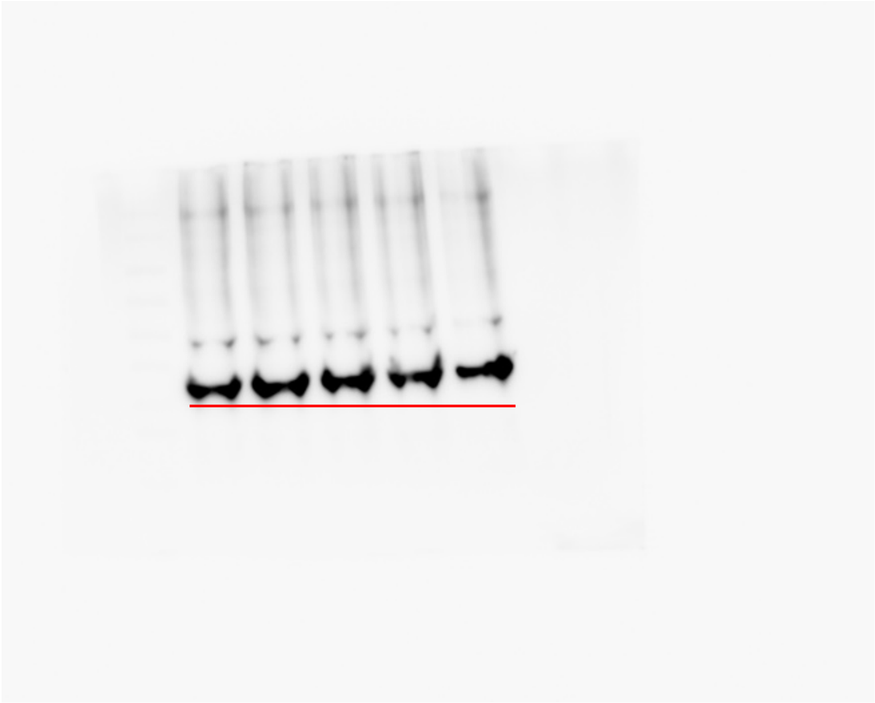

GAPDH-Control-1 (*In vivo*)

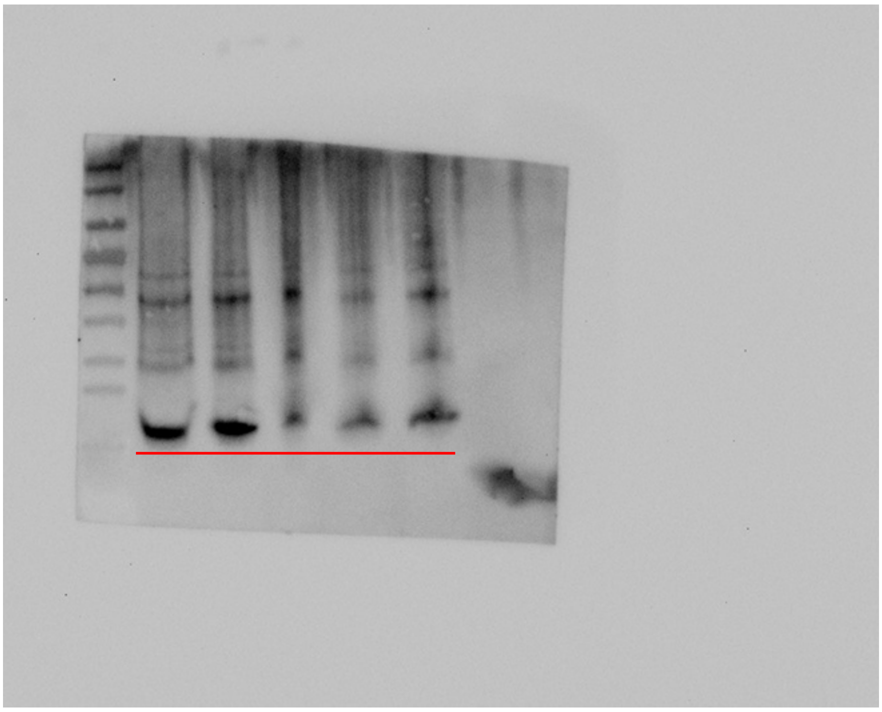

UBE2C-Halorotetin B-1 (*In vivo*)

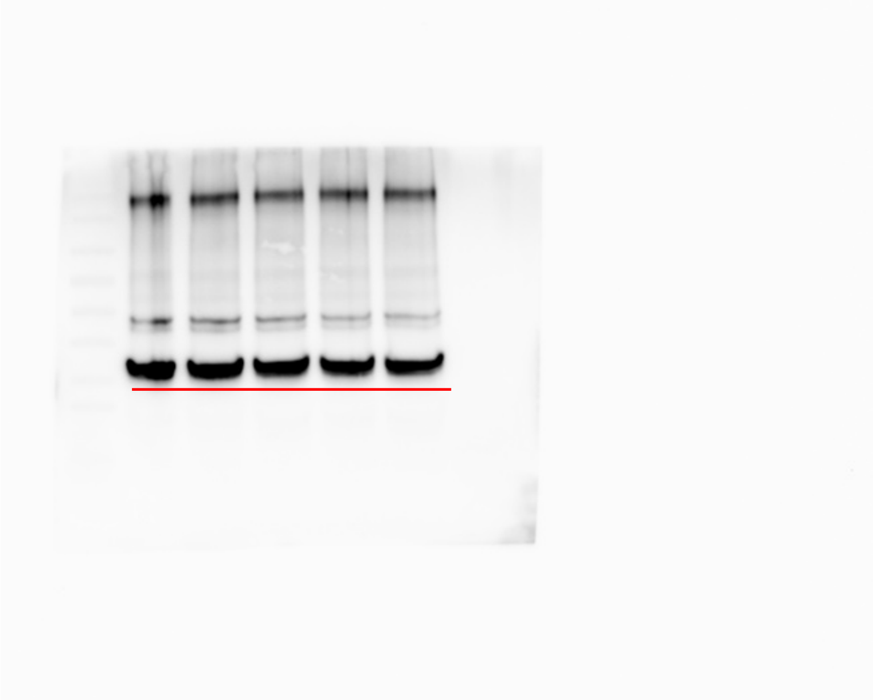

GAPDH-Halorotetin B-1 (*In vivo*)

The first independent replicate experiment (-1) was used to present in the manuscript, which related to Fig. S6c.

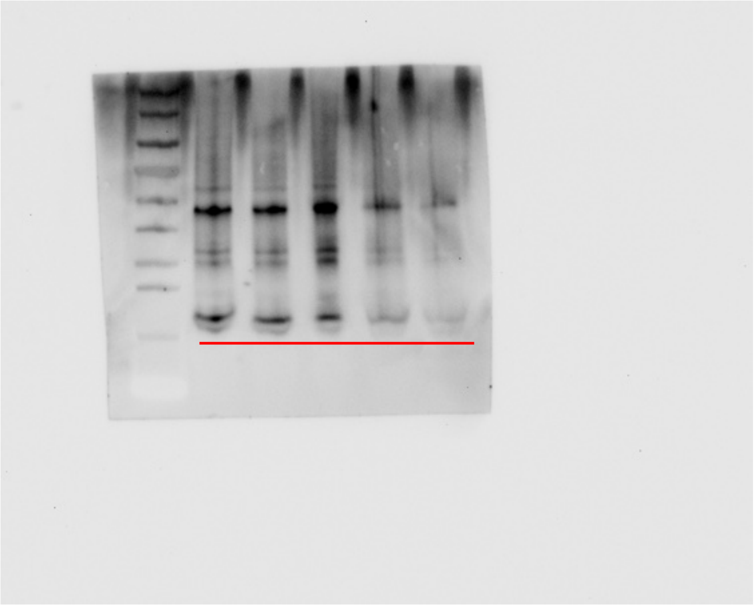

UBE2C-Control-2 (*In vivo*)

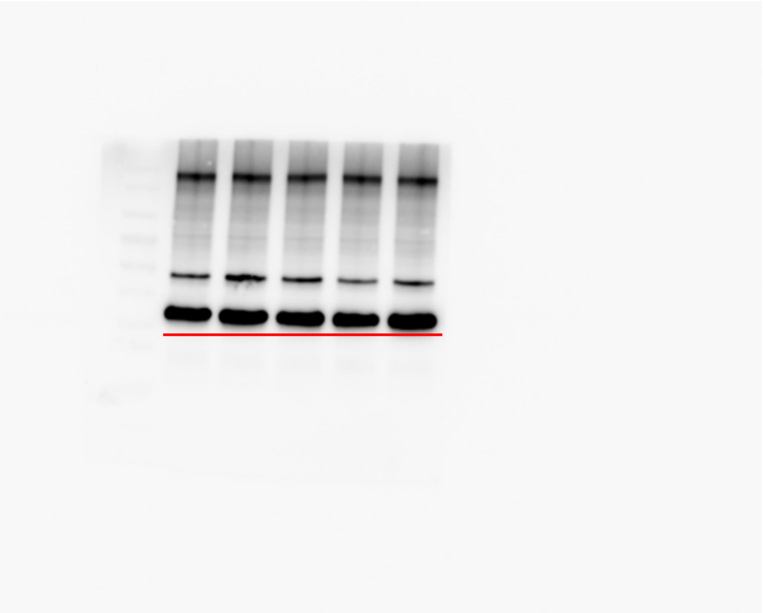

GAPDH-Control-2 (*In vivo*)

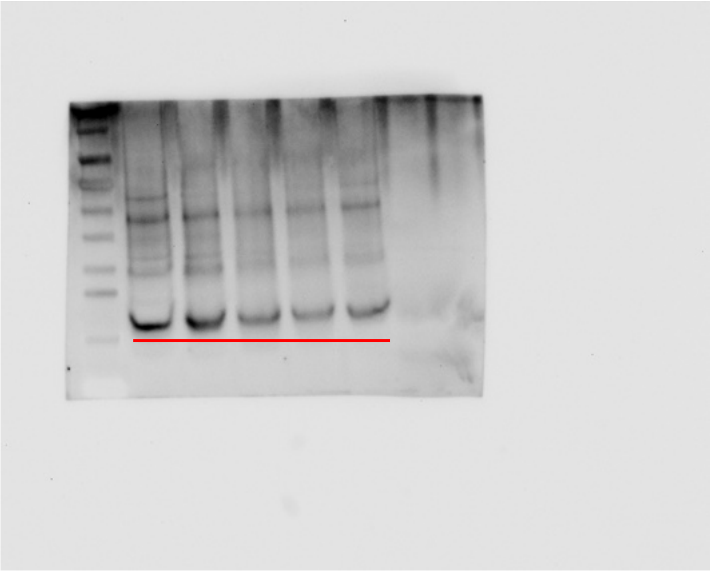

UBE2C-Halorotetin B-2 (*In vivo*)

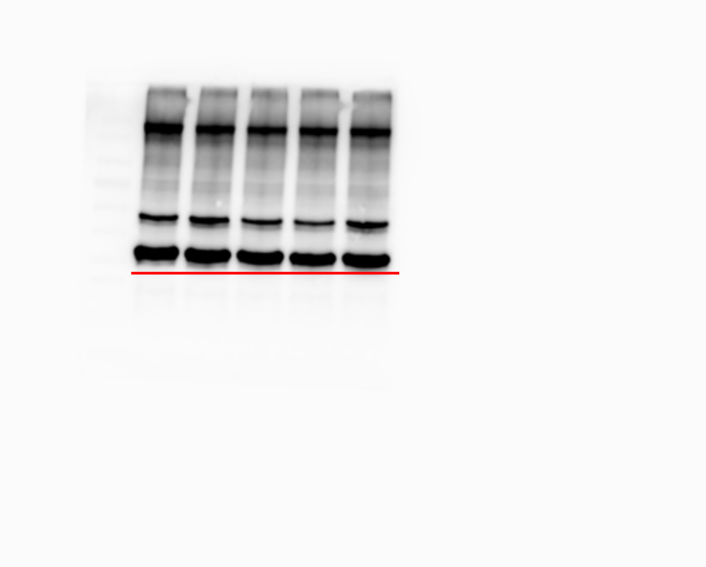

GAPDH-Halorotetin B-2 (*In vivo*)

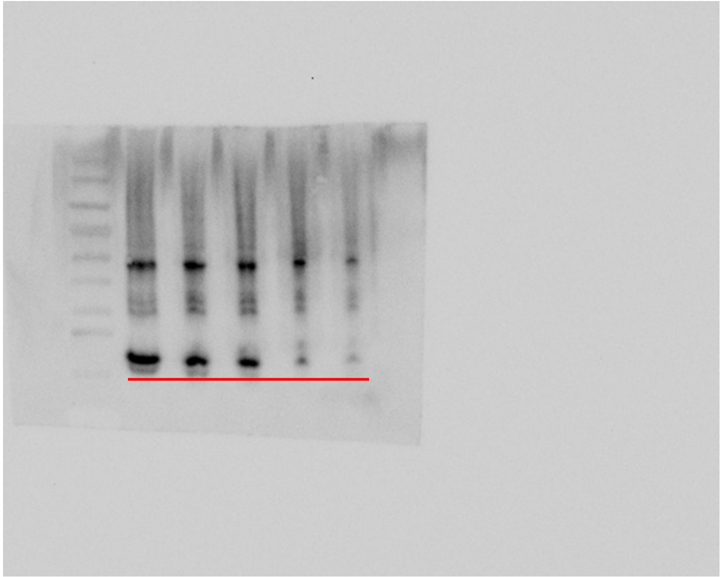

UBE2C-Control-3 (*In vivo*)

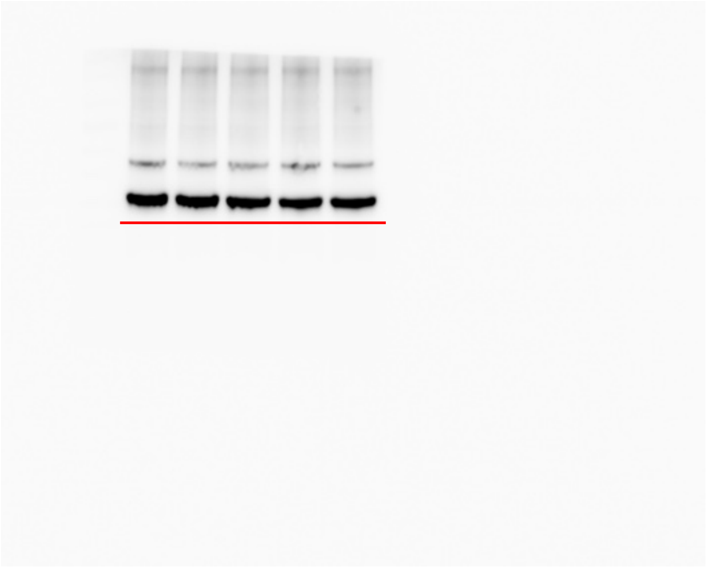

GAPDH-Control-3 (*In vivo*)

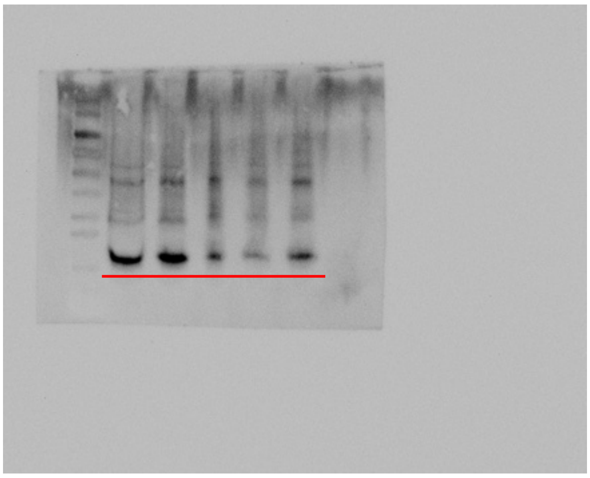

UBE2C-Halorotetin B-3 (*In vivo*)

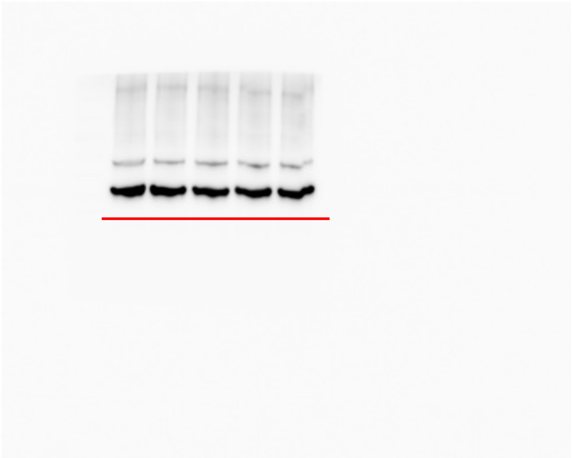

GAPDH-Halorotetin B-3 (*In vivo*)

Cellular Thermal Shift Assay (CETSA), related to Fig. 5d, S6b, and S6c.

Enzymatic activity of UBE2C

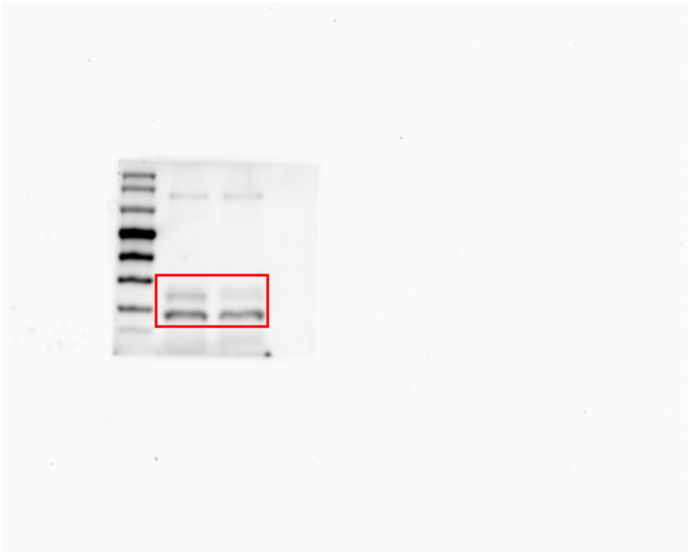

Enzymatic activity detection of UBE2C-1

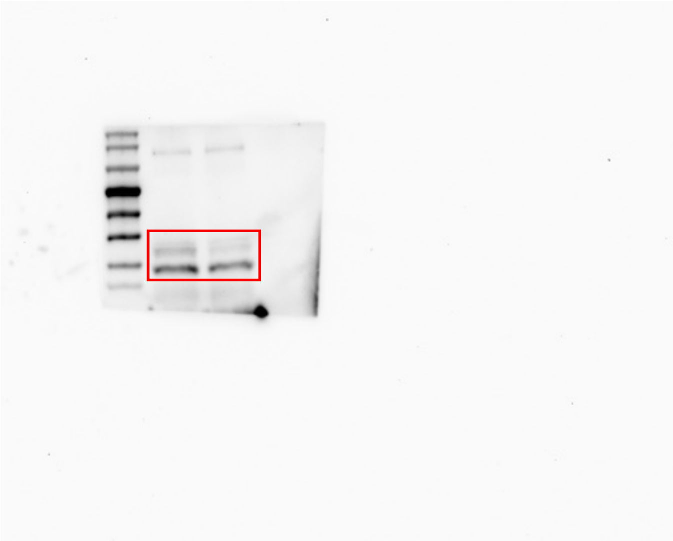

Enzymatic activity detection of UBE2C-2

The second independent replicate experiment (-2) was used to present in the manuscript, which related to Fig. 5g.

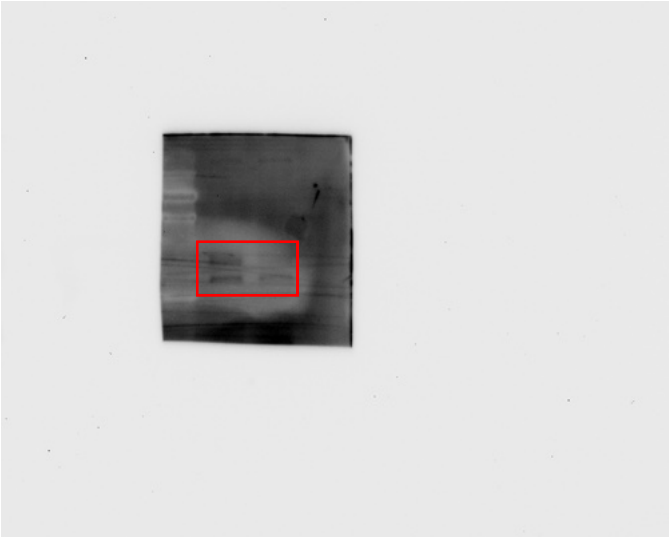

Enzymatic activity detection of UBE2C-3

Enzymatic activity detection of UBE2C, related to Fig. 5g.

CETSA-UBE2C-Amino acid (Ser 51) mutation

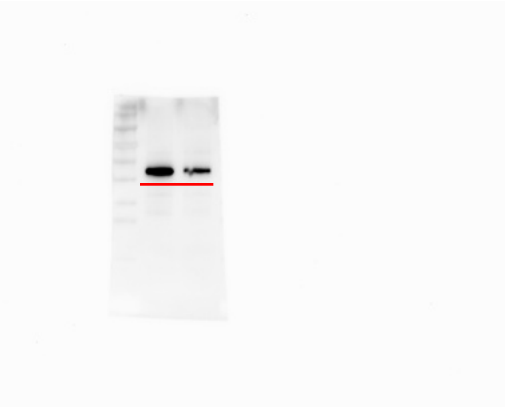

UBE2C (WT)-UBE2C (Ser51 mutation)-1

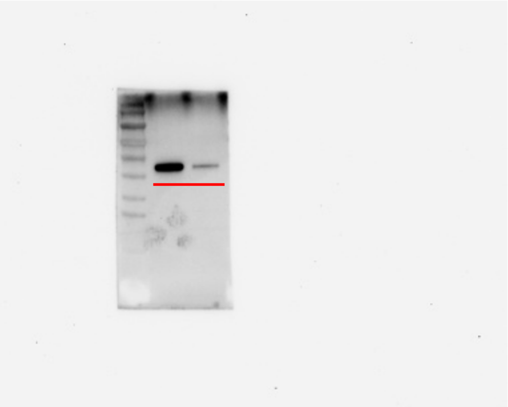

UBE2C (WT)-UBE2C (Ser51 mutation)-1 (77 °C for 10 min)

The first independent replicate experiment (-1) was used to present in the manuscript, which related to Fig. 5i.

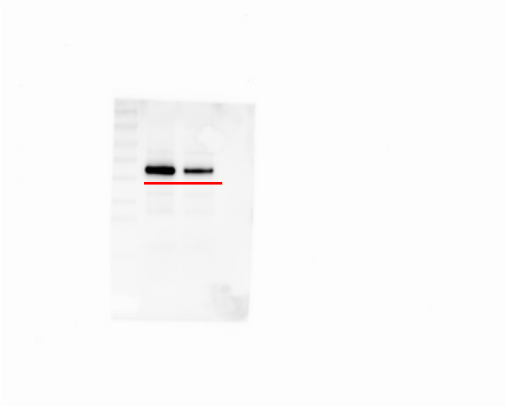

UBE2C (WT)-UBE2C (Ser51 mutation)-2

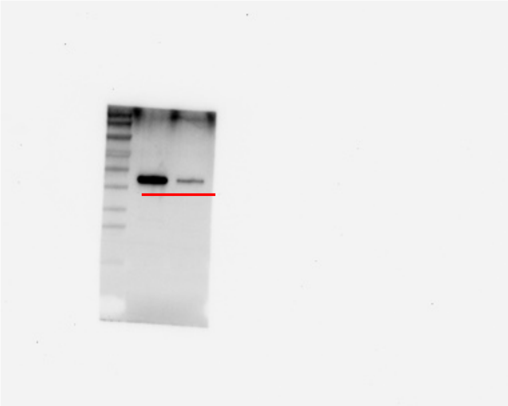

UBE2C (WT)-UBE2C (Ser51 mutation)-2 (77 °C for 10 min)

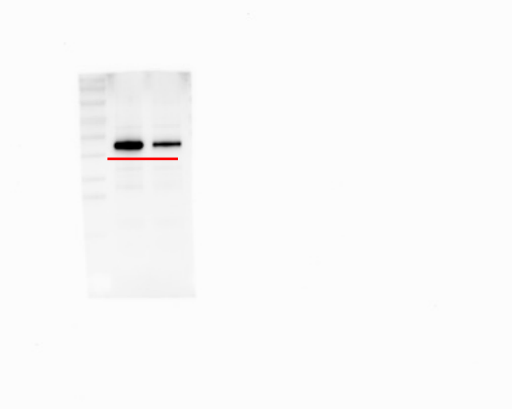

UBE2C (WT)-UBE2C (Ser51 mutation)-3

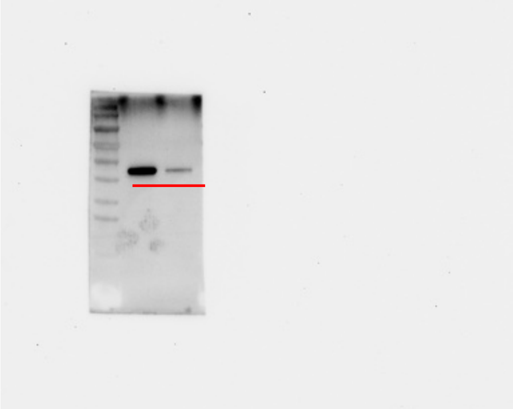

UBE2C (WT)-UBE2C (Ser51 mutation)-3 (77 °C for 10 min)

CETSA of amino acid mutation experiments, related to Fig. 5i.

UBE2C-RNAi

HepG2 cell

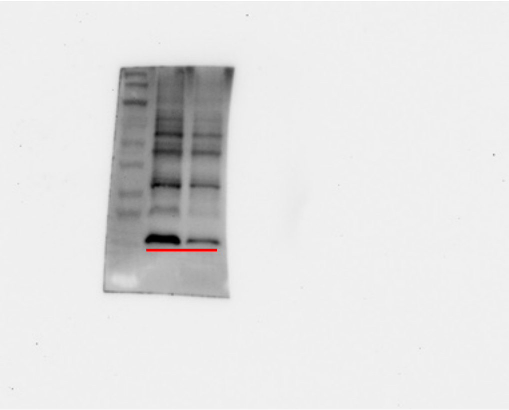

HepG2-UBE2C-siControl-siUBE2C-1

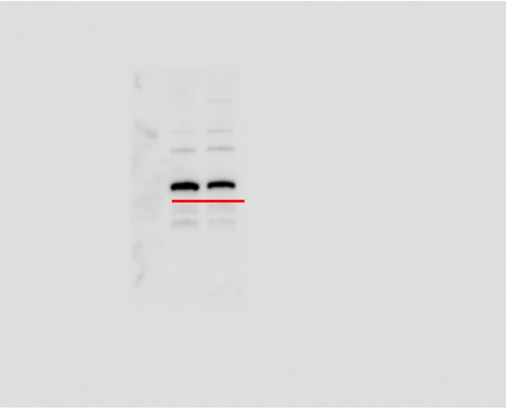

HepG2-GAPDH-siControl-siUBE2C-1

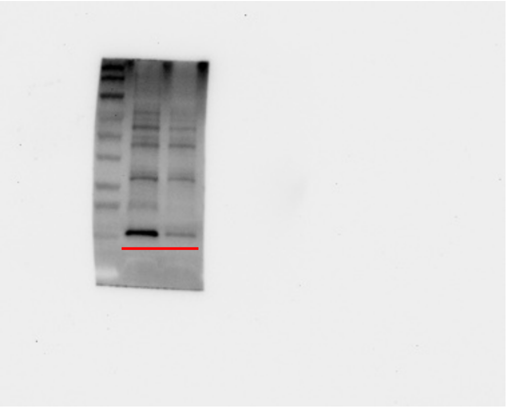

HepG2-UBE2C-siControl-siUBE2C-2

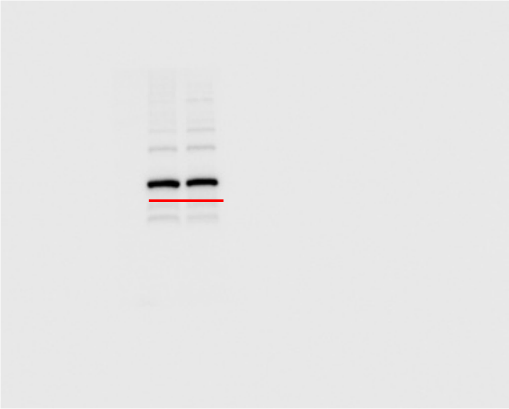

HepG2-GAPDH-siControl-siUBE2C-2

The second independent replicate experiment (-2) was used to present in the manuscript, which related to Fig. 6d.

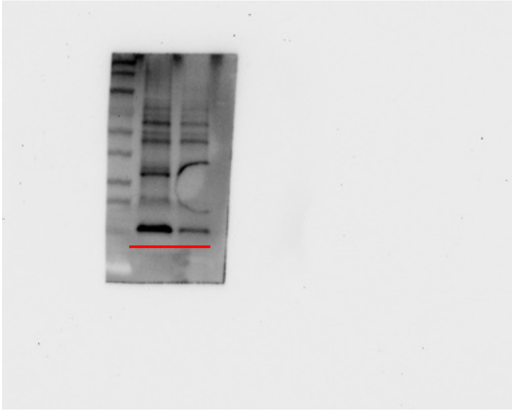

HepG2-UBE2C-siControl-siUBE2C-3

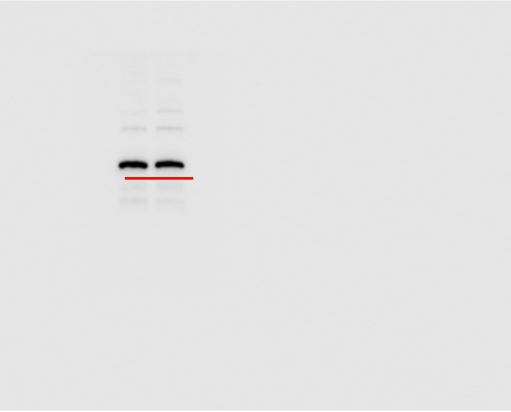

HepG2-GAPDH-siControl-siUBE2C-3

Knockdown of UBE2C of HepG2 cell

Bel-7402 cell

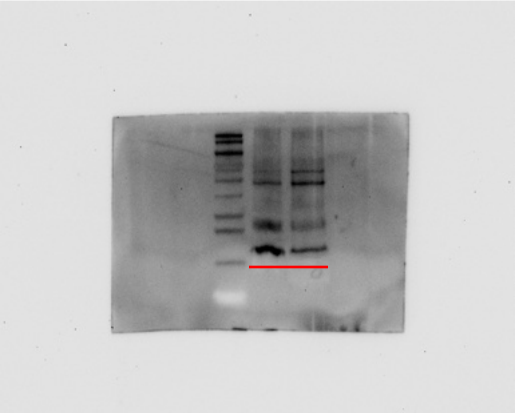

Bel-7402-UBE2C-siControl-siUBE2C-1

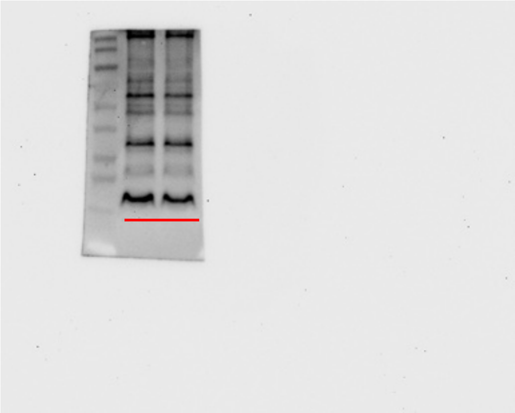

Bel-7402-UBE2C-siControl-siUBE2C-2

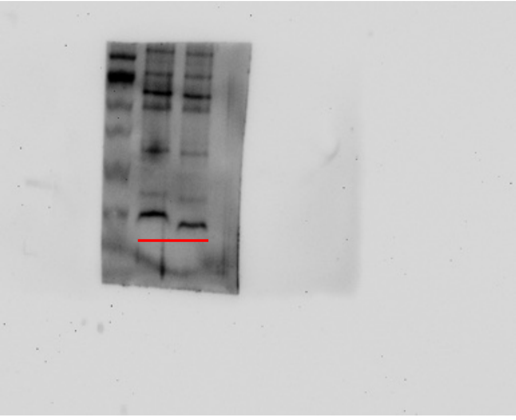

Bel-7402-UBE2C-siControl-siUBE2C-3

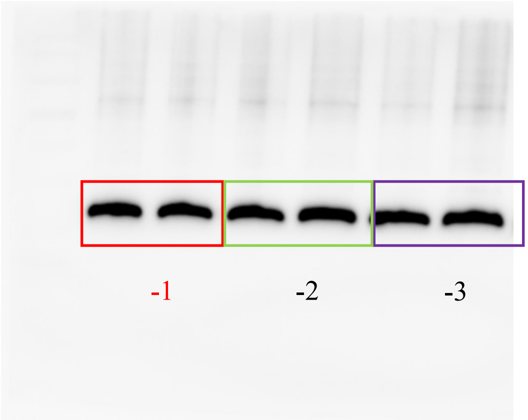

Bel-7402-GAPDH-siControl-siUBE2C-1,2,3

The first independent replicate experiment (-1) was used to present in the manuscript, which related to Fig. 6d.

Knockdown of UBE2C of Bel-7402 cell

Huh-7 cell

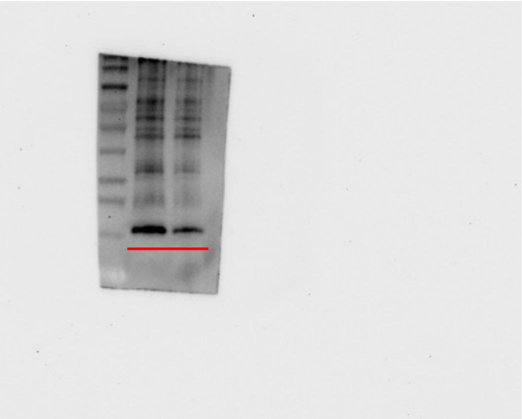

Huh-7-UBE2C-siControl-siUBE2C-1

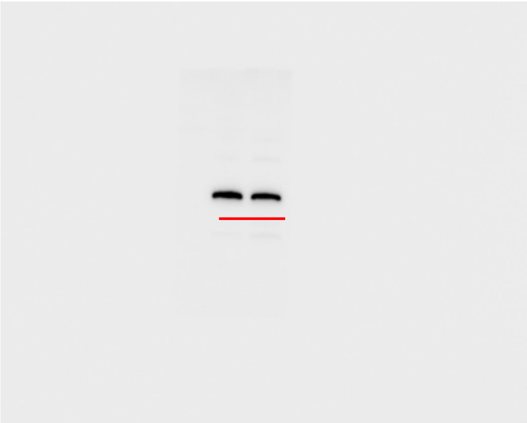

Huh-7-GAPDH-siControl-siUBE2C-1

The first independent replicate experiment (-1) was used to present in the manuscript, which related to Fig. S7a.

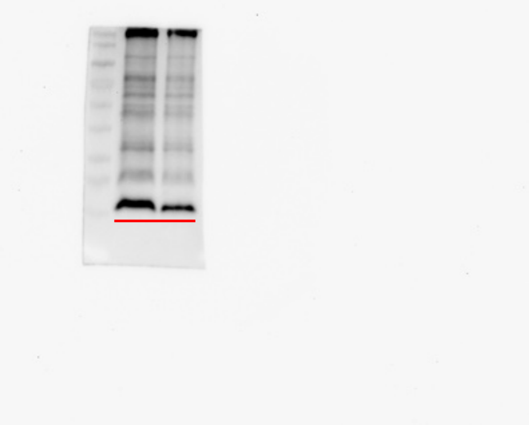

Huh-7-UBE2C-siControl-siUBE2C-2

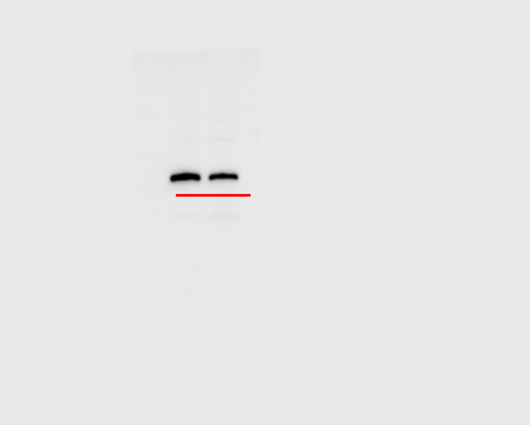

Huh-7-GAPDH-siControl-siUBE2C-2

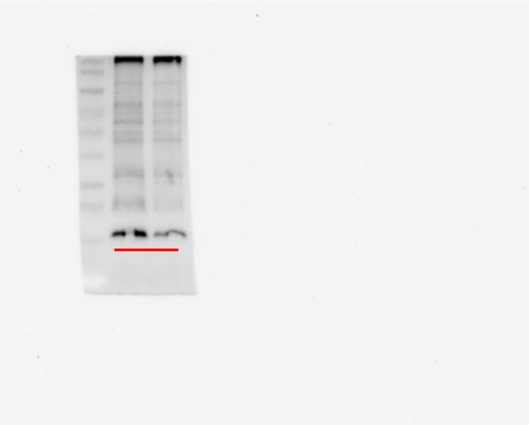

Huh-7-UBE2C-siControl-siUBE2C-3

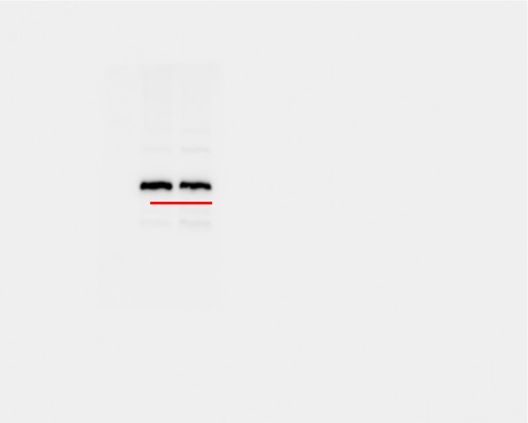

Huh-7-GAPDH-siControl-siUBE2C-3

Knockdown of UBE2C of Huh-7 cell

Detection of UBE2C expression after RNAi, related to Fig. 6d and S7a.

Cyclin B1 Expression

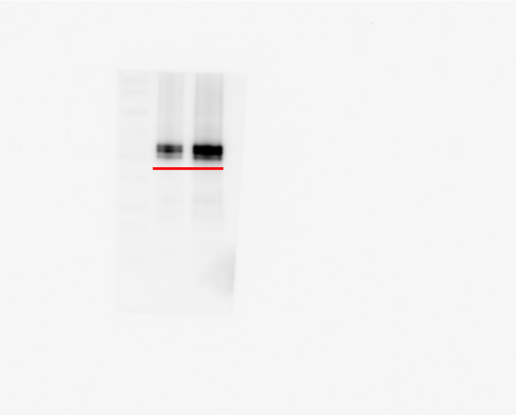

Cyclin B1-Control-H.B-1

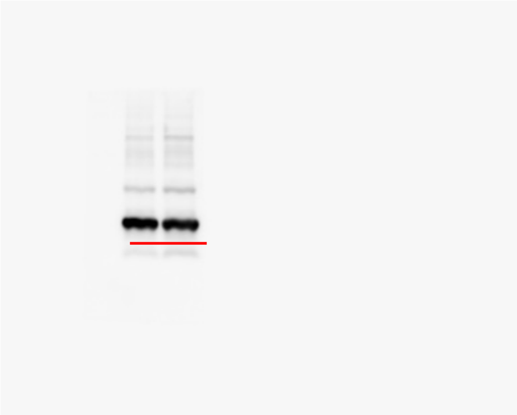

GAPDH-Control-H.B-1

The first independent replicate experiment (-1) was used to present in the manuscript, which related to Fig. 7b.

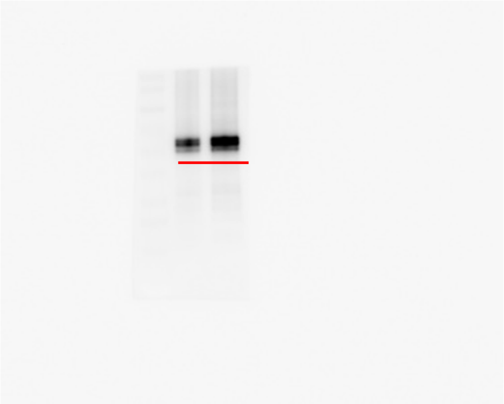

Cyclin B1-Control-H.B-2

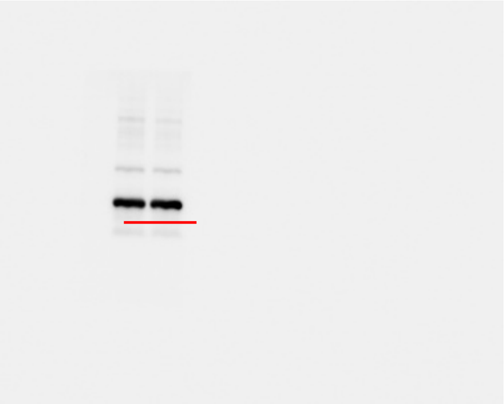

GAPDH-Control-H.B-2

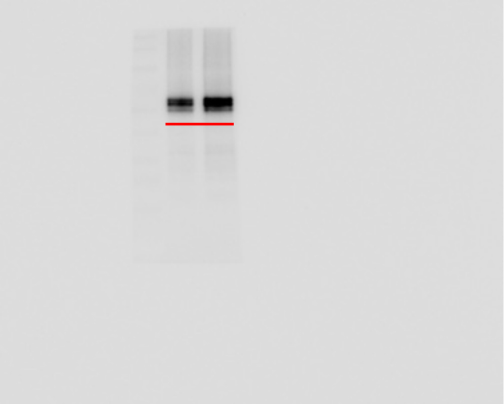

Cyclin B1-Control-H.B-3

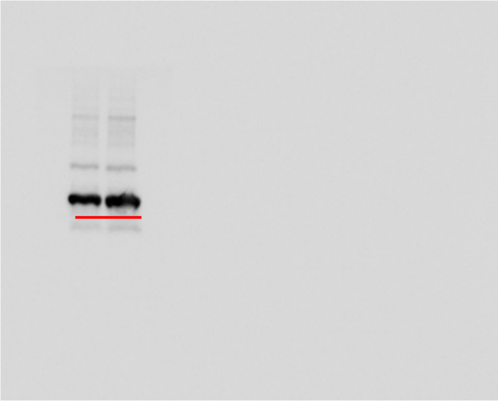

GAPDH-Control-H.B-3

Cyclin B1 expression level after Halorotetin B treatment, related to Fig. 7b.

Securin Expression

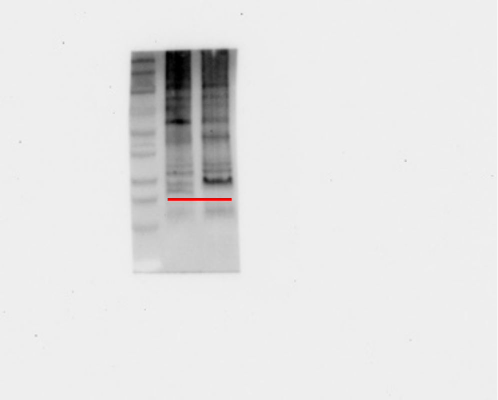

Securin-Control-H.B-1

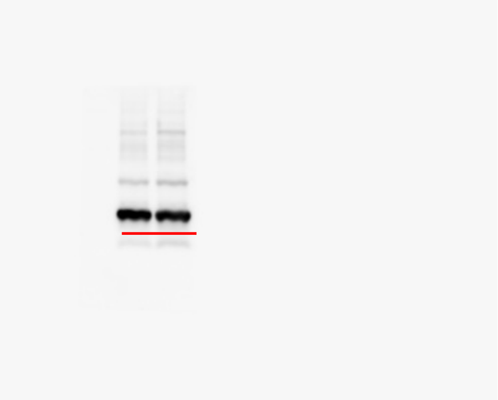

GAPDH-Control-H.B-1

The first independent replicate experiment (-1) was used to present in the

manuscript, which related to Fig. 7b.

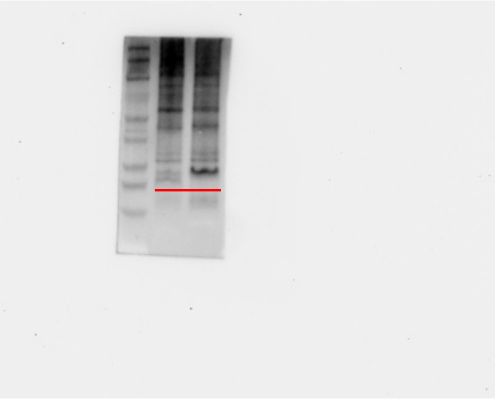

Securin-Control-H.B-2

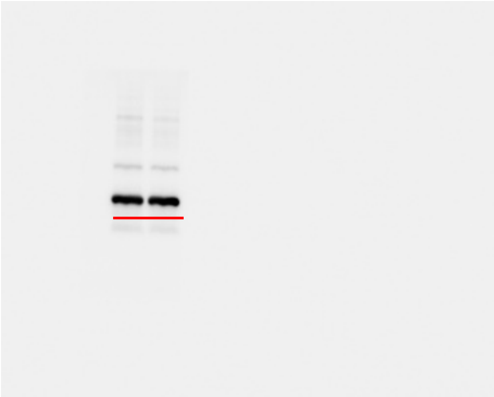

GAPDH-Control-H.B-2

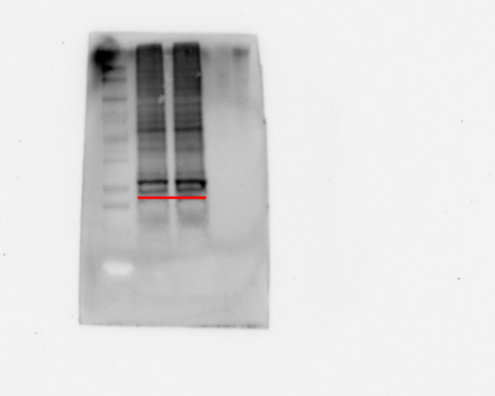

Securin-Control-H.B-3

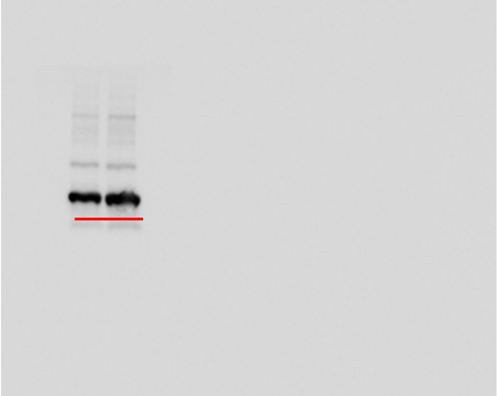

GAPDH-Control-H.B-3

Securin expression level after Halorotetin B treatment, related to Fig. 7b.

MG-132-Cyclin B1 Expression

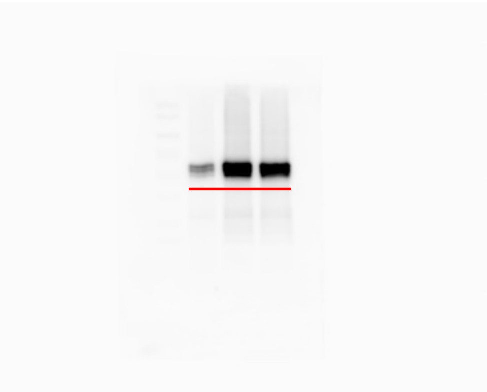

Cyclin B1-MG-132-0, 1 μM, 10 μM-1

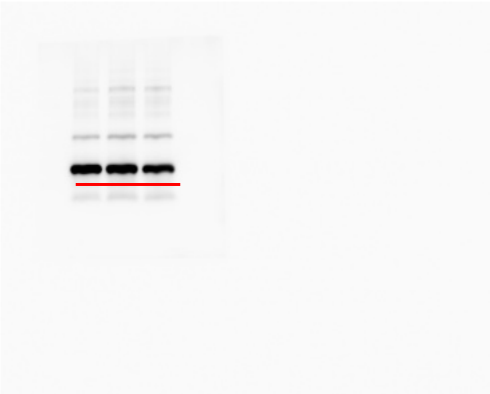

GAPDH-MG-132-0, 1 μM, 10 μM-1

The first independent replicate experiment (-1) was used to present in the manuscript, which related to Fig. 7c.

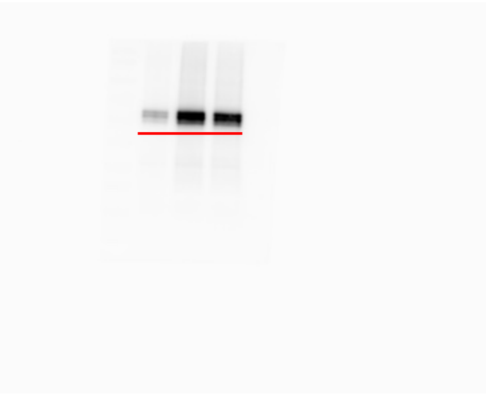

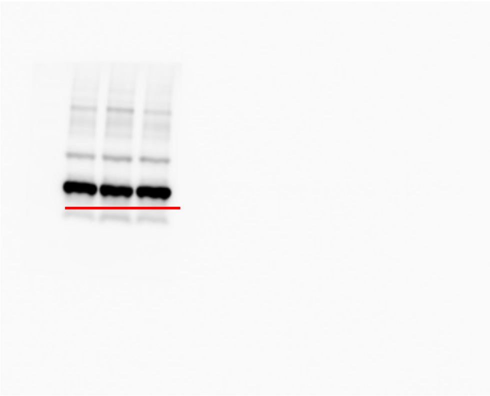

Cyclin B1-MG-132-0, 1 μM, 10 μM-2 GAPDH-MG-132-0, 1 μM, 10 μM-2

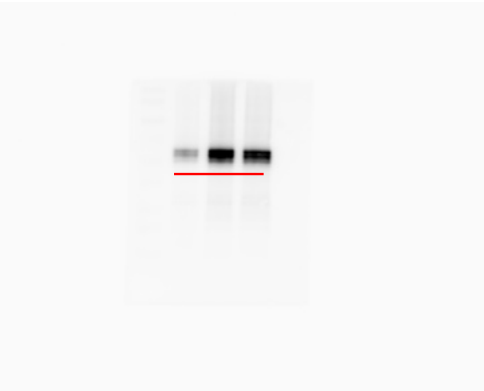

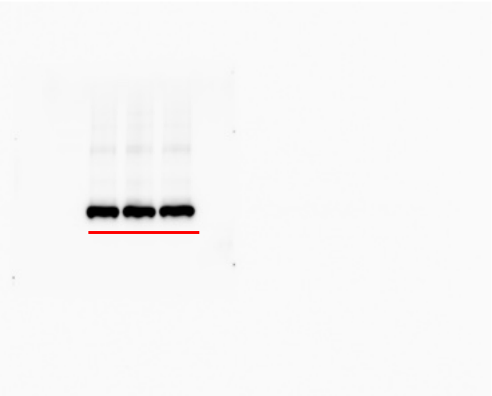

Cyclin B1-MG-132-0, 1 μM, 10 μM-3 GAPDH-MG-132-0, 1 μM, 10 μM-3

Cyclin B1 expression level after MG-132 treatment, related to Fig. 7c.

MG-132-Securin Expression

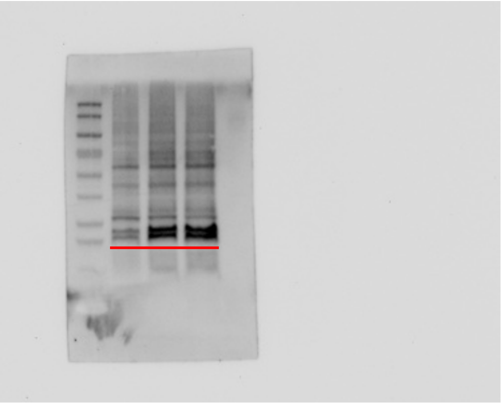

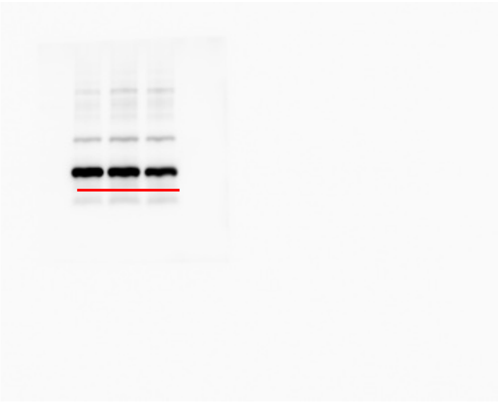

Securin-MG-132-0, 1 μM, 10 μM-1 GAPDH-MG-132-0, 1 μM, 10 μM-1

The first independent replicate experiment (-1) was used to present in the manuscript, which related to Fig. 7c.

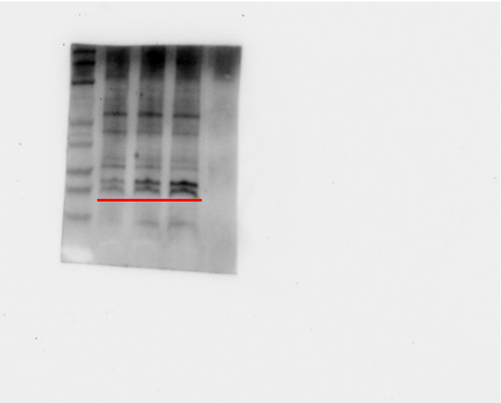

Securin-MG-132-0, 1 μM, 10 μM-2

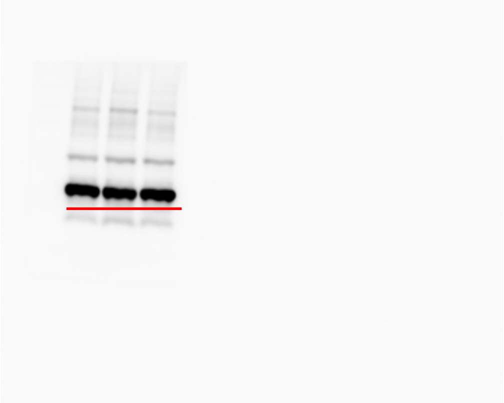

GAPDH-MG-132-0, 1 μM, 10 μM-2

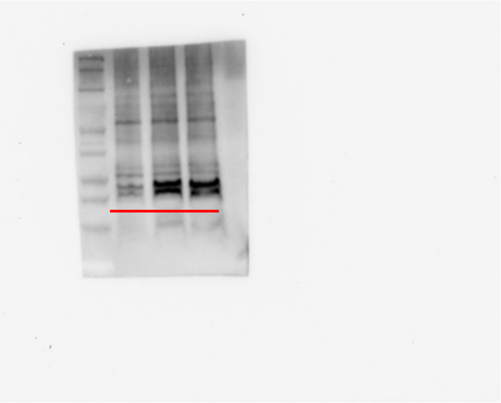

Securin-MG-132-0, 1 μM, 10 μM-3

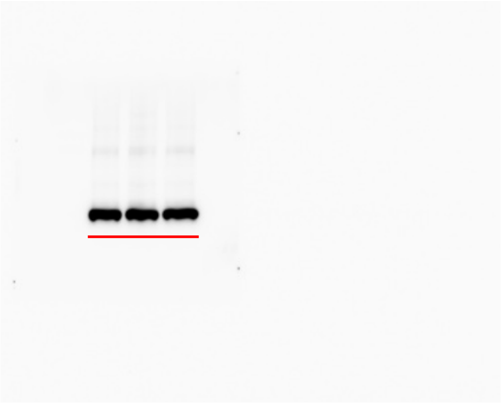

GAPDH-MG-132-0, 1 μM, 10 μM-3

Securin expression level after MG-132 treatment, related to Fig. 7c.

CHX-Cyclin B1 Degradation Rate

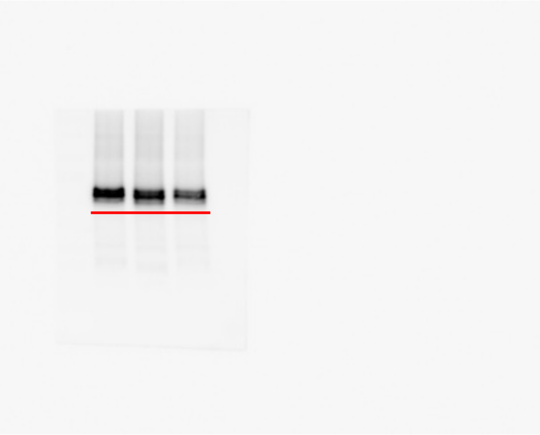

CHX-4 h, 8 h, 12 h-Cyclin B1-Control-1

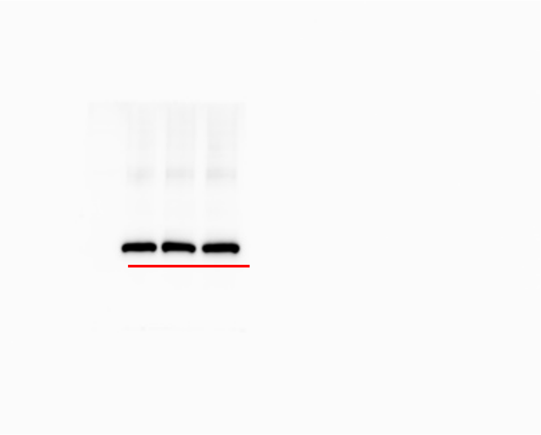

CHX-4 h, 8 h, 12 h-GAPDH-Control-1

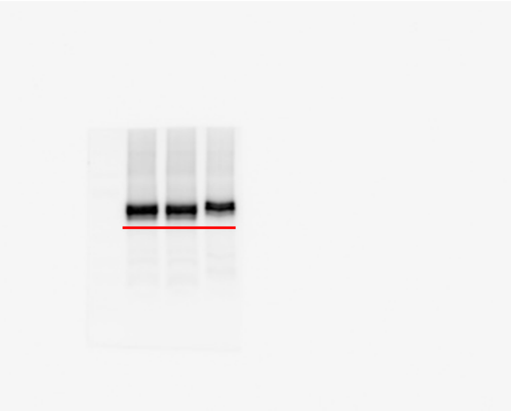

CHX-4 h, 8 h, 12 h-Cyclin B1-H.B-1

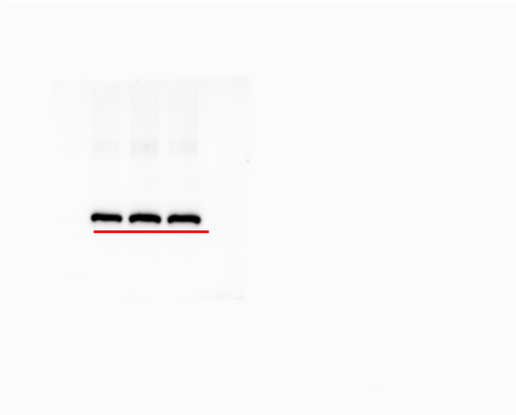

CHX-4 h, 8 h, 12 h-GAPDH-H.B-1

The first independent replicate experiment (-1) was used to present in the manuscript, which related to Fig. 7d.

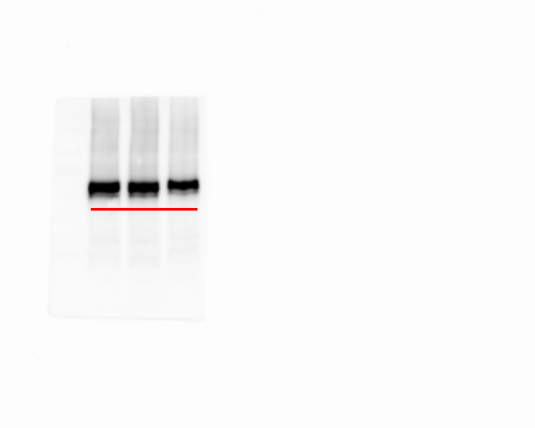

CHX-4 h, 8 h, 12 h-Cyclin B1-Control-2

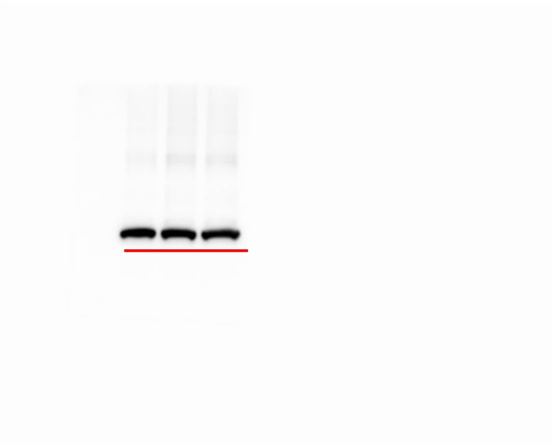

CHX-4 h, 8 h, 12 h-GAPDH-Control-2

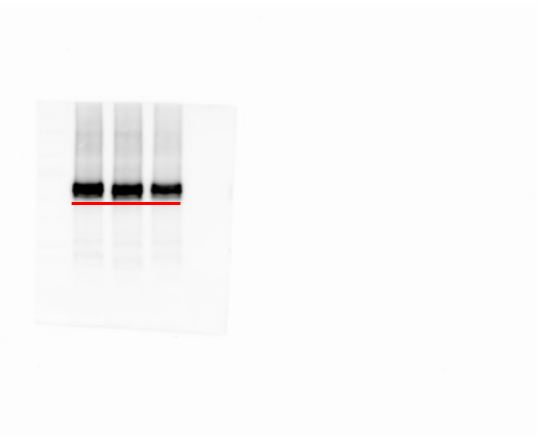

CHX-4 h, 8 h, 12 h-Cyclin B1-H.B-2

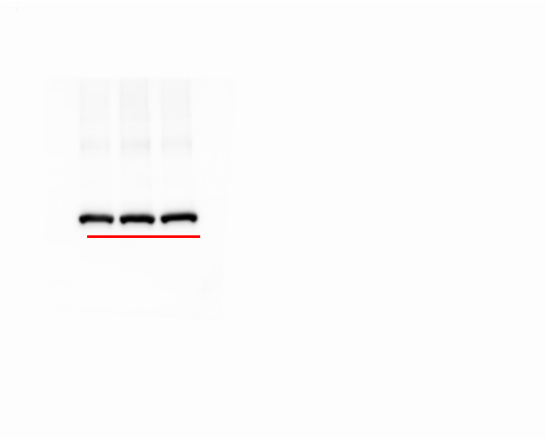

CHX-4 h, 8 h, 12 h-GAPDH-H.B-2

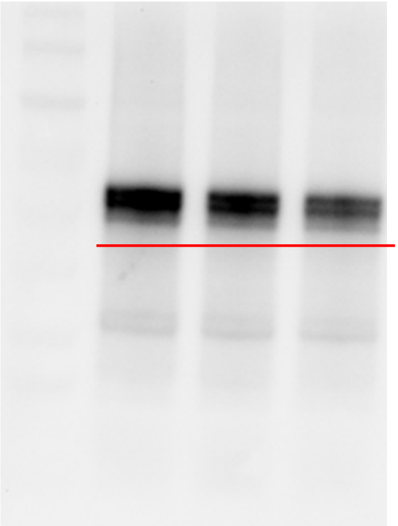

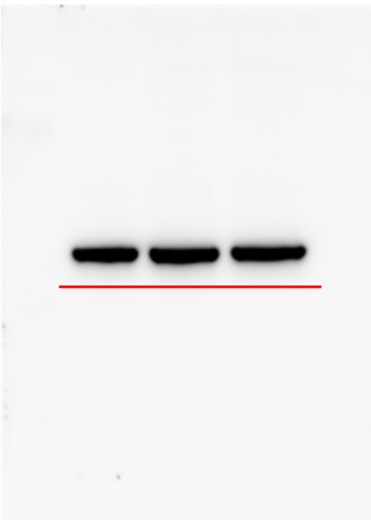

CHX-4 h, 8 h, 12 h-Cyclin B1-Control-3

CHX-4 h, 8 h, 12 h-GAPDH-Control-3

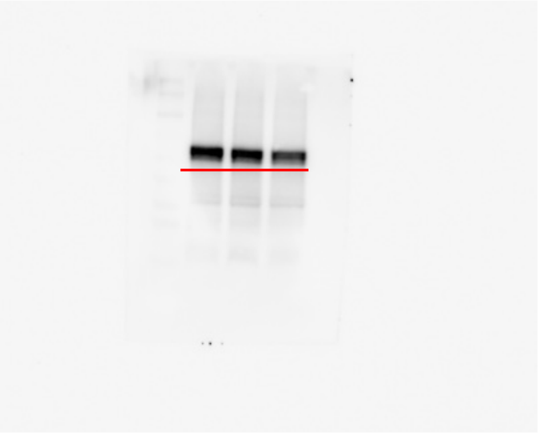

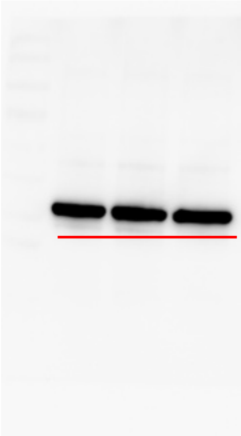

CHX-4 h, 8 h, 12 h-Cyclin B1-H.B-3

CHX-4 h, 8 h, 12 h-GAPDH-H.B-3

Cyclin B1 degradation rate after Halorotetin B treatment, related to Fig. 7d.

CHX-Securin Degradation Rate

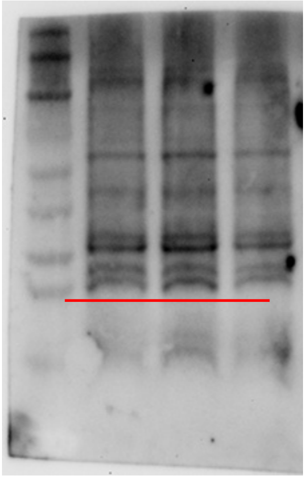

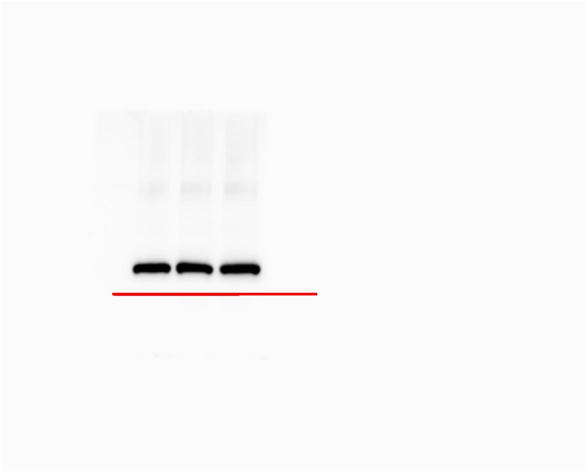

CHX-4 h, 8 h, 12 h-Securin-Control-1   CHX-4 h, 8 h, 12 h-GAPDH-Control-1

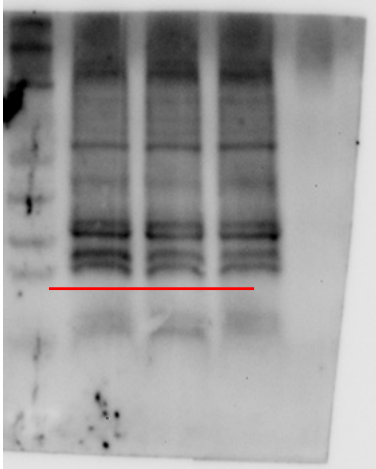

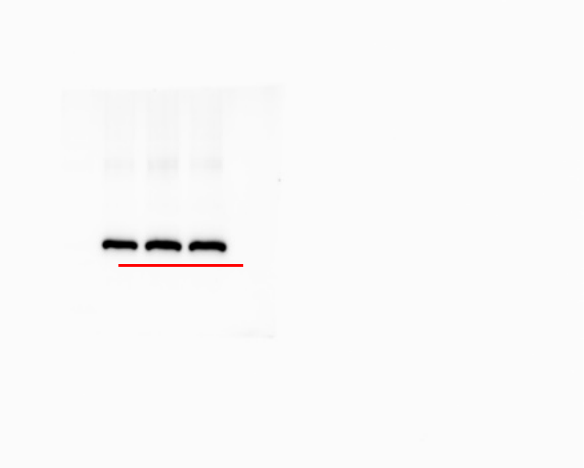

CHX-4 h, 8 h, 12 h-Securin-H.B-1   CHX-4 h, 8 h, 12 h-GAPDH-H.B-1

The first independent replicate experiment (-1) was used to present in the manuscript, which related to Fig. 7d.

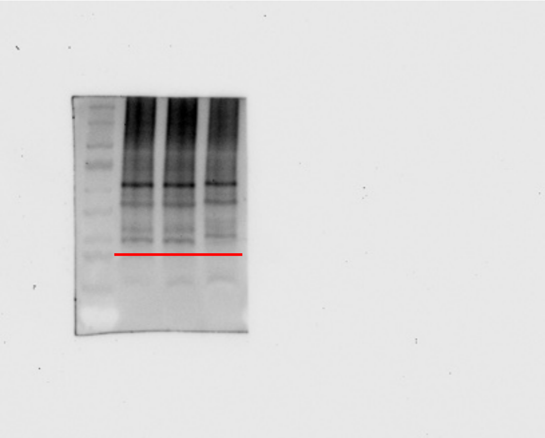

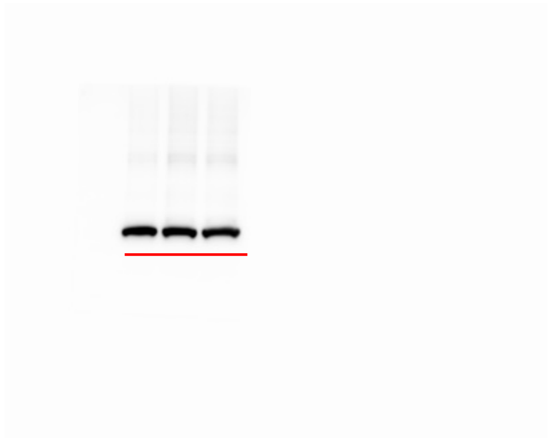

CHX-4 h, 8 h, 12 h-Securin-Control-2   CHX-4 h, 8 h, 12 h-GAPDH-Control-2

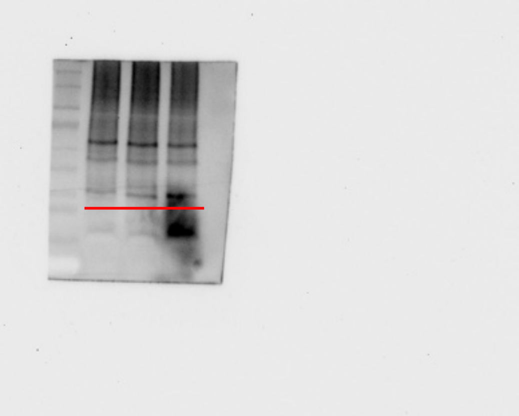

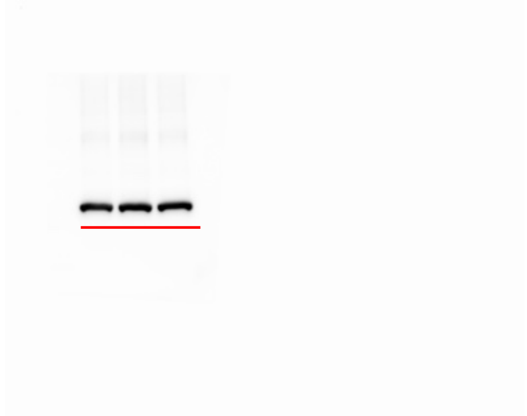

CHX-4 h, 8 h, 12 h-Securin-H.B-2   CHX-4 h, 8 h, 12 h-GAPDH-H.B-2

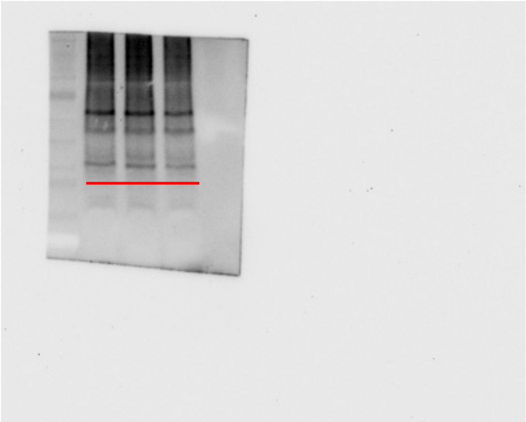

CHX-4 h, 8 h, 12 h-Securin-Control-3

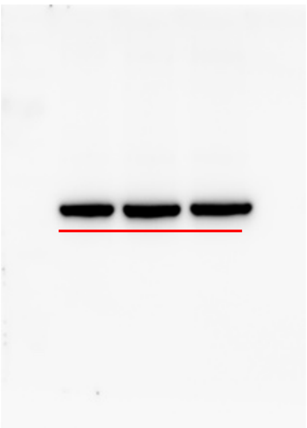

CHX-4 h, 8 h, 12 h-GAPDH-Control-3

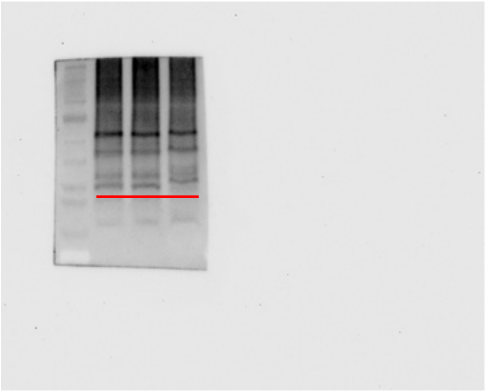

CHX-4 h, 8 h, 12 h-Securin-H.B-3

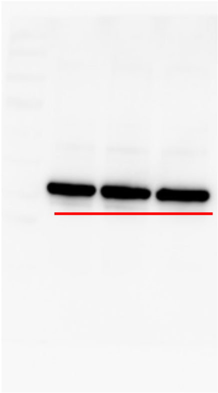

CHX-4 h, 8 h, 12 h-GAPDH-H.B-3

Securin degradation rate after Halorotetin B treatment, related to Fig. 7d.

Ubiquitin-Input-Cyclin B1 and Securin

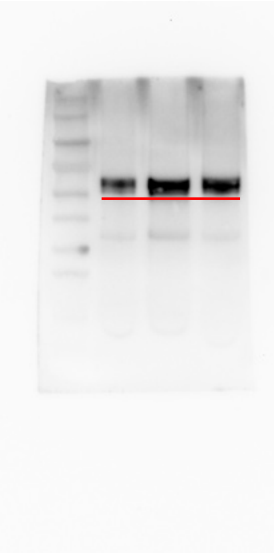
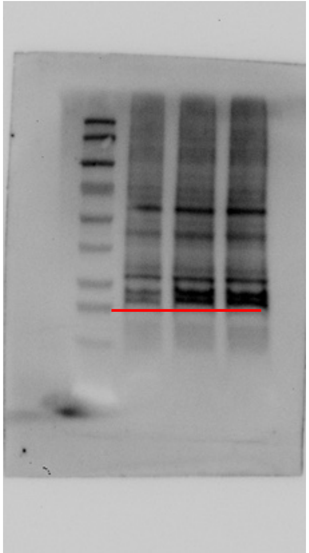
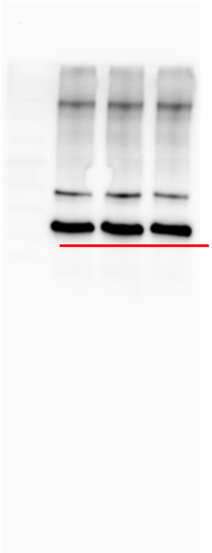

Input-Cyclin B1-1   Input-Securin-1   Input-GAPDH-1

The first independent replicate experiment (-1) was used to present in the manuscript for the input of cyclin B1 and securin, which related to Fig. 7e.

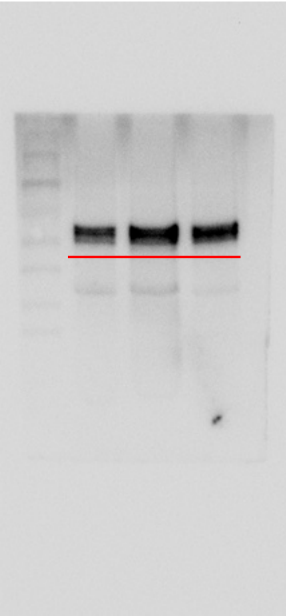
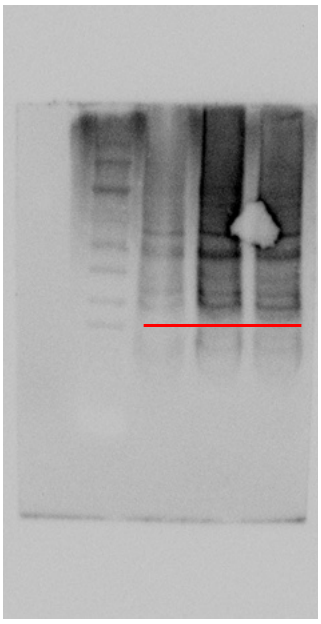
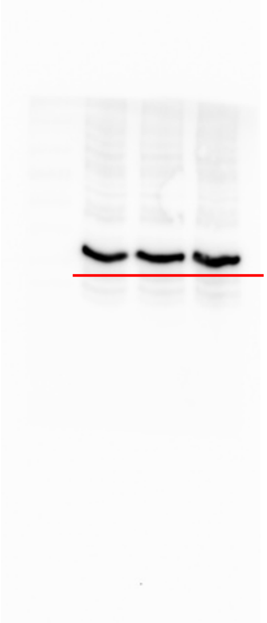

Input-Cyclin B1-2   Input-Securin-2   Input-GAPDH-2

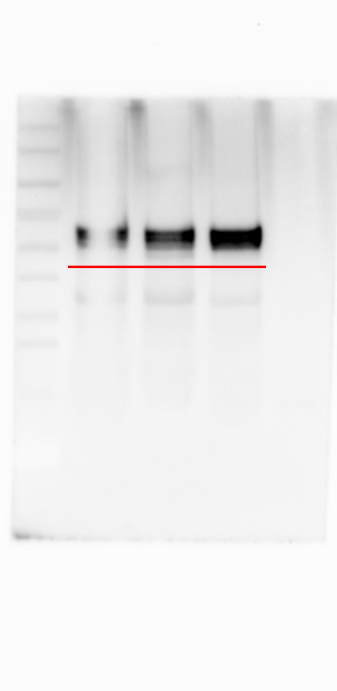

Input-Cyclin B1-3

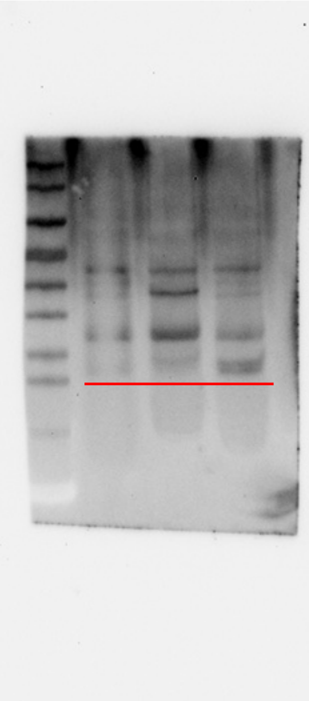

Input-Securin-3

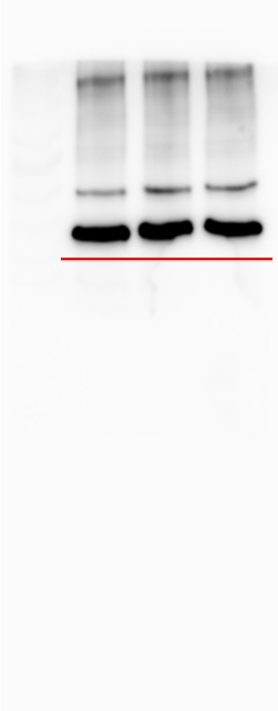

Input-GAPDH-3

Cyclin B1 and Securin expression level after Halorotetin B and MG-132 treatment, related to Fig. 7e.

Ubiquitin-IP-Cyclin B1

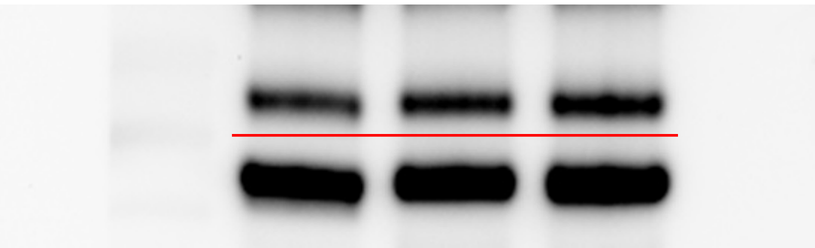

IP-Cyclin B1-1

The first independent replicate experiment (-1) was used to present in the manuscript, which related to Fig. 7e.

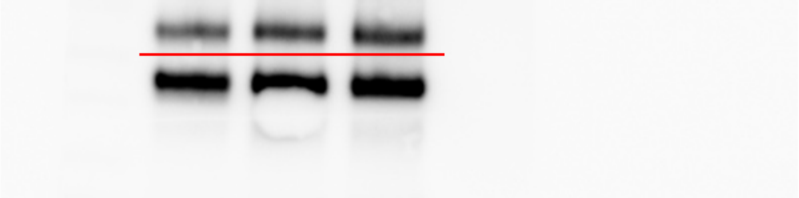

IP-Cyclin B1-2

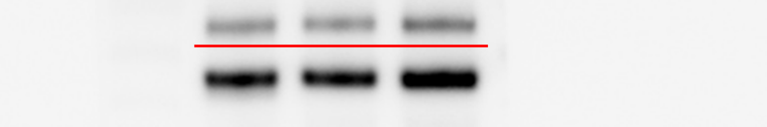

IP-Cyclin B1-3

Cyclin B1 level after rProtein G MagPoly beads treatment, related to Fig. 7e.

Ubiquitin-IP-Securin

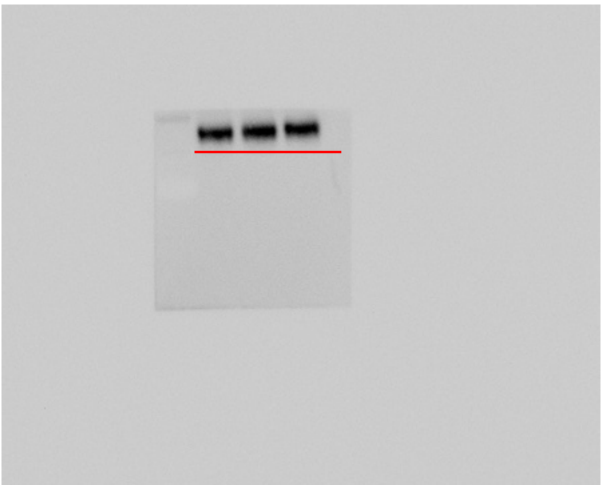

IP-Securin-1

The first independent replicate experiment (-1) was used to present in the manuscript, which related to Fig. 7e.

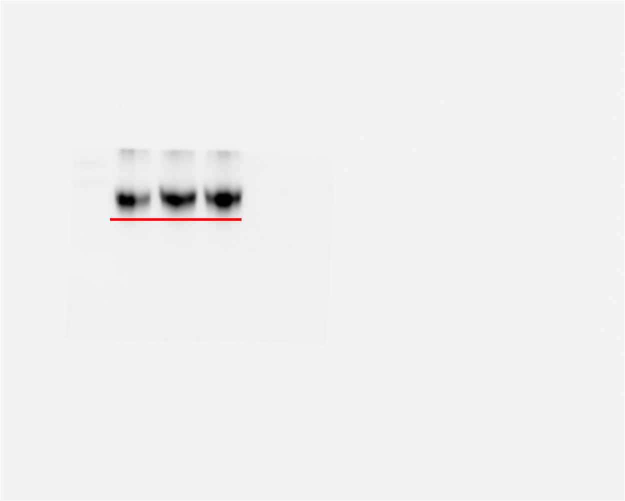

IP-Securin-2

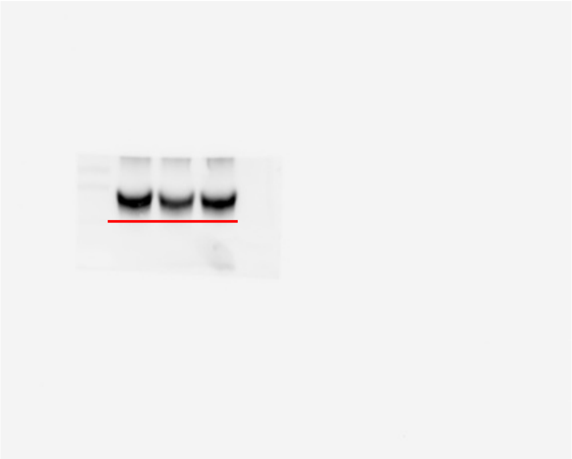

IP-Securin-3

Securin level after rProtein G MagPoly beads treatment, related to Fig. 7e.

Ubiquitin-Cyclin B1

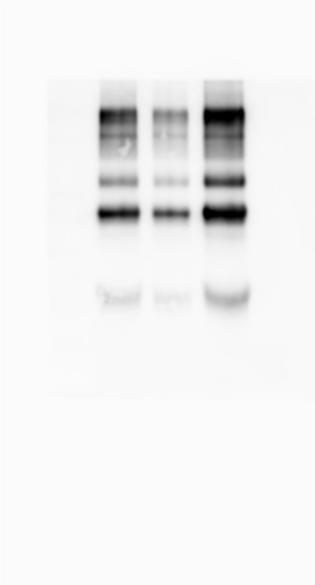

Ubiquitin-Cyclin B1-1

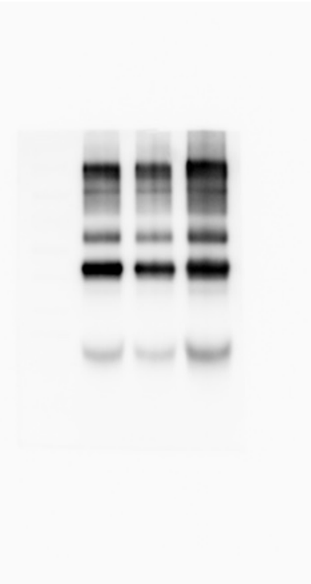

Ubiquitin-Cyclin B1-2

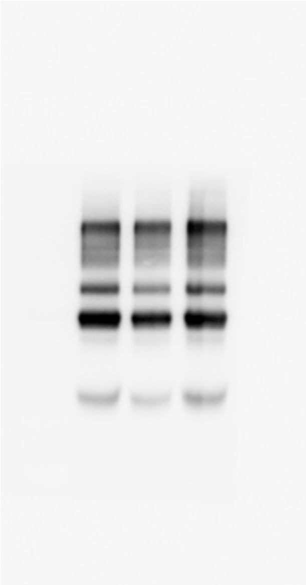

Ubiquitin-Cyclin B1-3

The first independent replicate experiment (-1) was used to present in the manuscript, which related to Fig. 7e.

Ubiquitin level detection of Cyclin B1 after Halorotetin B and MG-132 treatment, related to Fig. 7e.

Ubiquitin-Securin

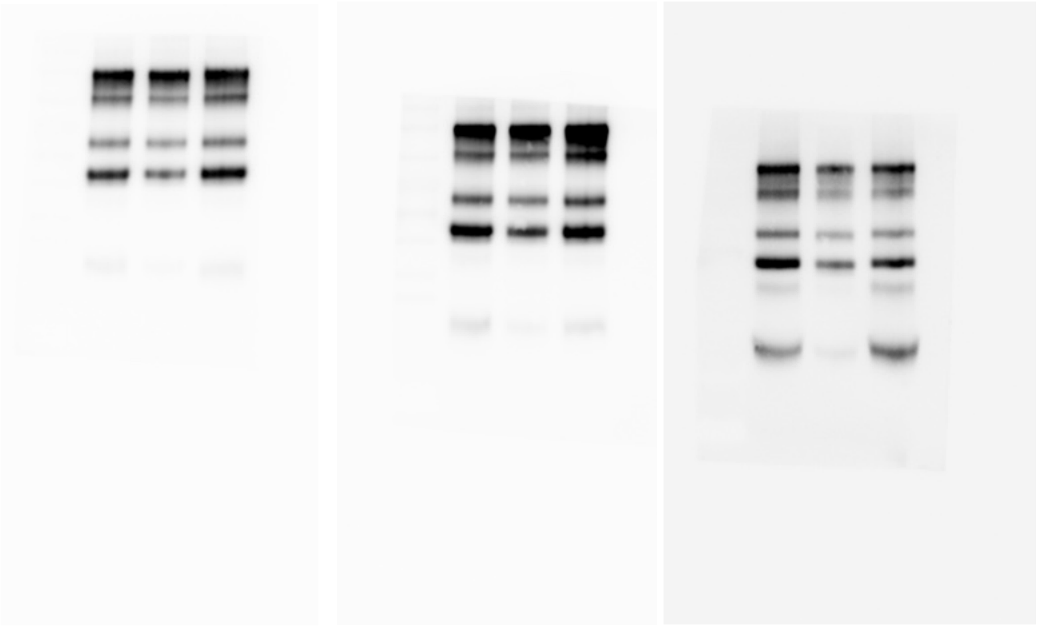

Ubiquitin-Securin-1   Ubiquitin-Securin-2   Ubiquitin-Securin-3

The first independent replicate experiment (-1) was used to present in the manuscript, which related to Fig. 7e.

Ubiquitin level detection of Securin after Halorotetin B and MG-132 treatment, related to Fig. 7e.

Cyclin B1 and Securin protein level in tumor tissue

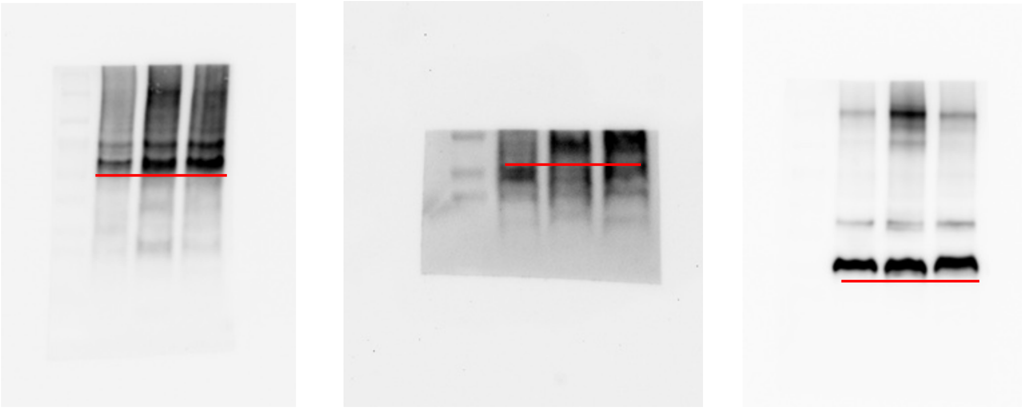

Cyclin B1-Control-10 mg/kg-20 mg/kg-1   Securin-Control-10 mg/kg-20 mg/kg-1   GAPDH-Control-10 mg/kg-20 mg/kg-1

The first independent replicate experiment (-1) was used to present in the manuscript, which related to Fig. S8a.

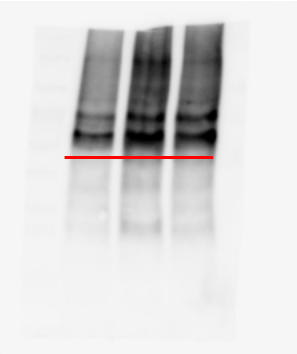

Cyclin B1-Control-
10 mg/kg-20 mg/kg-2

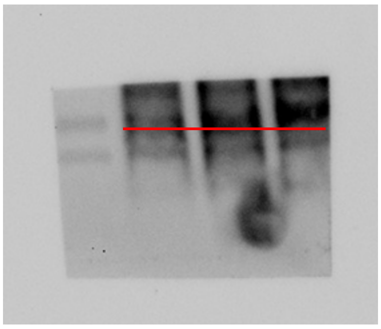

Securin-Control-
10 mg/kg-20 mg/kg-2

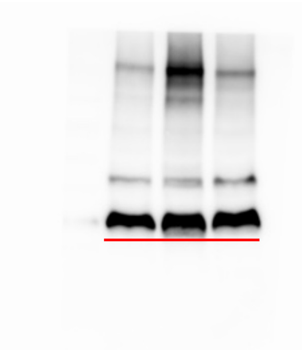

GAPDH-Control-
10 mg/kg-20 mg/kg-2

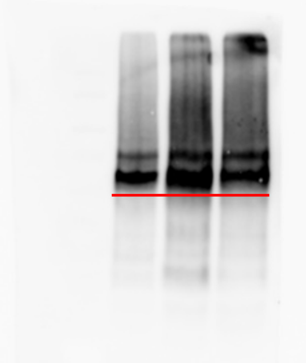

Cyclin B1-Control-
10 mg/kg-20 mg/kg-3

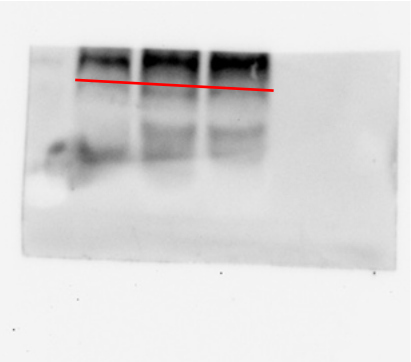

Securin-Control-
10 mg/kg-20 mg/kg-3

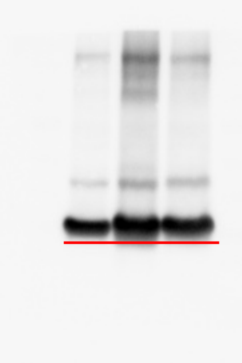

GAPDH-Control-
10 mg/kg-20 mg/kg-3

Protein level of cyclin B1 and securin in tumor tissues after treatment with Halorotetin B or solvent, related to Fig. S8a.

Proteins of cell senescence

HepG2 cell

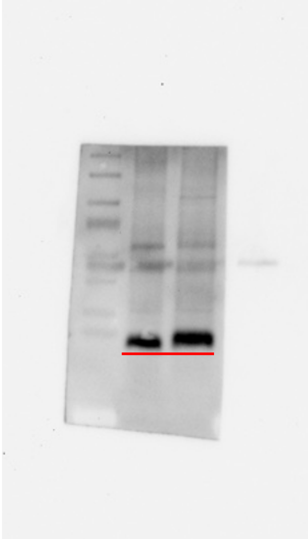

HepG2-Bcl-2-1

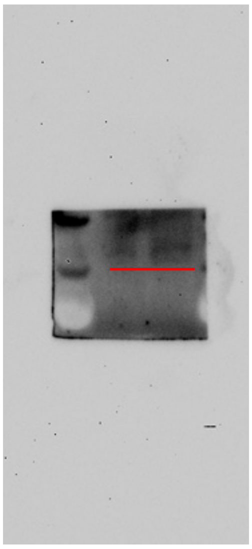

HepG2-p21-1

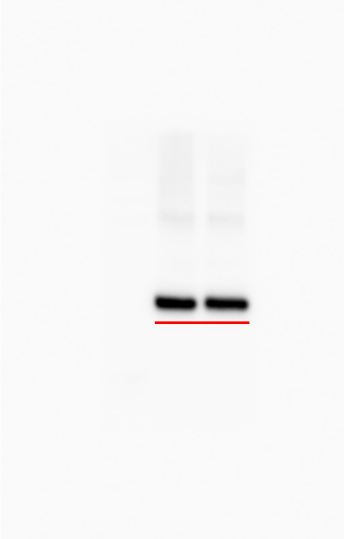

HepG2-GAPDH-1

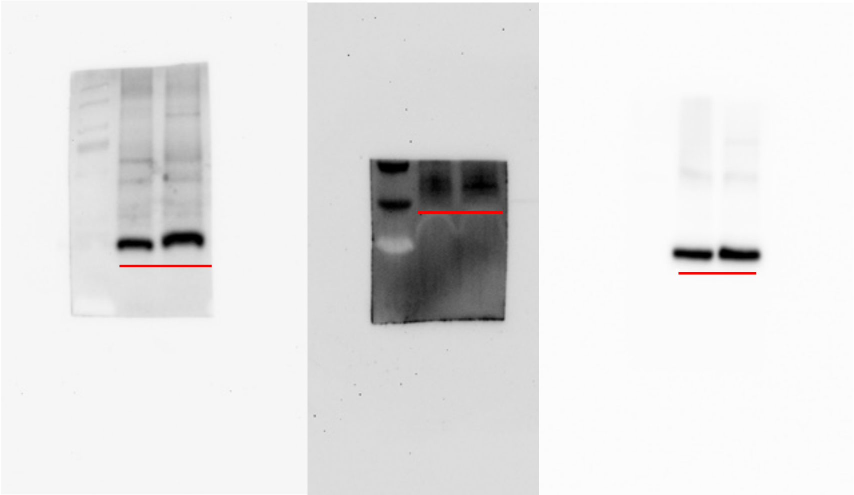

HepG2-Bcl-2-2 HepG2-p21-2 HepG2-GAPDH-2

The second independent replicate experiment (-2) was used to present in the manuscript, which related to Fig. 8b.

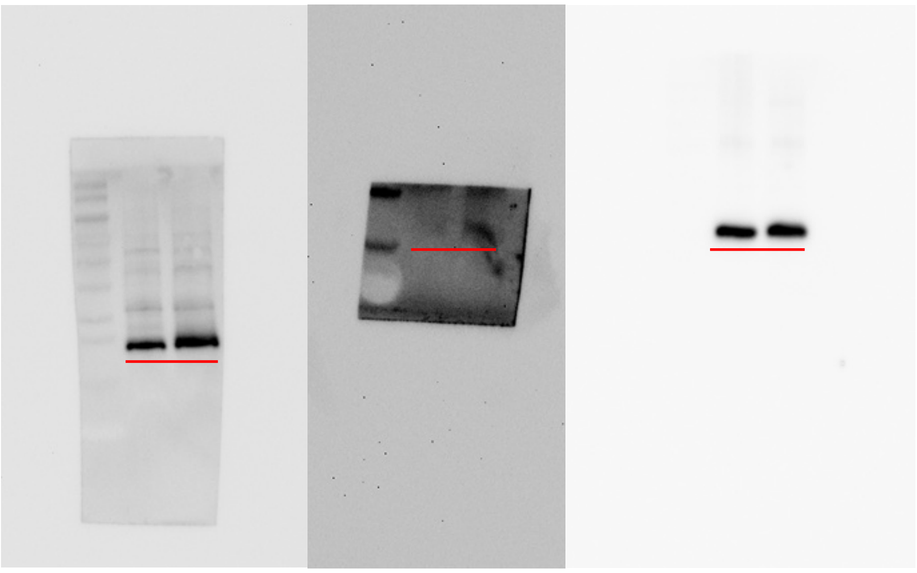

HepG2-Bcl-2-3 HepG2-p21-3 HepG2-GAPDH-3

Bel-7402 cell

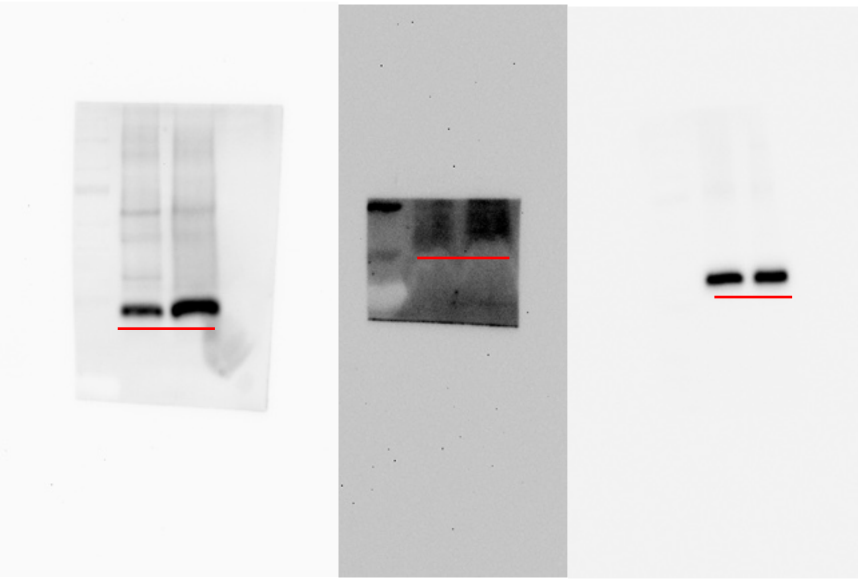

Bel-7402-Bcl-2-1 Bel-7402-p21-1 Bel-7402-GAPDH-1

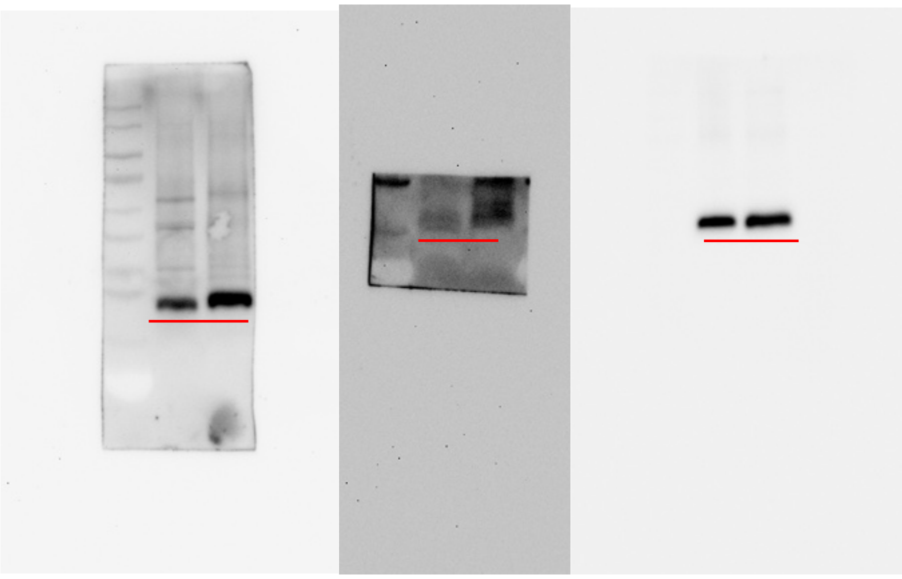

Bel-7402-Bcl-2-2 Bel-7402-p21-2 Bel-7402-GAPDH-2

The second independent replicate experiment (-2) was used to present in the manuscript, which related to Fig. 7c.

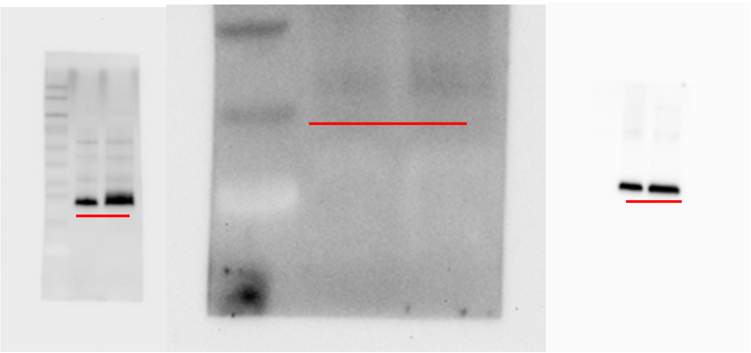

Bel-7402-Bcl-2-3 Bel-7402-p21-3 Bel-7402-GAPDH-3

Huh-7 cell

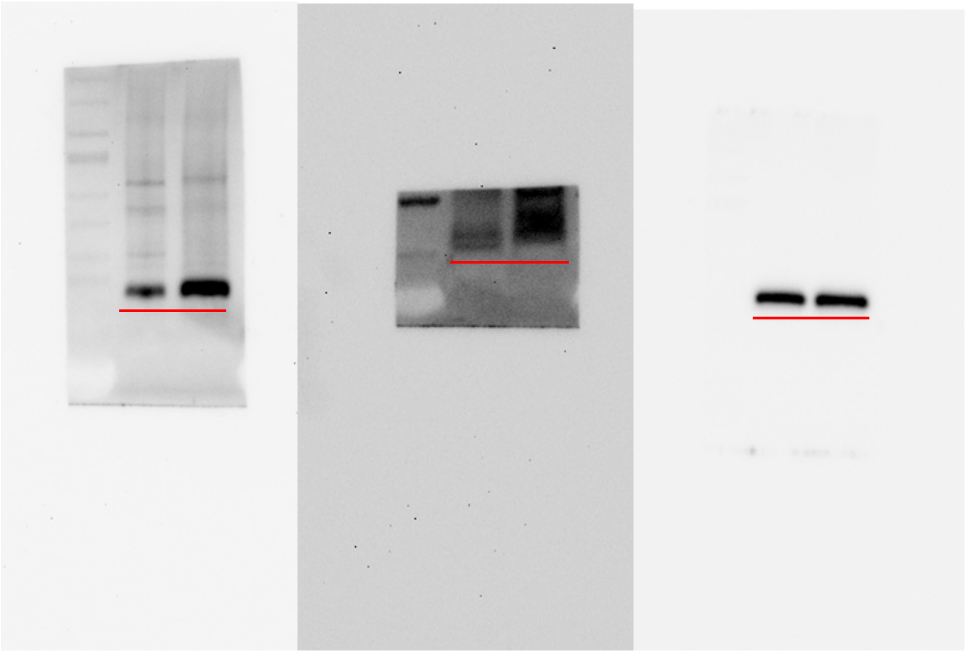

Huh-7-Bcl-2-1 Huh-7-p21-1 Huh-7-GAPDH-1

The first independent replicate experiment (-1) was used to present in the manuscript, which related to Fig. S9b.

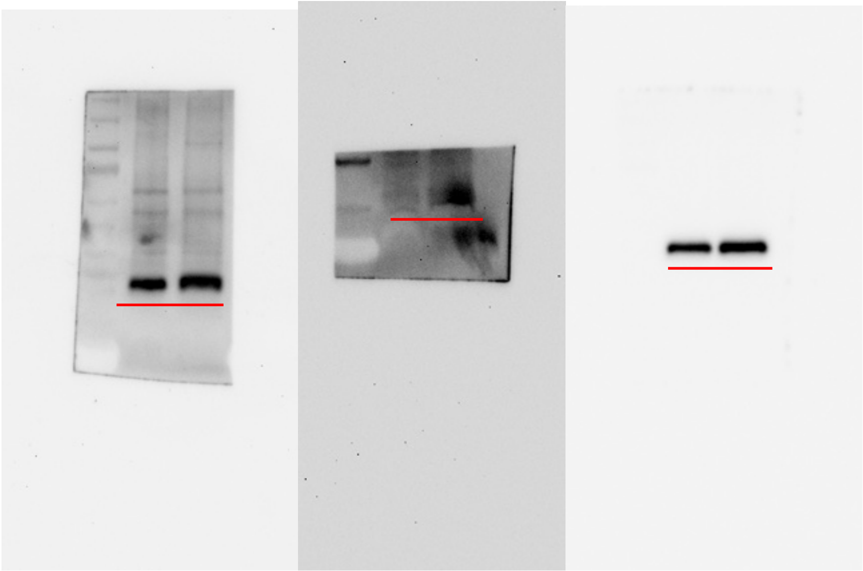

Huh-7-Bcl-2-2 Huh-7-p21-2 Huh-7-GAPDH-2

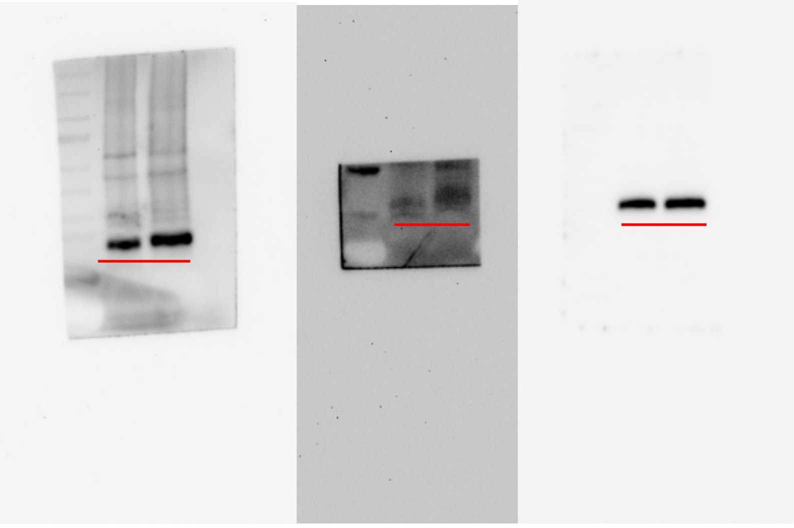

Huh-7-Bcl-2-3 Huh-7-p21-3 Huh-7-GAPDH-3

Bcl-2 and p21 expression level after Halorotetin B treatment, related to Fig. 8b and S9b.

SASP detection

HepG2 cell

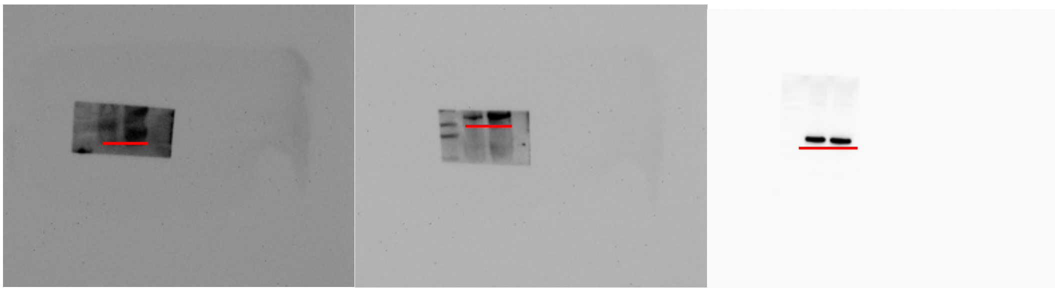

HepG2-IL-1β-1 HepG2-IL-6-1 HepG2-GAPDH-1

The first independent replicate experiment (-1) was used to present in the manuscript, which related to Fig. 8c.

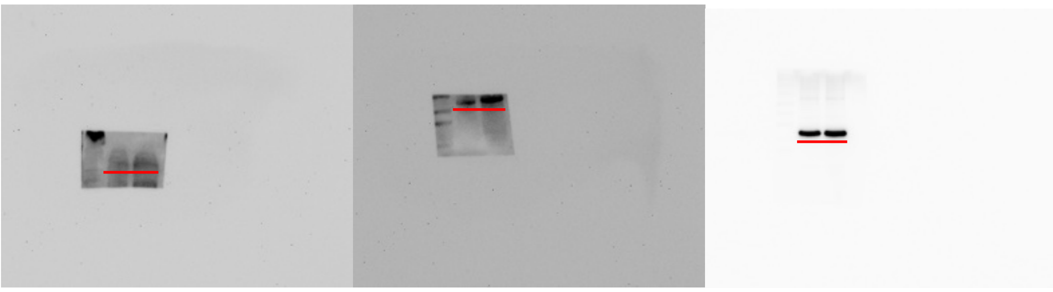

HepG2-IL-1β-2 HepG2-IL-6-2 HepG2-GAPDH-2

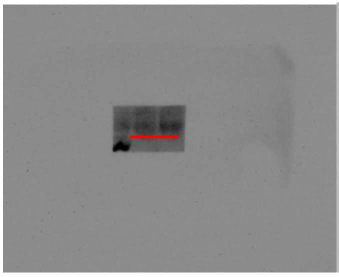
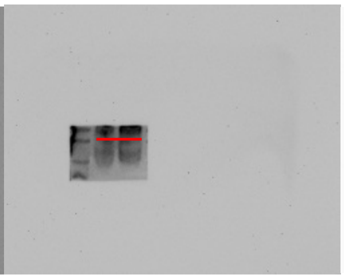
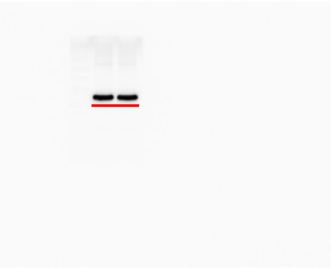

HepG2-IL-1β-3 HepG2-IL-6-3 HepG2-GAPDH-3

Bel-7402 cell

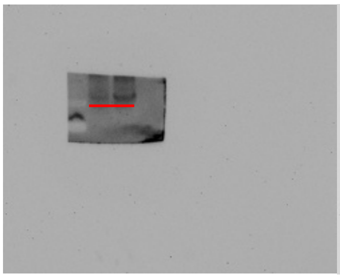
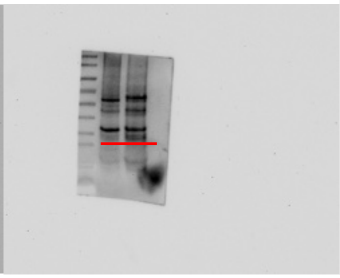
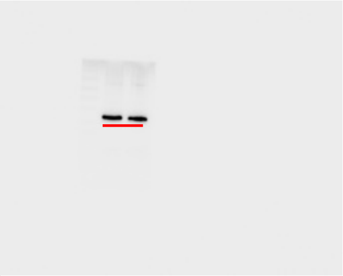

Bel-7402-IL-1β-1 Bel-7402-IL-6-1 Bel-7402-GAPDH-1

The first independent replicate experiment (-1) was used to present in the manuscript, which related to Fig. 8c.

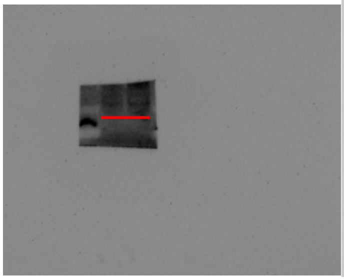
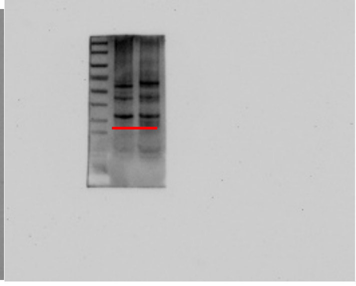
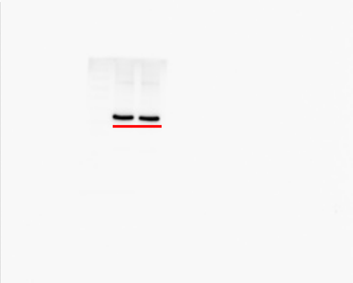

Bel-7402-IL-1β-2 Bel-7402-IL-6-2 Bel-7402-GAPDH-2

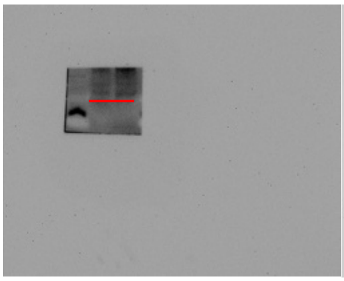
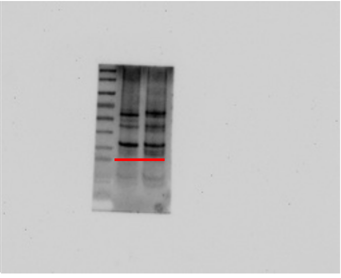

Bel-7402-IL-1β-3 Bel-7402-IL-6-3 Bel-7402-GAPDH-3

Huh-7 cell

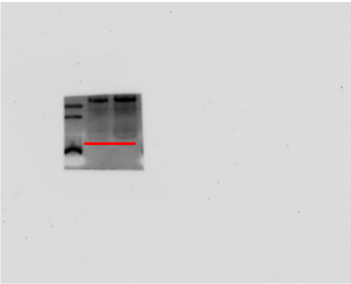

Huh-7-IL-1β-1

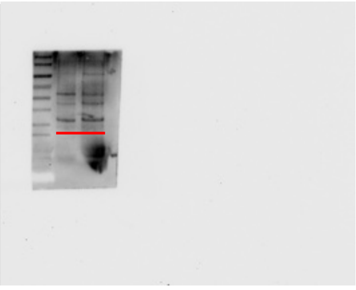

Huh-7-IL-6-1

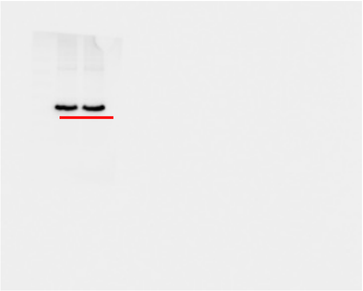

Huh-7-GAPDH-1

The first independent replicate experiment (-1) was used to present in the manuscript, which related to Fig. S9c.

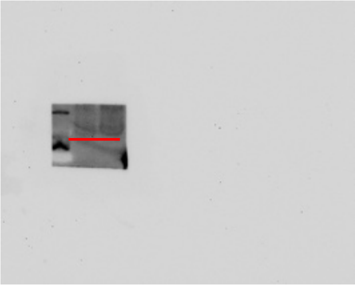

Huh-7-IL-1β-2

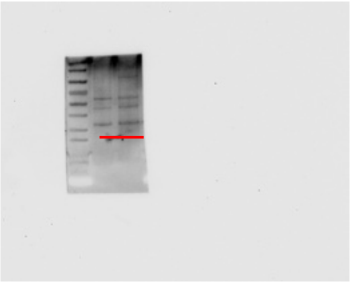

Huh-7-IL-6-2

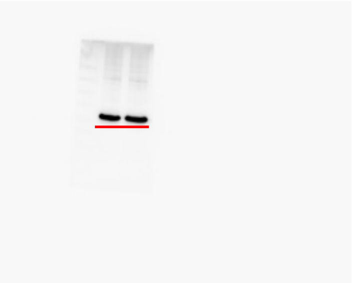

Huh-7-GAPDH-2

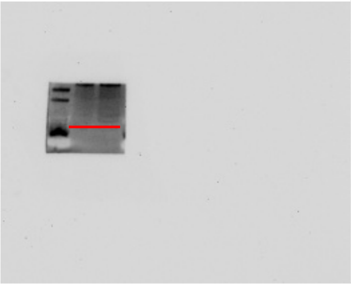

Huh-7-IL-1β-3

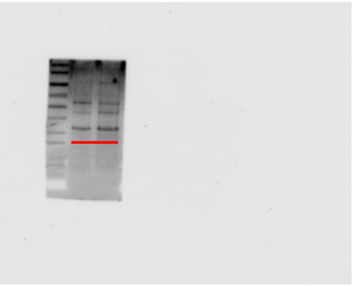

Huh-7-IL-6-3

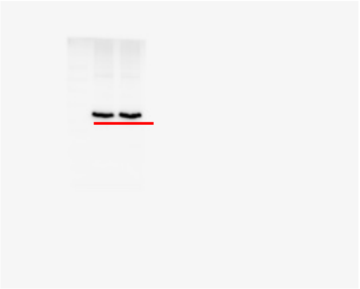

Huh-7-GAPDH-3

SASP detection after Halorotetin B treatment, related to Fig. 8c and S9c.
